# Early midcell localization of *Escherichia coli* PBP4 supports the function of peptidoglycan amidases

**DOI:** 10.1371/journal.pgen.1010222

**Published:** 2022-05-23

**Authors:** Jolanda Verheul, Adam Lodge, Hamish C. L. Yau, Xiaolong Liu, Gabriela Boelter, Xinwei Liu, Alexandra S. Solovyova, Athanasios Typas, Manuel Banzhaf, Waldemar Vollmer, Tanneke den Blaauwen

**Affiliations:** 1 Bacterial Cell Biology, Swammerdam Institute for Life Sciences, Faculty of Science, University of Amsterdam, Amsterdam, The Netherlands; 2 Centre for Bacterial Cell Biology, Biosciences Institute, Newcastle University, Newcastle upon Tyne, United Kingdom; 3 Department of Biochemistry, University of Oxford, Oxford, United Kingdom; 4 Institute of Microbiology & Infection and School of Biosciences, University of Birmingham, Edgbaston, Birmingham, United Kingdom; 5 NUPPA, Leech Building, Framlington Place, Newcastle upon Tyne, United Kingdom; 6 European Molecular Biology Laboratory, Genome Biology Unit, Heidelberg, Germany; 7 European Molecular Biology Laboratory, Structural & Computational Unit, Heidelberg, Germany; Washington University in St. Louis, UNITED STATES

## Abstract

Insertion of new material into the *Escherichia coli* peptidoglycan (PG) sacculus between the cytoplasmic membrane and the outer membrane requires a well-organized balance between synthetic and hydrolytic activities to maintain cell shape and avoid lysis. Since most bacteria carry multiple enzymes carrying the same type of PG hydrolytic activity, we know little about the specific function of given enzymes. Here we show that the DD-carboxy/endopeptidase PBP4 localizes in a PBP1A/LpoA and FtsEX dependent fashion at midcell during septal PG synthesis. Midcell localization of PBP4 requires its non-catalytic domain 3 of unknown function, but not the activity of PBP4 or FtsE. Microscale thermophoresis with isolated proteins shows that PBP4 interacts with NlpI and the FtsEX-interacting protein EnvC, an activator of amidases AmiA and AmiB, which are needed to generate denuded glycan strands to recruit the initiator of septal PG synthesis, FtsN. The domain 3 of PBP4 is needed for the interaction with NlpI and EnvC, but not PBP1A or LpoA. *In vivo* crosslinking experiments confirm the interaction of PBP4 with PBP1A and LpoA. We propose that the interaction of PBP4 with EnvC, whilst not absolutely necessary for mid-cell recruitment of either protein, coordinates the activities of PBP4 and the amidases, which affects the formation of denuded glycan strands that attract FtsN. Consistent with this model, we found that the divisome assembly at midcell was premature in cells lacking PBP4, illustrating how the complexity of interactions affect the timing of cell division initiation.

## Author summary

Peptidoglycan biosynthesis is a major target for antibacterials. The covalently closed peptidoglycan mesh, called sacculus, protects the bacterium from lysis due to its turgor. Sacculus growth is facilitated by the balanced activities of synthases and hydrolases, and disturbing this balance leads to cell lysis and bacterial death. Because of the large number and possible redundant functions of peptidoglycan hydrolases, it has been difficult to decipher their individual functions. In this paper we show that the DD-endopeptidase PBP4 localizes at midcell during septal peptidoglycan synthesis in *Escherichia coli* and is involved in the timing of the assembly and activation of the division machinery. This shows that inhibition of certain hydrolases could weaken the cells and might enhance antibiotic action.

## Introduction

The peptidoglycan (PG) layer is sandwiched between the cytoplasmic membrane (CM) and the outer membrane (OM) of the Gram-negative bacterium *Escherichia coli* forming a covalently closed network of glycan strands that are interconnected by short peptides [1]. The PG layer maintains the shape of the bacterium by stabilizing the cell against its high internal osmotic pressure. To proliferate, the rod-shaped bacterium grows in length and then divides by binary fission into two equally sized daughter cells, with nascent PG inserted into the lateral cell wall and septum.

The basic precursor (lipid II) for PG synthesis is the β-(1,4) linked disaccharide *N*-acetylglucosamine-*N*-acetylmuramic acid (Glc*N*Ac-Mur*N*Ac) with an undecaprenol-pyrophosphate linked to C1 of Mur*N*Ac and a stem peptide with the sequence L-alanine-D-*iso*-glutamate-diaminopimelic acid (Dap)-D-alanine-D-alanine coupled to the C3 of Mur*N*Ac. Lipid II is used by glycosyltransferases (GTases), which polymerize the glycan chains, and TPases, which crosslink the stem peptides. The main PG synthases in *E*. *coli* are the class A PBPs (PBP1A and PBP1B) with GTase and TPase activities [2–6], the integral membrane proteins RodA and FtsW with GTase activity [7–9] and their partner TPases PBP2 and PBP3, respectively [10–13].

Binary fission in *E*. *coli* is initiated by the assembly of the Z-ring at midcell, which consists of a network of dynamic FtsZ filaments that are anchored to the cytoplasmic membrane by FtsA and ZipA [14,15]. The Z-ring also recruits the PG synthases PBP1A and PBP1B to midcell [2,16]. A number of Z-associated proteins play a role in the organization and dynamics of the ring [17–22]. Of these, the integral membrane protein FtsX interacts with FtsA [23] and its cytoplasmic ATPase partner protein FtsE interacts with FtsZ [24]. FtsE and FtsX localize early to midcell, together with the Z-ring [25]. The recruitment of the other cell division proteins occurs with a time delay of about 20% of the cell division cycle, depending on the growth condition [26–28]. FtsK, FtsBLQ, FtsW, PBP3 and FtsN localize in an interdependent fashion. The FtsBLQ complex inhibits the PG synthase activities of PBP3, FtsW and PBP1B until it is outcompeted by the accumulation of FtsN that relieves this inhibition and initiates septum synthesis [29–32].

The maintenance of the PG sacculus is a dynamic process, which is achieved by the balanced activities of PG synthases and hydrolases that ensures a safe extension of the PG layer without defects that would cause cell lysis [33]. Beta-lactam antibiotics disturb this balance by inactivating the transpeptidase (TPase) activity of the penicillin binding proteins (PBP)s causing lysis of growing cells. We currently know more than twenty PG hydrolases that can cleave either in the glycan chains or peptides within the PG layer, but how their activities are regulated is poorly understood. This is particularly true for the PG endopeptidases [33].

The hydrolases are present in greater redundancy than the synthases [33,34]. The carboxypeptidases (CPase) PBP5, PBP6A and PBP6B remove the terminal D-Ala from nascent pentapeptides [35–38]. The endopeptidases (EPases) PBP4, PBP7, MepA, MepM, MepH, and MepS cleave the crosslinks in the PG [39–41]. The amidases AmiA-D hydrolyze the bond between Mur*N*Ac and L-alanine at position 1 of the stem peptide [25,42–44]. During cell division, as a complex, FtsEX recruits EnvC, which activates AmiA and AmiB that are needed for septum cleavage [43,45]. FtsE versions without ATPase activity still localize at midcell and recruit as FtsEX-EnvC complex but the latter is not able to activate the amidases [46]. Presumably an ATPase dependent conformational change in FtsEX induces a conformational change in EnvC, which is then able to relieve an autoinhibitory alpha-helix from the active site of AmiA and AmiB, activating them [47]. The third amidase AmiC is activated by the OM-anchored lipoprotein NlpD [25,48] and all three amidases AmiA-C are activated under certain stress conditions by the recently identified ActS [49,50]. Finally, the lytic transglycosylases Slt70 and MltA-G cleave the glycan strands to release 1,6-anhydroMur*N*Ac containing turnover products [51–53]. Although the large number of hydrolases may reflect the need to adapt to various environmental conditions [16,50], this still begs the question of how the cell controls their potentially dangerous activities to avoid cell lysis.

Recently, the OM bound lipoprotein NlpI was shown to interact with similar affinity with several endopeptidases, suggesting that NlpI acts as an interaction hub for endopeptidases to regulate their activities by competing protein-protein interactions. [54,55].

**Fig 1:**
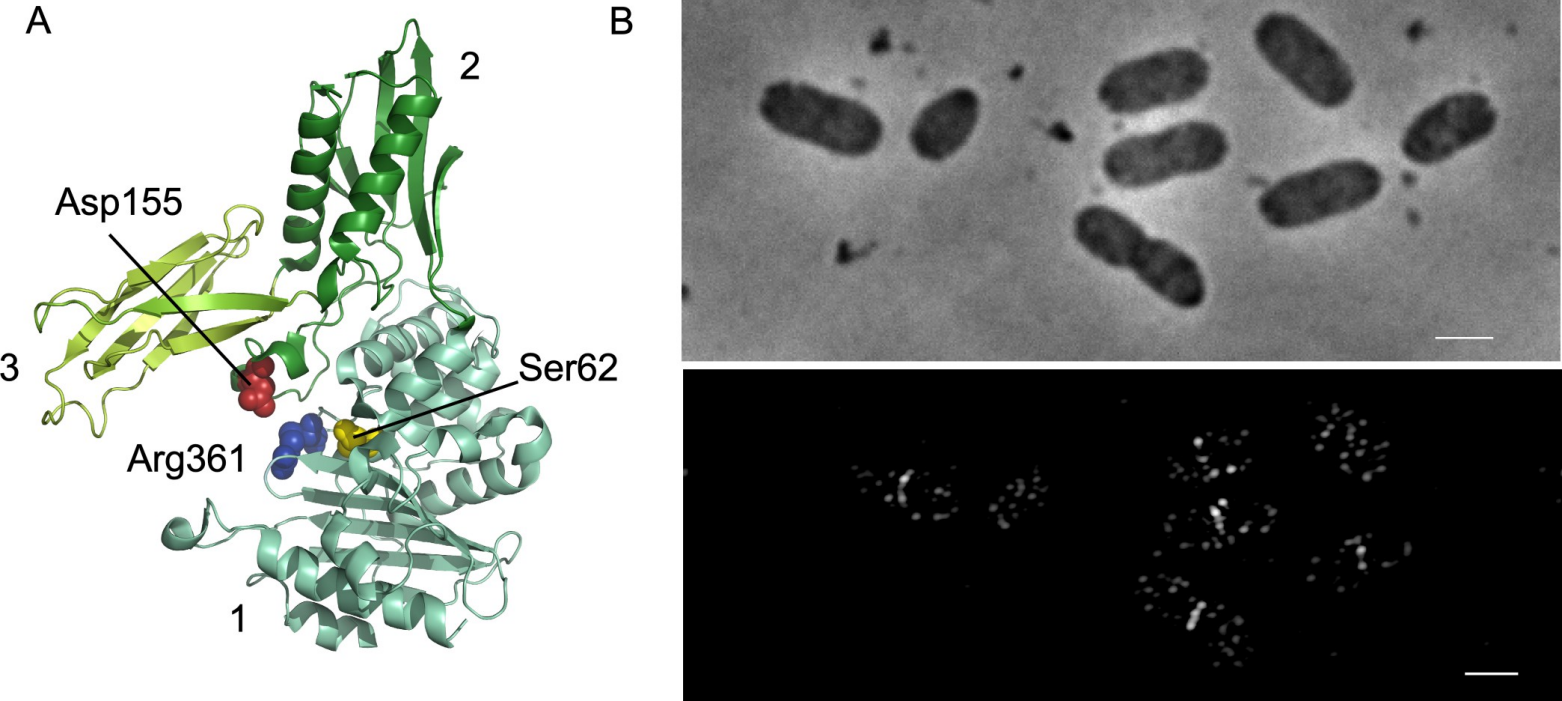
Structure of PBP4 and its localization at midcell and in the lateral wall during fast growth. A. Crystal structure of PBP4 in which the three domains (numbers 1–3) and some of the residues that are essential for its endopeptidase activity are indicated. B. MC4100 cells were grown exponentially in TY medium at 37°C to an OD600 of 0.3 and fixed and immunolabeled with BW25113Δ*dacB* pre-adsorbed anti-PBP4. Upper panel phase contrast image corresponding to the SIM fluorescence image in the lower panel. Scale bar equals 2 μm.

While investigating the specific function of various PG hydrolases, we found that PBP4 localizes specifically at midcell as part of the division machinery. PBP4 is a periplasmic endopeptidase [56] with a C-terminal amphipathic alpha-helix that associates with membranes [57] and has three domains that are assembled in an unusual way [58] (Fig 1A). A non-catalytical domain of unknown function (domain 2), which is inserted into the transpeptidase domain 1, and a third domain (domain 3), which is inserted into domain 2. Domain 3 is positioned above the active site (Serine 62, in domain 1), and might be involved in substrate binding or regulation [58,59] (Fig 1A). Site directed mutagenesis suggested that residues from domain 1 (R361) and 2 (D155) are important for the endopeptidase activity [60]. Deletion of *dacB*, which encodes PBP4, does not have morphological consequences but causes a decrease in the percentage of PG crosslinks and of PG-attached lipoprotein [61] and an increased sensitivity to bile salts [62]. A *dacB* deletion aggravates the morphological defects of a *dacA* (PBP5) deletion mutants [40], and the chaining phenotype of the ΔamiC strain [45]. PBP4 has also been reported to be involved in exopolysaccharide synthesis regulation [63]. Overproduction of PBP4 is toxic [64] presumably because the enhanced cleavage of peptide cross-links weakens the PG mesh and eventually causes lysis. There is litttle knowledge about the spatio-temporal regulation of PBP4 activity and its coordination with cell cycle events. Here we determined the cellular localization of PBP4 during the cell cycle of *E*. *coli*. The timing of localization at midcell and interactions of PBP4 support a role in remodeling of PG synthesized by the divisome.

## Results

### PBP4 localizes to the lateral wall and at midcell

**Fig 2:**
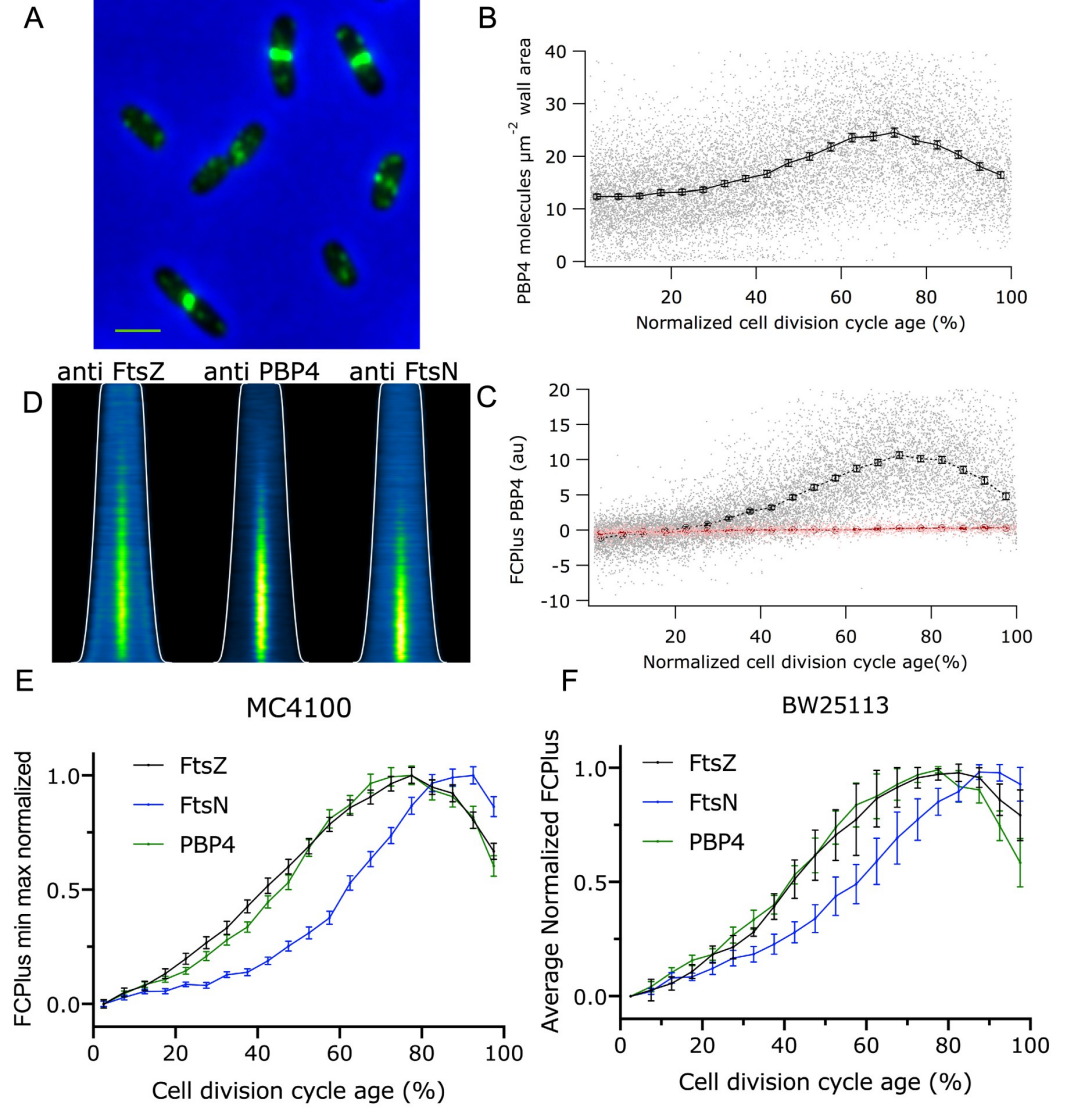
Cell division cycle timing of the localization of PBP4. **(**A) MC4100 cells were grown to steady state in minimal glucose medium at 28°C and immunolabeled with antibodies specific for PBP4. An overlay of a phase contrast and its corresponding fluorescence image is shown. Scale bar equals 2 μm. (B) The potential number of PBP4 molecules in the cell wall area (see results section), calculated from the volume of the cells is plotted as function of the cell division cycle age. The dots are the values for the individual cells and the markers are 5% age bins with 95% confidence. (C) The extra fluorescence at midcell compared to the rest of the cell (FCPlus) is plotted as function of the normalized cell division cycle age. The dots (grey for MC4100 and light pink for the BW25113 Δ*dacB* strain) are the values measured for the individual cells and the markers with bars (black for the wild-type MC4100, red for the BW25113 Δ*dacB*) are 5% age bins with 95% confidence. (D) Demographs of the localization of FtsZ, PBP4 and FtsN in the cells sorted according to cell length. The white line indicates the length of the cells. (E) Comparison of the timing of the localization at midcell of FtsZ (black), PBP4 (green) and FtsN (blue) during the cell division cycle age. The FCPlus values are min-max normalized to enable timescale comparison, despite differences in molecule number and antibody affinities. (F) BW25113 wild-type cells were grown to “steady state” in minimal glucose medium at 28°C and immunolabeled with antibodies specific for FtsZ, PBP4 and FtsN. The FCPlus of three independent biological experiments in 5% age bins was determined. The binned FCPlus values were min-max normalized. The average min-max normalized FCPlus values of the three experiments were subsequently plotted as function of the cell division age cycle.

To determine the localization of PBP4 in *E*. *coli* cells as a function of the bacterial cell cycle, we generated an antiserum against PBP4 and removed unspecific antibodies by adsorption to cells of a *dacB* deletion mutant that lacks PBP4. The purified antibodies were specific for PBP4 (S1 Fig) and used to immunolabel the wild-type strain MC4100 grown in rich (TY) medium at 37°C. PBP4 localized strongly at midcell and to a lesser extent in the lateral wall in wild-type cells (Figs 1 and 2).

### PBP4 localizes close to the peptidoglycan layer and the outer membrane

Although it was reported that PBP4 has a stretch of weakly amphipathic amino acids at its C-terminus [57], it was not known whether PBP4 freely diffuses in the periplasm or associates with the membrane or other proteins. It has been previously observed that chemically fixing freely diffusing periplasmic proteins can shock them into the cell poles [10,65]. The fact that we did not observe any polar localization of PBP4 after fixation (Figs 1B, 1C and S1), suggests that it is either associated with the membrane or other proteins. Next, we investigated whether PBP4 was accessible to anti-PBP4 in cells with an intact PG layer. For this, wild-type cells were grown in TY at 37°C and immunolabeled without the typically used Triton X-100-mediated permeabilization of the OM or without digesting the PG mesh with lysozyme. PBP4 was not accessible without permeabilization of the OM but was fully accessible in cells with an intact PG (S1D Fig). In contrast, cytoplasmic membrane bound proteins like FtsN and PBP3 were not accessible without degradation of the PG layer by lysozyme, whereas the outer membrane lipoprotein LpoB is accessible with Triton only (S1D Fig). This suggests that PBP4 resides close to the OM and the PG layer.

### PBP4 localizes as early divisome protein and its concentration in the envelope is not constant

Since PBP4 localizes at midcell it could be part of or associated with the divisome. The division machinery is assembled in two successive steps [26,66]. First, FtsZ and its membrane bound partners ZipA and FtsA in conjunction with other Z-ring associated proteins form the proto-ring that localizes at midcell [67]. Second, after approximately 20% of the cell division cycle the remaining proteins assemble subsequently at the division site. To obtain more details on PBP4 localization in relation to other cell division proteins, MC4100 cells were grown to steady state in minimal glucose medium at 28°C. The cells were immunolabeled with antibodies against PBP4, FtsZ (early localizing), or FtsN (late localizing). Interestingly, the concentration of PBP4 was not constant during the cell division cycle but increased simultaneously with its enhanced localization at midcell (Fig 2B and 2C). Using the ribosome profiling data from [68] on the average number of 133 PBP4 molecules per cell in a culture grown in MOPS minimal medium, we calculated from the total fluorescence per average cell in the culture and the amount of fluorescence at midcell (FCPlus) that about 100 PBP4 molecules are present at midcell during the process of septal PG synthesis. The strong localization bias of PBP4 at the midcell (Fig 2), suggests a dedicated function in the cell division process. To determine whether PBP4 belongs to the early or late localizing proteins of the divisome, we analyzed the timing of its midcell localization. To this end, we plotted the extra fluorescence (FCPlus) present at midcell in comparison to the amount of fluorescence in the rest of the cell (Fig 2D, 2E, and 2F). PBP4 localization coincided with that of FtsZ, indicating that PBP4 localizes relatively early in the cell cycle to midcell.

### Midcell localization of PBP4 depends on the FtsEX complex

**Fig 3:**
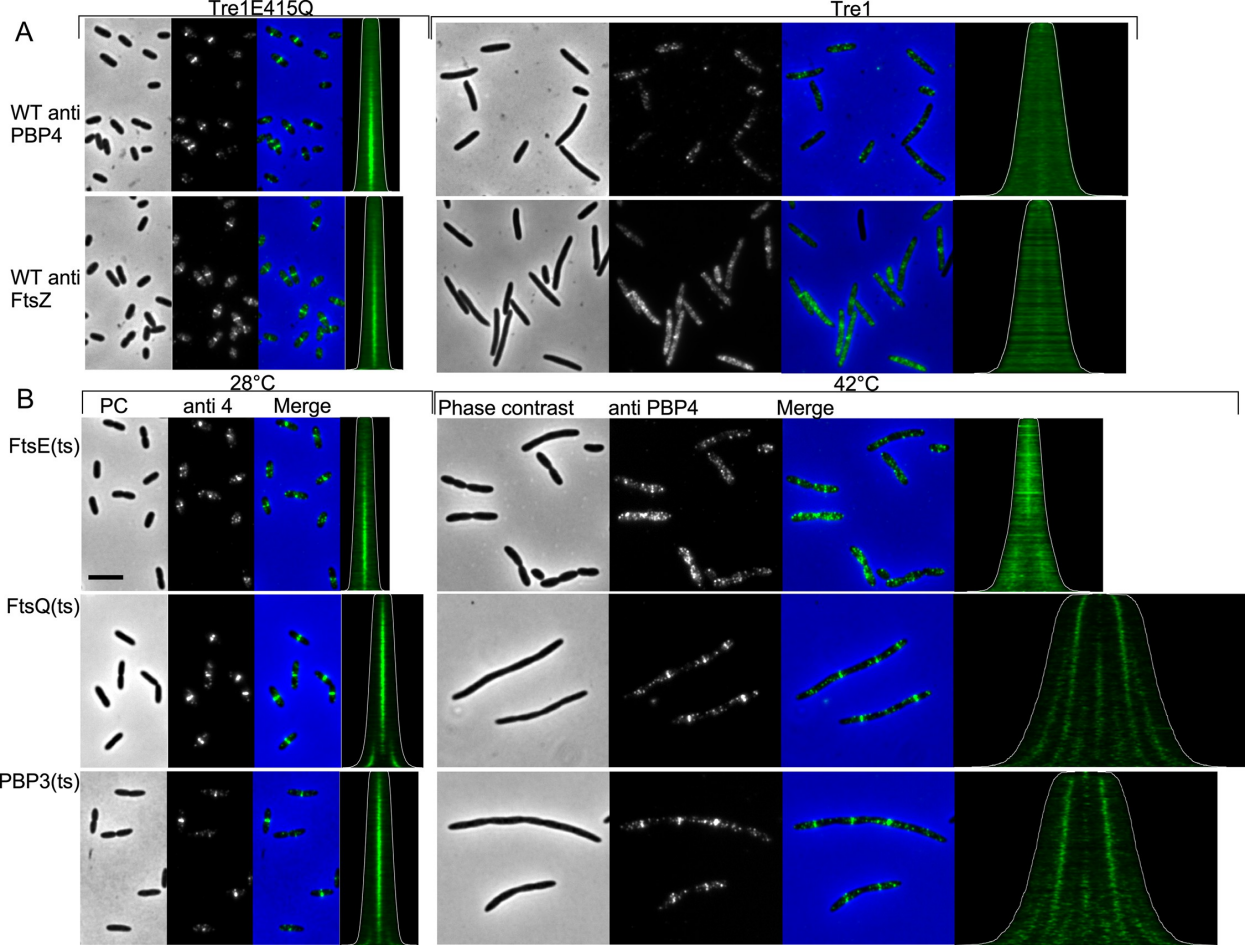
PBP4 localization is dependent on the presence of the proto-ring. A. MC4100 was transform with a plasmid that expressed either Tre1 that abolished the ability of FtsZ to polymerize by ADP-ribosylation of residue R174 or with the inactive variant Tre1(E415Q). Cells were grown in Gb1 medium at 28°C and expression of the inhibitor was induced for 2 mass doublings (MD) with 0.15% arabinose. Subsequently, the cells were fixed and immunolabeled with antibodies against FtsZ or PBP4. From left to right: the phase contrast (PC), corresponding fluorescence image of the PBP4 labeling and the merged images are shown. In the demographs the cells are sorted according to cell length (contours in white). The number of cells analyzed were 3694 and 3756 for Tre1E415Q and 1275 and 1621 for Tre1 for anti FtsZ and anti PBP4, respectively. B. Isogenic strains of MC4100 producing different temperature sensitive (ts) versions of cell division proteins were grown to steady state in Gb1 medium at 28°C and split in two parts. These were 1:4 diluted in prewarned medium and grown for two MD at either 28°C or 42°C. The cells were fixed, labeled with antibodies specific for PBP4 (anti4). From left to right: the phase contrast (PC), corresponding fluorescence image of the PBP4 labeling and the merged images at the permissive temperature, demograph of PBP4 fluorescence localization where cells are sorted according to their cell length (contours in white). This is followed by the same series from the non-permissive temperature samples. The number of cells analyzed were 2809 and 1576 for LMC515 FtsE(ts), 3833 and 1203 for LMC531 FtsQ (ts) and 3876 and 926 for LMC510 PBP3(ts), for the cells grown at 28°C and 42°C, respectively. The scale bar equals 5 μm. Within one antibody staining the brightness and contrast of the samples is identical and therefore directly comparable.

**Fig 4:**
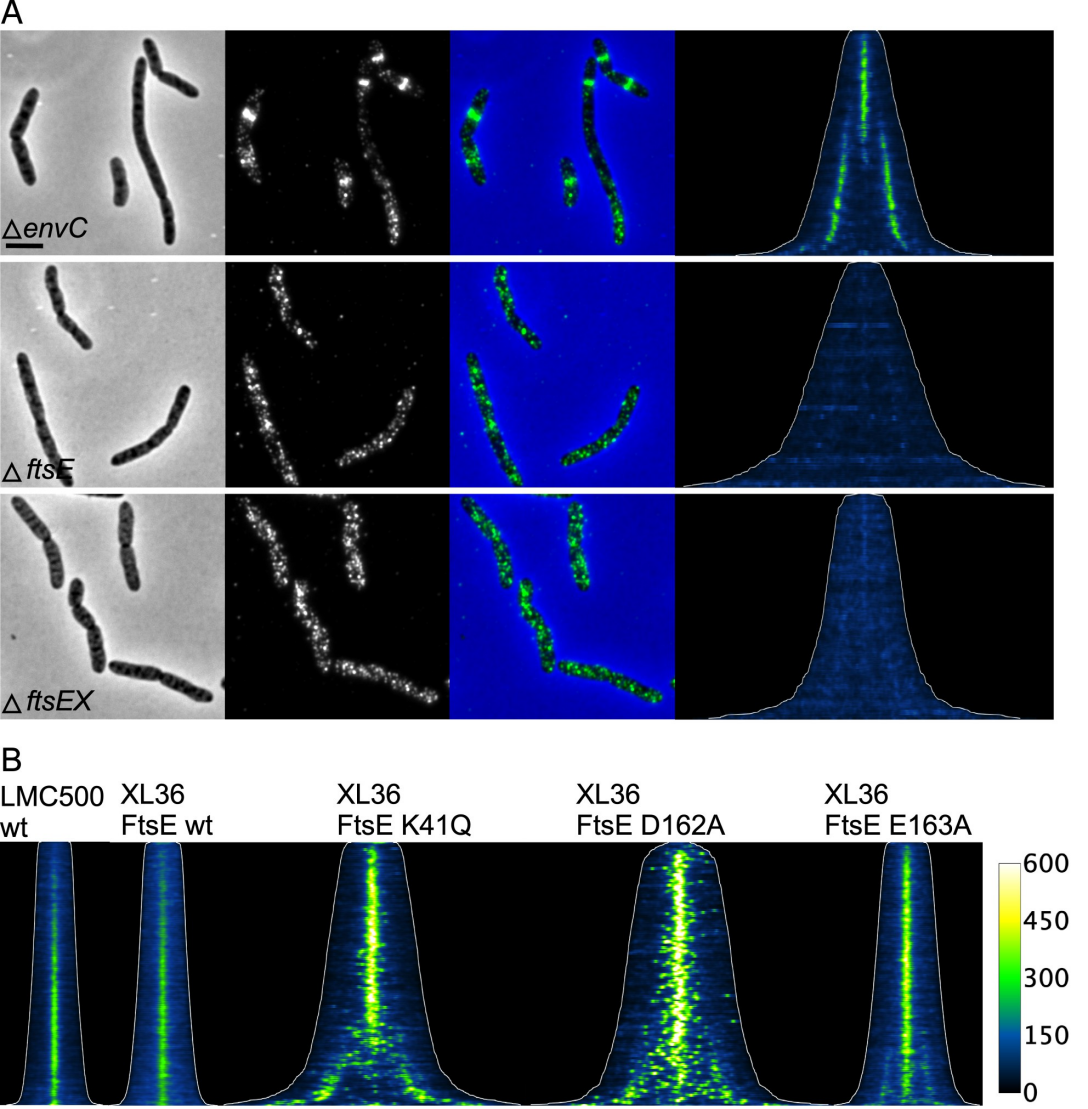
PBP4 is recruited by the septal cleavage complex FtsEX. (A) Cells of BW25113 expressing the proteins indicated were grown in LB at 37°C to OD_600_ = 0.3, fixed and labeled with specific antibodies against PBP4. From left to right, the phase contrast, corresponding fluorescence image of the PBP4 labeling, and the merged former two images, demograph of fluorescence with the cell lengths outlined (with the same contrast and brightness) for all demographs of PBP4 localization where cells are sorted according to their cell length are shown. The number of cells analyzed were 687 for Δ*envC*, 434 for Δ*ftsE*, and 346 for Δ*ftsEX*. The scale bar equals 5 μm. (B) Cells of LMC500 or XL36 (LMC500Δ*ftsE*::p*Trc99A*down *fts*X) expressing wild-type or active site versions of FtsE were immunolabeled with anti-PBP4 antibodies. Cells were grown in TY at 30°C and FtsE expression from plasmid was induced for 2 mass doubling with 30 μM IPTG, and cell were fixed and harvested at an OD_600_ of 0.3. The demographs of PBP4 fluorescence with the cell lengths outlined is shown for the indicated strains. Number of cells analyzed: LMC500 (2462), wild-type (2588), K41Q (728), D162A (554), and E163E (1052).

To investigate if PBP4 localization is dependent on the presence of the Z-ring or the later localizing division associated proteins we localized PBP4 in various division defective strains. First, the localization of PBP4 was assessed in a strain that expressed Tre1 that ADP-ribosylates 6 proteins including FtsZ at R174, which renders it unable to participate in protofilament formation [69,70]. The inactive E415Q variant of Tre1 was used as negative control. PBP4 was unable to localize in cells with insufficient FtsZ protofilaments to form division Z-rings (Fig 3A), whereas it localized as in WT cells in the presence of the TreE415Q variant, proving that a Z-ring is needed for PBP4 localization. Next, strains harboring temperature sensitive alleles of FtsE, FtsQ and PBP3 (FtsI) were grown in minimal glucose medium at 28°C to an OD_450_ of 0.2, diluted 1:4 in prewarmed medium of 28°C (permissive temperature) or 42°C (non-permissive temperature) and grown for two mass doublings before PBP4 immunolabelling. PBP4 localized at stalled division sites in filamentous cells with thermo-labile FtsQ or PBP3 indicating that these proteins are not needed to recruit PBP4 (Fig 3B). However, PBP4 localized very diffuse around potential division sites in the filamentous *ftsE*(ts) cells (Fig 3B). This suggests that a functional FtsE is important for PBP4 localization. The FtsEX/EnvC complex assists in the assembly of the divisome [71,72] and is essential to activate the amidase function of AmiA and AmiB to i) generate denuded glycan strands for the recruitment of the septal PG synthesis activator FtsN and ii) cleave PG for cell separation during constriction [46]. To investigate a possible link between PBP4 and the amidase activation complex we further looked for the localization requirement of PBP4 in a Δ*envC* strain, as EnvC bridges FtsEX with AmiA and AmiB, and is required for their activation [43,45]. EnvC was not necessary for PBP4 localization (Fig 4A), although PBP4 failed to localize in a Δ*ftsEX* strain (Fig 4A) and a newly constructed Δ*ftsE* single mutant strain. The *ftsE* and *fts*X genes reside in an operon and the expression of *fts*X requires the *fts*E gene [73]. We therefore constructed a Δ*ftsE* strain with a weakened p*Trc*99A promoter placed upstream of *fts*X to ensure sufficient expression of FtsX. The resulting strain, XL36 grew with a mild filamentous phenotype in rich medium that could be complemented by the expression of FtsE(wt) (S2 Fig). PBP4 did not localize at septal positions in the Δ*fts*E strain XL36 (Fig 4A) but localized normally when FtsE was expressed to complement this strain (Fig 4B). This suggests that the periplasmic part of FtsX enhances the septal localization of PBP4 and that this requires FtsE.

To determine whether the activity of FtsE was needed for the localization of PBP4, we expressed three inactive FtsE variants that had been shown to localize to midcell [73]. The K41Q and D162A versions of FtsE cannot bind ATP and FtsE(E163A) binds ATP but cannot hydrolyze it. PBP4 was able to localize at the division site in cells that expressed any of these inactive variants of FtsE as the only copy (Fig 4B), demonstrating that the activity of FtsE is not required for the localization of PBP4.

### Amidases affect the localization of PBP4

Interestingly, PBP4 localized strongly at division sites in a strain lacking the three amidases or lacking *amiA* and *B* but more diffuse in strains that lacked *amiC* or *nlpD* (S3 and S4 Figs). The absence of *amiAB* or *amiABC* also resulted in a 2- or 3-times enhanced expression of PBP4 compared to the parental wild-type strain. Furthermore, PBP4 localized as in wild-type in a strain lacking essential components of the Tol-Pal system, which is involved in the coordination of OM constriction and PG invagination during cell division (S4 Fig).

### PBP1A/LpoA contribute to midcell localization of PBP4

PBP4 was recently reported to interact with PBP1A, LpoA and the EPase adaptor protein NlpI [55]. To investigate whether PBP1A and/or LpoA proteins were needed for midcell localization of PBP4, strains deleted for *mrcA* (PBP1A), *lpoA*, *mrcB* (PBP1B) or *lpoB* were grown in rich medium at 37°C and immunolabeled with anti-PBP4. The amount of PBP4 that localized at midcell was identical for the parental wild-type strain BW25113, Δ*mrcB* and Δ*lpoB*, but reduced in the Δ*mrcA* and Δ*lpoA* strains (even after correction for the smaller diameter of these cells, [74], whereas the PBP4 concentration was similar for all strains (S5 Fig). This indicates that PBP1A/LpoA could assist in the localization of PBP4 at midcell.

### NlpI seems not to be involved in midcell localization of PBP4

As NlpI was reported to interact with PBP4 [55], we next tested if the interaction with the OM anchored NlpI affected the localization of PBP4. In the Δ*nlpI* cells the amount of PBP4 at midcell per μm average cell circumference was reduced by 42 ± 6.7% (n = 3) of the wild-type and the protein localized later at midcell than in the wild-type cells (S6 Fig). However, the PBP4 concentration in the Δ*nlpI* strain was also lower than in the wild-type cells (64.8 ± 17.6%, n = 3). This could indicate that the cells down-regulate PBP4 expression or that PBP4 becomes unstable in the Δ*nlpI* strain. Labeling of PBP4 in a Δ*nlpI* strain did not require lysozyme (S6 Fig), suggesting that NlpI is not needed to maintain PBP4 localized outside the PG layer, which could be facilitated by additional interactions of PBP4.

In conclusion, the midcell localization of PBP4 depends on the presence of the Z-ring and FtsEX, its distribution in the lateral wall and at midcell is affected by PBP1A, LpoA, and the amidases. Its concentration during the cells cycle is not constant and mutants have either less (Δ*nlpI*) or more Δ*amiABC)* PBP4 per cell than wild-type cells.

### PBP4 localizes at inactive divisomes

To further dissect whether the localization of PBP4 at the division site is dependent on the availability of its substrate or the presence of other proteins at the division site, cell division was inhibited through the specific inactivation of PBP3 by aztreonam [75]. Aztreonam treatment for 1–3 mass doublings stalls the division machinery at midcell resulting in filamentous growth. In the longer filaments new division machineries are formed and localize at future division sites [3]. In these filaments PBP4 still localized albeit somewhat weaker than in untreated cells at all possible division sites (S7 Fig), confirming that the activity of PBP3 was not required for the recruitment of PBP4. While PBP3 is inhibited, preseptal PG synthesis [76] still occurs at the potential division sites in these filamenting cells [16,36,77]. The localization of PBP4 mimics the localization of PBP1A in cells treated with aztreonam, which was previously shown to be recruited to preseptal PG synthesis sites by early cell division proteins [2,16].

### Activity of PBP4 is not needed for midcell localization

**Fig 5:**
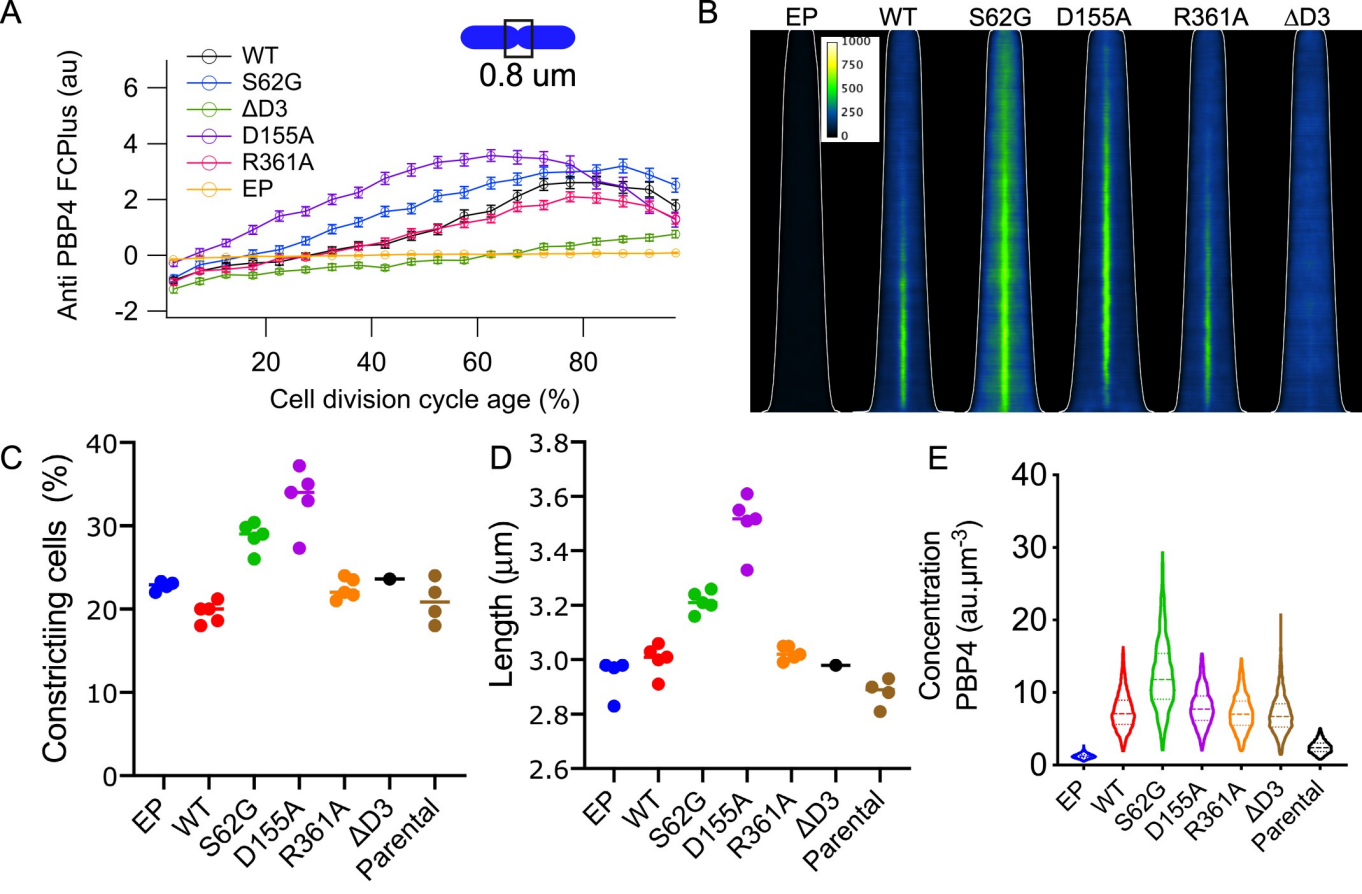
Localization of PBP4 depends on the presence of domain 3 but not on activity. Cells were grown in minimal glucose medium to steady state at 28°C, fixed and immunolabeled with antibodies against PBP4. (A) The extra fluorescence at midcell (FCPlus) in the Δ*dacB* strain transformed with the empty plasmid (EP, yellow), or plasmids expressing wild-type PBP4 (black), PBP4S63G (blue), PBP4D155A, (purple) PBP4R361A (red), PBP4ΔD3 (green) was determined and plotted as function of the cell division cycle age as in bins of 5% age classes with the error bar indicating the 95% confidence interval. (B) Demographs of the localization fluorescence pattern of the PBP4 variants shown in (A) with the cells sorted according to their cell length. The white line indicates the length of the cells. Intensity scaling is identical for all demographs. Number of cells analyzed for each immunolabeling was at least 2000 cells. Graphs with the percentage of constricting cells (C) and the average cell length in μm (D) for the various mutants expressed from plasmid without induction in the Δ*dacB* strain and of the parental strain BW24113 (n = 4). (E) The concentration of PBP4 in fluorescence units per μm^3^ in these cells for a representative experiment (out of the four repeats).

Substrate binding can be a key determinant in the localization of proteins [36,78,79]. To test whether PBP4 substrate binding is needed to localize at division sites we expressed the active site mutants PBP4(S62G) and (S62A) [59] that fail to bind β-lactams and are not able to hydrolyze the PG stem peptide (S8 and S9 Figs). A plasmid encoding the mutant protein was constitutively expressed in a Δ*dacB* background by basal activity of a weakened p*Trc99*A promoter. Interestingly, the wild-type and active site mutant when expressed from plasmid were both able to localize at midcell, although the S62G variant localized more diffusely (Fig 5). This indicates that although PBP4(S62G) cannot hydrolyze its substrate or bind β-lactams covalently (S8C and S9 Figs), it is either still able to interact with peptidoglycan or it localizes through protein interactions.

Since PBP4(S62G) could still interact with its PG substrate, we mutagenized the gene to express PBP4 versions in which residues D155 or R361 involved in the binding of the DAP residue in the stem peptide [60] were replaced by alanine. The genes encoding PBP4(D155A) or PBP4(R361A) were expressed again from same plasmid (p*Trc99*A) in a Δ*dacB* background, and both PBP4 versions localized at midcell (Fig 5). An immunoblot analysis showed that all mutants were expressed to similar levels and that PBP4(R361A) and PBP4(D155A) were able to bind the fluorescent β-lactam Bocilin-FL (S8 Fig). Therefore, all three active site protein variants are potentially able to interact with substrate as an inactive protein.

A PBP4 version lacking domain 3 (PBP4ΔD3) was still able to bind β-lactams (S8B and S8 Fig) but did not have DD-carboxypeptidase or DD-endopeptidase activity (S9 Fig). Analytical ultracentrifugation (S10A Fig) and circular dichroism analysis (S10B Fig) of isolated PBP4ΔD3 showed that the removal of domain 3 did not affect the dimerization of the rest of the protein. The truncated protein interacted with PG sacculi, albeit somewhat weaker than the native protein and perhaps non-specific, (S10C Fig). However, although the inactive PBP4ΔD3 was capable of binding substrate, it did not localize at midcell (Fig 5). Hence, we conclude that the midcell localization of PBP4 is driven by protein-protein interactions and/or by substrate interaction but is independent from its own activity.

Although the PBP4 variants were not induced, the leakiness of the plasmids was sufficient to produce PBP4 in 3 x excess to the wild-type strain, which clearly affected their morphological parameters (Fig 5C and 5D). The timing of the PBP4 localization of these mutants was also quite variable (Fig 5A). To verify whether the timing of the rest of the divisome had adapted itself to the timing of PBP4, cells grown to steady state in minimal medium at 28°C, were fixed, divided into three aliquots, and labeled with antibodies against FtsZ, PBP4 or FtsN. The timing of the arrival of these proteins at midcell was determined by analyzing fluorescence signals of several thousands of cells [80]. The initiation of localization at midcell was defined as the timepoint during the cell division cycle age where more fluorescence is detected at mid cell than in the rest of the cell (t_0_) and the time it would take to reach half of the maximum value of the fluorescence at midcell (t_1/2_) was determined by dividing the maximum FCPlus by two and noting the corresponding timepoint (see for a graphical explanation S11A or 6A Figs). We decided against using the maximum because this time point was more difficult to determine as the maximum FCPlus often reached a plateau before decreasing at the end of the cell cycle. Indeed, the variation in the timing of the PBP4 variants localization reflected differences in the timing of the assembly of the divisome (S11 Fig).

**Fig 6:**
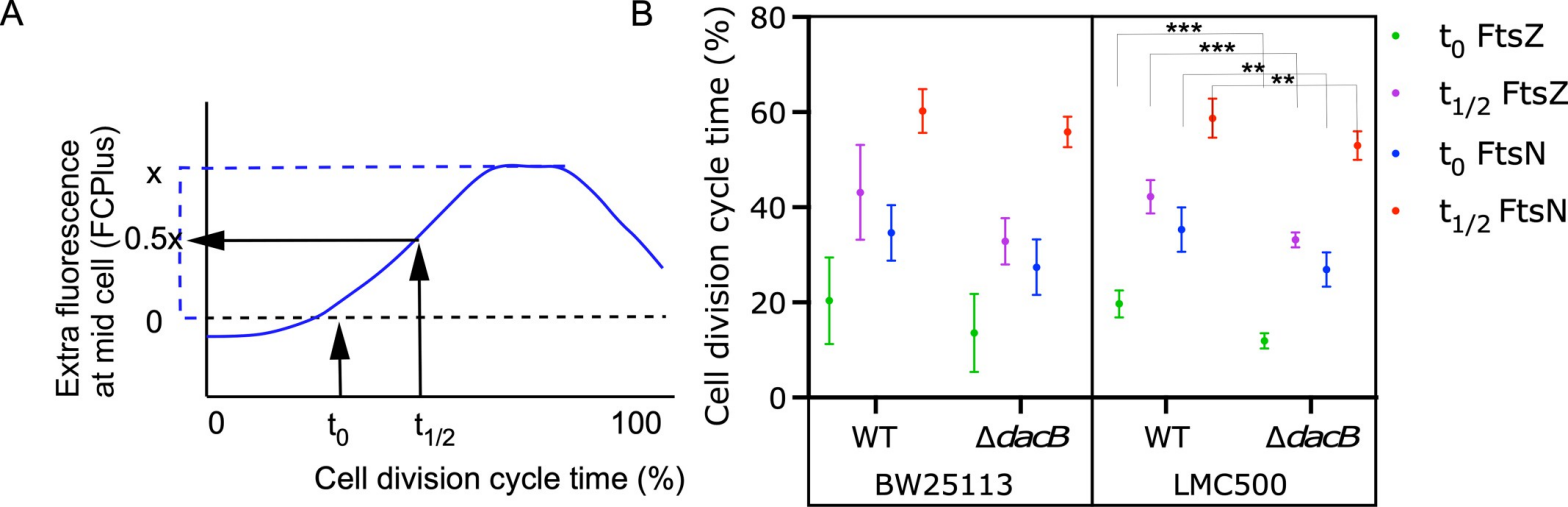
The absence of PBP4 advances divisome assembly. Cells were grown in minimal glucose medium to steady state at 28°C, fixed and immunolabeled against FtsZ and FtsN. (A) Graphical illustration of the meaning of t_0_ and t_1/2_. (E). Cell division cycle age timing of FtsZ and FtsN for the Δ*dacB* strain (10 and 6 replicates, respectively) and its parental BW25113 (7 and 6 replicates, respectively) and the Δ*dacB* strain (6 and 6 replicates, respectively) and its parental LMC500 (13 and 12 replicates, respectively). Using an unpaired T-test: P 0.0001 = ***, P 0.001 = **.

### PBP4 affects the timing of the divisome assembly

Because the PBP4 variants affected the morphology and the timing of the divisome assembly (Figs 5 and S11), we hypothesized a role for PBP4 in the divisome assembly. To verify this, we measured the timing of the divisome assembly in the absence of PBP4 in the ΔdacB strain and in its parental BW25113. A difference was observed between the two strains, which was not significant due to the large variation in the localization timing of FtsZ and FtsN in both strains (Fig 6B). We inferred that BW25113 is not able to reach a reproducible steady state, possibly due to a known frameshift in the *rph* gene, which leads to pyrimidine starvation and hence irregular DNA replication [81]. The uracil and thymine that we add as standard to the minimal medium for this strain is evidently unable to fully compensate the deficiency. To solve this possible issue, the *dacB* gene was deleted from the wild-type strain MC4100 and the cells were grown to steady state in minimal medium in the absence of an antibiotic. The results reproduced the difference between the Δ*dacB* and wild-type strains but now with a significance of P = 0.0001 (Fig 6B). No significant difference between the Δ*dacB* and its parental in other parameters that could account for a change in cell cycle such as mass doubling time, cell length, diameter or the percentage of constricting cells was detected (S1 Table). However, FtsZ started to localize (t_0_) 8% of the cell division cycle time earlier in the Δ*dacB* strain and reached 50% of its maximum intensity (t_1/2_) 9% earlier compared to the parental strain. FtsN localized 8.9% (t_0_) and 6.5% (t_1/2_) earlier (see for an explanation of t_0_ and t_1/2_ Fig 6A). These results show that the cells initiated divisome assembly significantly earlier in the absence of PBP4.

### The absence of PBP4 exacerbates filamentation of Δ*envC*

**Fig 7:**
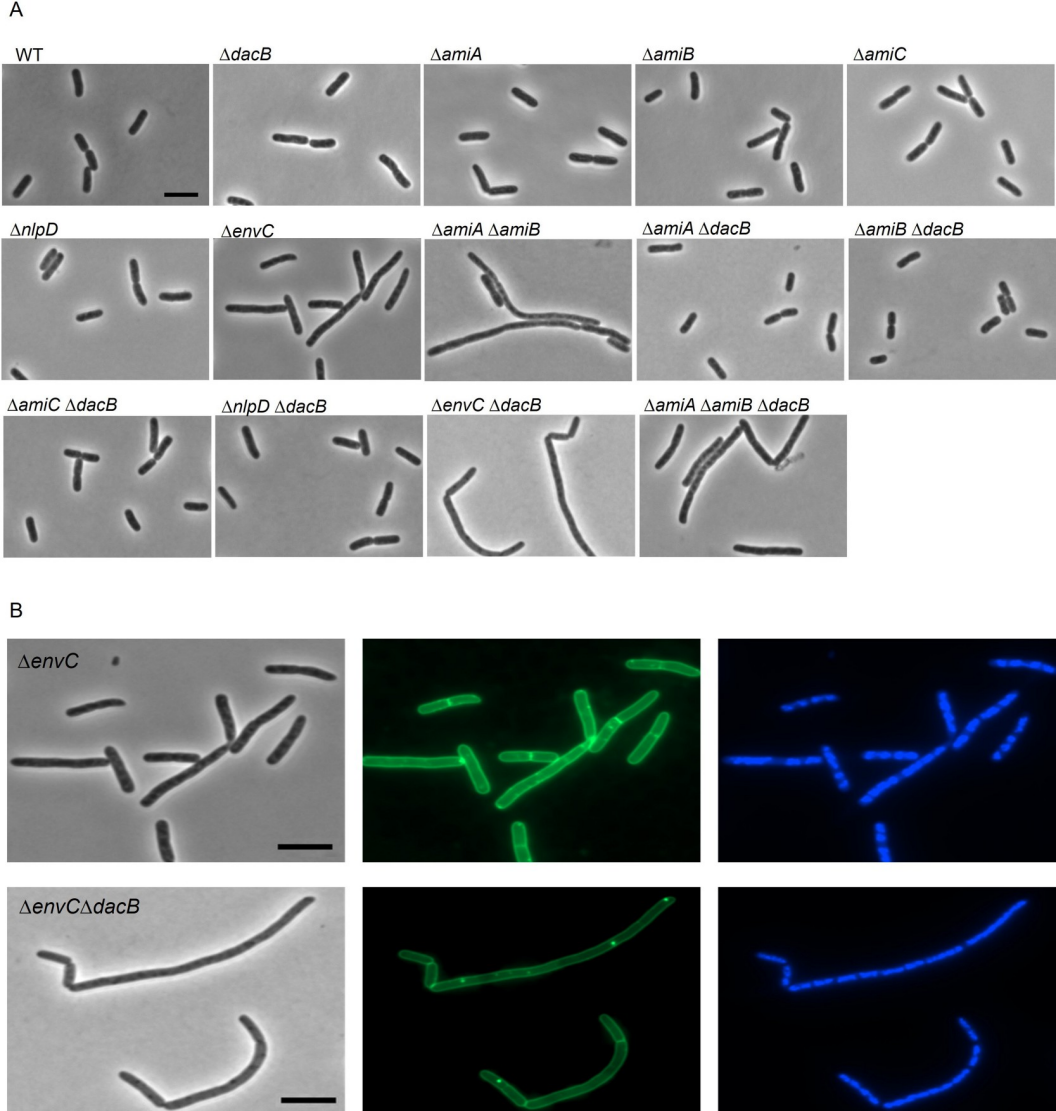
Morphology of deletetion strains lacking amidase or their regulators. (A) Phase contrast microscopy of WT, Δ*dacB*, Δ*nlpD*, Δ*envC*, Δ*amiA*, Δ*amiB*, Δ*amiC*, Δ*amiA*Δ*amiB*, Δ*nlpD*Δ*dacB*, Δ*envC*Δ*dacB*, Δ*amiA*Δ*dacB*, Δ*amiB*Δ*dacB*, Δ*amiC*Δ*dacB*, and Δ*amiA*Δ*amiB*Δ*dacB* cells in early-exponential phase growth (OD_600_ = 0.2) in LB medium at 37°C. (B) Microscopy of Δ*envC* and Δ*envC*Δ*dacB* cells. Cells were grown to early-exponential phase (OD_600_ = 0.2) in TY at 37°C, stained with the membrane dye FM1-43FX, fixed, and stained with DAPI. Cells were visualized by phase contrast microscopy and fluorescence microscopy. Scale bar equals 5 μm.

The early localization, the delay in divisome assembly, and the dependence on FtsEX for midcell localization in combination with the enhanced expression of PBP4 in a Δ*amiAB* strain, suggested a connection between the activity of PBP4 and the start of cell division. To test the hypothesis that PBP4 could help AmiAB to provide denuded glycan strands that would attract FtsN, the morphology of a series of deletion combinations was analyzed (S2 Table). Interestingly, Δ*envC* cell chains became twice as long when *dacB* was also deleted (Fig 7). In contrast, the morphology of mutants lacking single amidase genes or a double *amiAB* deletion mutant was not affected by the additional deletion of *dacB* (S2 Table). From these results we conclude that EnvC and PBP4 are both needed for the correct function of AmiA and/or AmiB.

### PBP4 domain 3 is needed for interaction with NlpI but not PBP1A/LpoA

**Fig 8:**
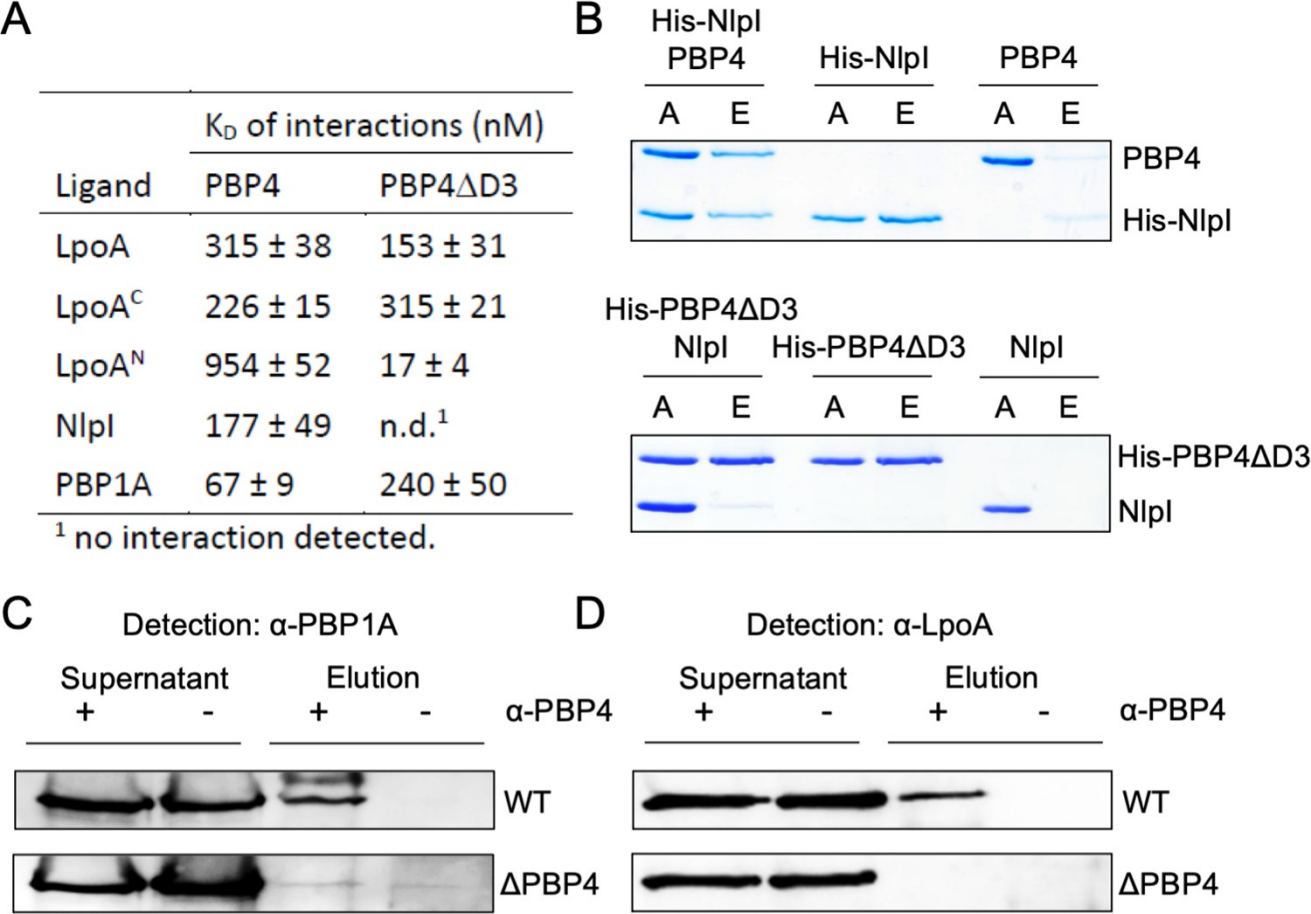
PBP4 interacts with PBP1A, LpoA and NlpI. (A) Summary of apparent K_D_ values of the interactions of PBP4 or a version lacking domain 3 (PBP4ΔD3) with LpoA, the C-terminal domain of LpoA (LpoA^C^), the N-terminal domain of LpoA (LpoA^N^), NlpI and PBP1A determined by microscale thermophoresis (MST). Corresponding binding curves are shown in S11 Fig (B) Pulldown experiment showing that domain 3 of PBP4 is required for interaction with NlpI. Oligo-histidine tagged NlpI (His-NlpI) pulled down PBP4 to Ni-NTA beads. His-tagged PBP3ΔD3 did not pull down untagged NlpI. A, applied sample; E, eluted sample. (C) *In vivo* cross-linking/co-immunoprecipitation showing interaction between PBP1A and PBP4. Growing cells of BW25113 (wt) or BW25113Δ*dacB* (ΔPBP4) were chemically cross-linked with DTSSP and cell extract was immunoprecipitated with purified anti-PBP4 antibody. Control samples did not receive anti-PBP4 antibody. The cross-linker was cleaved by reducing agent and proteins were separated by SDS-PAGE and transferred to a membrane, followed by detection of PBP1A with specific antibodies. PBP1A was detected in the elution of the sample from wild-type and not from ΔPBP4. (D) *In vivo* cross-linking/co-immunoprecipitation (as in panel C) showing that PBP4 and be cross-linked with LpoA in cells.

We next used microscale thermophoresis (MST) in which one protein is labelled with a fluorophore and titrated with an unlabeled partner protein to analyze the interactions between PBP4 and its known and putative interaction partners. Purified proteins were assayed for direct protein-protein interactions. We confirmed our previous result showing that wild-type PBP4 interacted with PBP1A, LpoA and NlpI [55], and we could now cross-link PBP1A to PBP4 in cells, followed by co-immunoprecipitation (Fig 8C). Interestingly, PBP4 lacking domain 3 (PBP4ΔD3) did not interact with NlpI (Fig 8).

We found that PBP4 and PBP4ΔD3 interacted with full length LpoA with apparent K_D_ values of 315 ± 38 nM and 153 ± 31 nM, respectively (Figs 8A and S12) suggesting that domains 1 or 2 of PBP4 are sufficient for the interaction with LpoA. MST assays of PBP4 or PBP4ΔD3 with LpoA’s C-terminal (residues N257-S678; LpoA^C^) or N-terminal (LpoA residues G28-T256; LpoA^N^) domains also yielded positive binding curves (Figs 8A and S12) [82–84]. LpoA^C^ and the full length LpoA version had comparable affinities for both PBP4 versions, suggesting that the C-terminal domain of LpoA is sufficient for interaction with PBP4 (Fig 8A). Interestingly, the removal of domain 3 from PBP4 resulted in an increased affinity of LpoA^N^ for PBP4 (app. K_D_ decreased by ~50 fold) (Figs 8A and S12). Hence, the interaction between the N-terminal domain of LpoA and PBP4 possibly occurs via a different mechanism and may be enhanced by a conformational rearrangement of domain 3 of PBP4. In conclusion PBP4 robustly interacts with PBP1A/LpoA independent of its domain 3.

### LpoA has a small effect on PBP4 activity

We found previously that NlpI does not affect the activity of PBP4 [55] but it was possible that PBP1A/LpoA have an effect. We first verified that NlpI or PBP4 did not alter the activity of PBP1A in the presence or absence of LpoA using an *in vitro* glycosyltransferase assay (S13 Fig). We then tested if PBP1A or LpoA affected the activity of PBP4 using different *in vitro* assays. LpoA decreased the DD-CPase activity of PBP4 by about 30% in a continuous assay with the soluble UDP-Mur*N*Ac pentapeptide substrate (S14B Fig) and its DD-EPase activity in an endpoint PG digestion assay (S14C Fig).

### Amidase activator EnvC interacts with PBP4 and NlpI

PBP4 localizes at midcell in cells lacking EnvC but not in cells lacking membrane anchored FtsEX. To begin investigating interactions of PBP4 with members of the amidase activation pathway, we first tested for the interactions with EnvC, a periplasmic protein with a coiled-coil and a LytM domain that activates AmiA and B upon interaction with FtsEX [85]. MST experiments showed that full-length EnvC interacted with full-length PBP4 (K_D_ = 307±45 nM), but substantially weaker with PBP4ΔD3 (K_D_ > 5900±2823 nM) (S14 Fig). The LytM domain of EnvC did not interact with PBP4 suggesting that the interaction between both proteins is mediated by domain 3 of PBP4 and the coiled-coil domain of EnvC. Hence domain 3 of PBP4 is needed for midcell localization (Fig 6) and is also used to bind to EnvC (Fig 8) and NlpI (S15 Fig). Yet the absence of none of these proteins alone is enough to abrogate the PBP4 midcell localization (Figs 3, 4 and S6).

During these studies, we also discovered an interaction between EnvC with NlpI, via the LytM domain of EnvC (S15 Fig). The different binding domains in EnvC for PBP4 and NlpI suggested that a trimeric complex could be formed. Indeed, a pulldown assay with oligo-histidine-tagged NlpI and untagged PBP4 and EnvC supported the formation of a trimeric complex. In summary, we show that PBP4 interacts with a component of the amidase-activation pathway, EnvC and with NlpI in a manner that can imply the formation of dynamic/different tripartite complexes that requires further investigation.

## Discussion

*E*. *coli* contains a large number of seemingly redundant PG hydrolases, which possibly fine tune peptidoglycan biogenesis and remodelling in response to environmental parameters [34]. Apart from their enzymatic activities, little is known about the function of most PG hydrolases. Here we report that the DD-carboxy/endopeptidase PBP4 localizes at midcell during septal PG synthesis in *E*. *coli* and is important for the timing of the assembly of the division machinery.

### PBP4 localizes at midcell during cell division in an FtsEX dependent manner

PBP4 localizes predominantly at midcell during cell division in an FtsEX dependent manner. Inactive PBP4 variants were able to localize at midcell indicating that substrate hydrolysis is not a requirement for localization. However, a version of PBP4 lacking the non-catalytic domain 3 (PBP4ΔD3), which is required for activity but not β-lactam binding, did not localize to midcell. Domain 3 is also required for the interaction with EnvC and NlpI, but not with PBP1A/LpoA. From these results we infer that the midcell localization of PBP4 requires multiple protein interactions, but not substrate hdyrolysis.

### PBP4 might be initially recruited by preseptal PG synthesis

**Fig 9:**
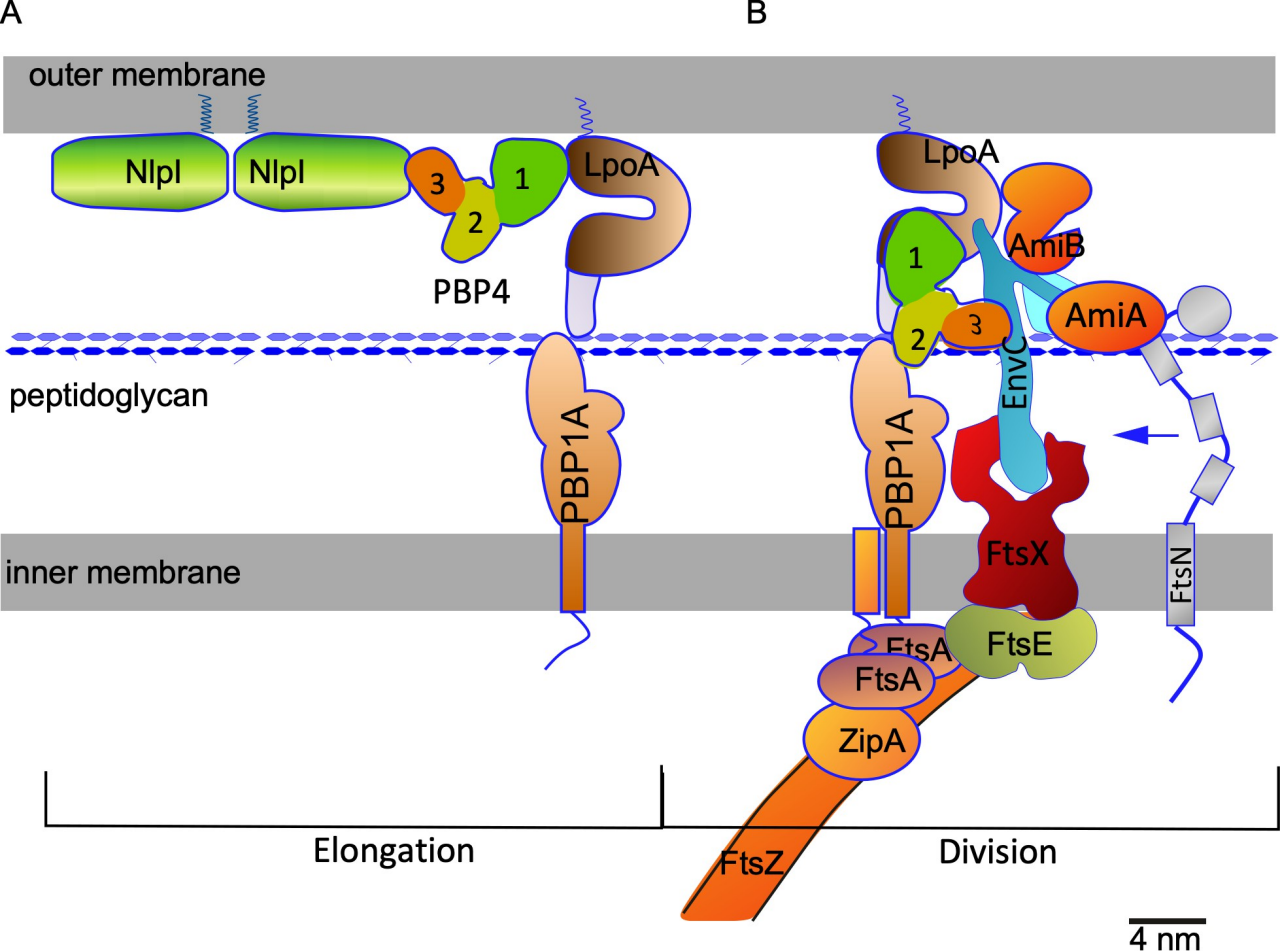
Model of the organization of PBP4 in the periplasm of *E*. *coli*. (A) During elongation PBP4 (or a fraction of the PBP4 molecules) is kept away from its substrate through its interaction with NlpI and possibly LpoA. (B) PBP1A and LpoA associate with ZipA and the Z-ring to assist in preseptal PG synthesis. Because of their presence, the absence of NlpI and the newly synthesized PG, PBP4 is attracted by PBP1A and LpoA at the septal synthesis site, where it interacts with the FtsEXEnvC complex. Preseptal PG synthesis might provide substrate for PBP4 and the activated AmiA and B to produce denuded strand that attract FtsN. All proteins apart from the FtsZ polymer have been drawn approximately according to their crystal structure shape and hydrated crystal structure sizes (scale bar equals 4 nm). The membranes are assumed to be 2 nm and the distance between the outer and the inner membrane 21 nm.

PBP4 interacts with NlpI, PBP1A and LpoA and these proteins can form a ternary complex ([55] and (Fig 8). PBP4ΔD3 interacted with PBP1A and LpoA but not with NlpI or EnvC, suggesting that NlpI and EnvC bind to domain 3. The interaction with PBP1A and/or LpoA may contribute to the recruitment of PBP4 at midcell, as the amount of PBP4 at midcell was slightly reduced in Δ*mrcA* and Δ*lpoA* strains. PBP1A and its partner LpoA are involved in preseptal PG synthesis [16,80]. Although somewhat weaker, PBP4 still localizes at putative division sites in cell filaments generated with aztreonam that have lost septal synthesis but not preseptal synthesis activity. Possibly, some PBP4 enriches at preseptal PG synthesis sites together with PBP1A/LpoA, which localize through the interaction of PBP1A with ZipA [16], and preseptal PG synthesis may provide initially the substrate for PBP4. The interaction between NlpI and PBP4 could function to sequester PBP4 in the lateral wall to prevent its interaction with potential substrate (Fig 9).

### Why does the loss of PBP4 cause premature divisome assembly?

The absence of PBP4 interferes with the timing of divisome assembly. In most bacteria studied thus far, the Z-ring and associated early division proteins localize first to prepare the future cell division site, perhaps by generating the border between the side wall and the future septum [16,76,86]. After a time-delay, the proteins that synthesize the bulk of the septal PG arrive and septation starts [26–28]. The absence of PBP4 causes a premature initiation of the assembly of the divisome for yet unknown reasons. The absence of PBP4 might enable NlpI to bind other EPases [54,55], coordinating their activity for remodelling the PG layer. However, the robust localization of PBP4 at midcell suggests a more direct role in modulating the timing of division initiation. We hypothesize that during cell elongation sufficient PBP4 molecules are retained by NlpI in the lateral wall above the PG layer to avoid promiscuous PBP4 activity (Fig 9), consistent with the reduced amount of PBP4 in cells lacking NlpI (S6 Fig and [55]). PBP4 might be initially recruited to midcell through interactions with PBP1A and LpoA, and the FtsEX-EnvC complex (Fig 9). The interaction of PBP4 with EnvC (S15 Fig) might ensure a concerted endopeptidase/amidase activity as EnvC activates AmiA and AmiB, which generate denuded glycan chains that attract FtsN, the trigger of septal PG synthesis. This is also suggested by the observation that a double Δ*dacB* Δ*amiC* mutant forms longer cell chains than a single Δ*amiC* strain [45] and by the observation that a Δ*envC* Δ*dacB* forms longer filaments than a Δ*envC* strain (Fig 7), whereas the deletion of *dacB* in an *amiA* and *amiB* double deletion strain has no exacerbating effect. As deleting *dacB* does not prevent the assembly of the divisome, it is plausible that other endopeptidases either contribute and/or partially take over the function of PBP4. Once septal peptidoglycan synthesis starts by the mature divisome, PBP4 remains at midcell as shown by the immunolocalization experiments (Fig 2). This could suggest that continued PBP4 activity is needed in cooperation with the amidases and other endopeptidase to create sufficient binding substrate for FtsN.

## Materials and methods

### Culturing conditions

*E*. *coli* K12 cells were grown to steady state [80] in glucose minimal medium containing 6.33 g of K_2_HPO_4_×3H_2_O, 2.95 g of KH_2_PO_4_, pH 7.0, 1.05 g of (NH_4_)_2_, 0.10 g of MgSO_4_×7 H_2_O, 0.28 mg of FeSO_4_×7 H_2_O, 7.1 mg of Ca(NO_3_)_2_×4 H_2_O, 4 mg of thiamine and 4 g of glucose per L. For strain MC4100 and its derivatives 50 μg lysine (Gb1) and for BW25114 and MG1655 derivatives 2 μg uracil, 20 μg thymidine, 50 μg arginine and glutamine (Gb4) per liter at 28°C or 37°C were added. Absorbance was measured at 450 nm with a 300-T-1 spectrophotometer (Gilford Instrument Laboratories Inc.). Alternatively, cells were grown in TY (5g yeast extract, 10 g bacto trypton and 85 mM NaCl, pH 7.0), or LB, which is TY with 170 mM NaCl and absorbance was measured at 600 nm.

### Strain construction

Strain XL35, the FtsE partial deletion strain, was constructed as follows: Primers priXL221 and priXL222 were used to amplify the upstream homologous sequences (*UHS*) from the *ftsE* start codon, and primers priXL223 and priXL224 were used to amplify the downstream homologous sequence after the FtsE 151^th^ amino acid (the potential promoter of *ftsX*), from the MC4100 genome. Primers priXL51 and priXL54 were used to amplify the kanamycin resistance cassette from plasmid pKD4 [87]. The amplified overlap PCR product (*UHS-FRT-KAN-FRT-DHS*) with primers priXL221 and priXL224 was used to construct the recombinant strain XL34 in which the first 150 amino acids encoding sequence of *ftsE* was deleted [87]. Subsequently, the kanamycin selection cassette was removed with plasmid pCP20 [87] yielding XL35.

Strain XL36, the *ftsE* clean deletion strain, was constructed similarly as XL35. Primers priXL221 and priXL222 were used to amplify the upstream homologous sequences from the *ftsE* start codon, and primers priXL225 and priXL226 were used to amplify the downstream homologous sequences after the *ftsE* stop codon, from the MC4100 genome. In order to restore the FtsX expression, a *pTrc99Adown* promoter [88] was amplified from plasmid pSAV057 [89] with primers priXL77 and priXL227 and a chloramphenicol selection cassette was amplified from plasmid pKD3 with primers priXL52 and priXL54. The amplified overlap PCR product (*UHS-FRT-CAM-FRT-DHS*) with primers priXL221 and priXL226 was used to construct the recombinant strain XL33 in which the entire *ftsE* coding sequence was deleted and FtsX was expressed from the chromosomally encoded p*Trc99Adown* promoter. Subsequently, the chloramphenicol selection cassette was removed with plasmid pCP20 yielding XL36 (Table 1). Bacterial strains construction for Δ*dacB* and amidases/regulators: *E*. *coli* BW25113 was the parental strain used for these experiments. *E*. *coli amiA*::*kan*, *amiB*::*kan*, *amiC*::*kan*, *envC*::*kan*, *nlpD*::*kan*, *dacB*::*kan* mutants were obtained from the KEIO library [90] and the mutations transduced into a clean parental strain. The strain *dacB*::*cam* was obtained from a previous study [55]. Double mutants were created by P1 transduction as described previously [91]. The chromosomal modification was confirmed by PCR when a Cam^R^ or Kan^R^ cassette was transduced.

**Table 1:** Strain and plasmids used in this study.

**Strain**	**Protein affected**	**Genotype**	**Source**
LMC500 (MC4100)	Wild-type	F^-^, *araD139*, *Δ(argF-lac)U169*, *deoC1*, *flbB5301*, *lysA1*, *ptsF25*, *rbsR*, *relA1*, *rpsL150*	[92]
BL21(DE3)	Overexpression strain	F^-^ *ompT*, *dcm lon hsdS hsdSB(rB-mB-)* λ(DE3)	Novagen
BW25113	Keio background strain	*F*^*-*^, *DE(araD-araB)567*, *lacZ4787(del)*::*rrnB-3*, *LAM-*, *rph-1*, *DE(rhaD-rhaB)568*, *hsdR514*	[87]
DH5α	Storage strain	F^-^ *endA1 glnV44 thi-1 recA1* ϕ80Δ*lacZ*ΔM15, λ^-^	Invitrogen
MC1061	Laboratory strain	Δ(*araA-leu*)7697 Δ(*lac*)X74 *galK*16 *galE1*5(GalS) λ^-^e14- *mcrA*0 *relA1 rpsL*150(str^R^) *spoT1 mcrB1 hsdR2*	[105]
LMC509	FtsZ (ts)	MC4100 *ftsZ*184	[92]
LMC511	FtsA (ts)	MC4100 *ftsA*10	[92]
LMC512	FtsA (ts)	MC4100 *ftsA*1882	[92]
LMC515	FtsE (ts)	MC4100 *ftsE*1181	[92]
LMC531	FtsQ (ts)	MC4100 *ftsQ*(ts)	[92]
LMC2487	FtsW (ts)	JLB17; F^-^ *thr*, *trp*, *his*, *thy*, *ara*, *lac*, *gal*, *xyl*, *mtl*, *rspL*, *tonA*, *ftsW* amino acid replacement G311D	[106]
LMC510	PBP3 (ts)	MC4100 *ftsI* 2158	[92]
BCB677	ΔPBP1A	BW25113Δ*mrcA*	[90]
BCB678	ΔPBP1B	BW25113Δ*mrcB*	[90]
BCB192	ΔLpoA	BW25113Δ*lpoA*	[90]
LMC2217	ΔLpoB	BW25113Δ*lpoA*	[90]
BCB655	ΔPBP4	BW25113Δ*dacB*::*kan*	[90]
BCB1433	ΔPBP4	XWL010 MC4100Δ*dacB*::*kan*	This work
BCB904	ΔNlpI	BW25113Δ*nlpI*::*frt pCP20* (NT10087)	This work
BCB197	ΔPal	BW25113Δ*pal*::*kan* (CAG70150)	[90]
BCB195	ΔTolA	BW25113Δ*tolA*::*kan* (CAG70204)	[90]
BCB742	ΔEnvC	BW25113Δ*envC*:: *frt* pPC20 (NT10086)	This work
BCB968	ΔAmiC	BW25113Δ*amiC*::*cam* (NT10090)	This work
BCB970	ΔAmiCΔNlpI	BW25113Δ*amiCΔnlpI*::*tet* (NT10266)	This work
BCB1153	ΔAmiAB	MC1061Δ*amiAB*:*cam*	[42]
BCB021	ΔAmiABC	MC1061Δ*amiABC*:*cam*, *kan*	[42]
BCB744	ΔFtsE	BW25113Δ*ftsE* kan^R^ CAG60397	This work
BCB746	ΔFtsEX	BW25113Δ*ftsE*X cat^R^ CAG60399	This work
BCB1081	ΔFtsE 1–150	XL34 LMC500Δ*fts*E 1–150::*kan*-*fts*X	This work
BCB849	ΔFtsE 1–150	XL35 LMC500Δ*fts*E 1–150::*fts*X	This work
BCB1082	ΔFtsE	XL33 LMC500Δ*fts*E::-cam-p*Trc99A*down *fts*X	This work
BCB850	ΔFtsE	XL36 LMC500Δ*fts*E::p*Trc99A*down *fts*X	This work
NT10022	ΔPBP4	BW25113Δ*dacB*::*cam*	[55]
MB01119	ΔAmiA	BW25113Δ*amiA*::*kan*	[90]
MB01120	ΔAmiB	BW25113Δ*amiB*::*kan*	[90]
MB01121	ΔAmiC	BW25113Δ*amiC*::*kan*	[90]
MB01051	ΔNlpD	BW25113Δ*nlpD*::*kan*	[90]
MB01122	ΔEnvC	BW25113Δ*envC*::*kan*	[90]
NT10274	ΔAmiAΔAmiB	BW25113Δ*amiA*::*kan*Δ*amiB*::*kan*	This work
MB01154	ΔAmiAΔPBP4	BW25113Δ*amiA*::*kan*Δ*dacB*::*cam*	This work
MB01155	ΔAmiBΔPBP4	BW25113Δ*amiB*::*kan*Δ*dacB*::*cam*	This work
MB01156	ΔAmiCΔPBP4	BW25113Δ*amiC*::*kan*Δ*dacB*::*cam*	This work
MB01157	ΔEnvCΔPBP4	BW25113Δ*envC*::*kan*Δ*dacB*::*cam*	This work
MB01158	ΔNlpDΔPBP4	BW25113Δ*nlpD*::*kan*Δ*dacB*::*cam*	This work
MB01159	ΔAmiAΔAmiBΔPBP4	BW25113Δ*amiA*Δ*amiB*::*kan*Δ*dacB*::*cam*	This work
**Plasmid**	**Name**	**Characteristics**	**Source**
pET21b	pET21b-PBP4(S62A)Δ1–60	Inactive PBP4 lacking residues 1–60, Amp^R^	[55]
pET21b	pET21b-His-PBP4(S62A)Δ1–60	For purification of His-PBP4 (active site mutant), Amp^R^	[55]
pET21b	pET21b-PBP4Δ1–60	Native PBP4 lacking residues 1–60, Amp^R^	[55]
pET28a	pET28a-His-LpoA(sol)	Soluble LpoA (LpoAΔ1–27) construct, N-terminal His-tag, Kan^R^	[82]
pET28a	pET28-His-LpoA^N^	Purification of His-LpoA^N^, Kan^R^	[82]
pET28a	pET28-His-LpoA^C^	Purification of His-LpoA^C^, Kan^R^	[82]
pTK1A-His	pTK1A-His	Full length His-PBP1A, Kan^R^	[82]
pET28a	pET28a-His-NlpI	Soluble NlpI construct, N-terminal His-tag, KanR	[4]
pBAD18	pBAD18-His-PBP4ΔD3	for purification of His-PBP4 lacking domain 3, Amp^R^	This work
pET28a	pET28a-His-EnvC	for purification of His-EnvC, Kan^R^	This work
pBAD18	Tre1	Arabinose inducible Tre1, Amp^R^	[69]
pBad18	Tre1 E415Q	TreE415Q inactive Tre1 variant, Amp^R^	[69]
pXL86	pTHV mCh-FtsE wt	p*Trc* 99A down expressing mCh-FtsE(wt) colE1 ori, Amp^R^	This work
pXL89	pTHV mCh-FtsE ts	p*Trc* 99A down expressing mCh-FtsE(ts) (P135S) colE1 ori, Amp^R^	This work
pXL110	pSAV mNG-NNN-FtsE	p*Trc* 99A down expressing mNG-FtsE, p15A origin, Cam^R^	This work
pXL133	pSAV-PBP4	p*Trc* 99A down expressing PBP4, p15A origin, Cam^R^	This work
pXL134	pSAV-PBP4 S62G	p*Trc* 99A down expressing PBP4 S62G, p15A origin, Cam^R^	This work
pXL135	pSAV-FtsE	p*Trc* 99A down expressing FtsE, p15A origin, Cam^R^	This work
pXL137	pSAV-FtsE K41Q	p*Trc* 99A down expressing FtsE K41Q, p15A origin, Cam^R^	This work
pXL138	pSAV-FtsE D162A	p*Trc* 99A down expressing FtsE D162A, p15A origin, Cam^R^	This work
pXL139	pSAV-FtsE E163A	p*Trc* 99A down expressing FtsE E163A, p15A origin, Cam^R^	This work
pXL140	pSAV-PBP4-ΔD3	p*Trc* 99A down expressing PBP4Δ173–247 (domain 3), p15A origin, Cam^R^	This work
pXL150	pSAV-PBP4-D155A	p*Trc* 99A down expressing PBP4 D155A, p15A origin, Cam^R^	This work
pXL151	pSAV-PBP4-R361A	p*Trc* 99A down expressing PBP4 R361A, p15A origin, Cam^R^	This work

Amp^R^, Kan^R^ and Cam^R^ are ampicillin, kananamycin and chloramphenicol resistance, respectively

### Plasmid construction

The *ftsE* gene was amplified from the chromosome of LMC500 [92] using the forward primer priXL187 and the reverse primer priXL188, and sequenced to characterize the mutation. The wild-type *ftsE* gene was cloned into pSAV057, p15A origin and Cam resistant [89] to produce the plasmid pXL135. *FtsE* was expressed under the control of the p*Tcr*99A down promoter. QuickChange site directed mutagenesis (Agilent technologies, Santa Clara, CA) and Gibson assembly [93] approaches were applied afterwards to construct the inactive *ftsE* mutant plasmids pXL137, pXL138 and pXL139, using the primers listed in Table 2.

**Table 2:** Primers used in this study.

Primer	Sequence 5’-3’	Purpose
priXL51	ACCATGGCTAATTCCCATGTCAG	GA_pkd3_F
priXL54	GTGTAGGCTGGAGCTGCTTC	PCR_pkd3_R
priXL187	CCGGCCGAATTCAACAACAACTCGATGATTCGCTTTGAACATGTCAGC	FtsE-EcoRI fw
priXL188	GGCCAAGCTTTTATTCATGGCCCACGCCTCC	FtsE-EcoRI rv
priXL221	CATGAAGCCGTTGGCTTAACC	ΔFtsE-FA-F
priXL222	TAGGAACTTCGAAGCAGCTCCAGCCTACACTGTTAATCCTCTCGGGCAAAAAG	ΔFtsE -FA-R
priXL223	GGATATTCATATGGACCATGGCTAATTCCCATGTCAGGCGGTGGTGAACAAGCCCGC	ΔFtsE- 1–150
priXL224	GGTGAGCATGCGATAGGAACG	ΔFtsE-1:150
priXL225	TGAGCGGATAACAATTTCACACAGGAAACAGACCATGAATAAGCGCGATGCAATC	ΔFtsE-clean
priXL226	CTTTTCAGATCCTGCAATGCG	ΔFtsE-clean
priXL230	ATTCCGGCGCAGGGCAAAGTACTCTCCTGAAGCTG	FtsE-K41Q-F
priXL231	GATCAGCTTCAGGAGAGTACTTTGCCCTGCGCCGGA	FtsE-K41Q-R
priXL232	CGGTACTGCTAGCGGCTGAACCGACTGGTAACCTG	FtsE-D162A-F
priXL233	AGGTTACCAGTCGGTTCAGCCGCTAGCAGTACCGC	FtsE-D162A-R
priXL234	GTACTGCTAGCGGACGCTCCGACTGGTAACCT	FtsE-E163A-F
priXL235	AGGTTACCAGTCGGAGCGTCCGCTAGCAGTACCGC	FtsE-E163A-R
priXL236	AGATGGCGCTGCCTGCCGGCACCCAGAAAG	PBP4-S62G-F
priXL244	GCGCGAATTCatgCGATTTTCCAGATTTATCA	EcoRI-PBP4-F
priXL245	GCGCAAGCTTctaATTGTTCTGATAAATATC	HindIII-PBP4-R
priXL250	GTTGACCGCAACGGTGGCGGAAGCGGGTCTGTGCAGGATGGAGCCAGCTATG	PBP4ΔD3-F
priXL251	TCCATCCTGCACAGACCCGCTTCCGCCACCGTTGCGGTCAACTATGGCGGC	PBPΔD3-R
priXL266	CCGGCTGGCCATGGAATGCCATGACACAATGCTTTAGCGCTC	PBP4-D155A-F
priXL267	GGAGCGCTAAAGCATTGTGTCATGGCATTCCATGGCCAGCCGG	PBP4-D155A-R
priXL268	GCCGATGGTTCAGGGCTTTCGGCGCATAACCTGATTGCCCCCGCC	PBP4-R361A-F
priXL269	GGCGGGGGCAATCAGGTTATGCGCCGAAAGCCCTGAACCATCGGC	PBP4-R361A-R
prXW5-F	TAGTATGACGGCTCGATTCCAGGTTGTTAGCGCGAGATTgtgtaggctggagctgcttc	LMC500Δ*dacB*
priXW6-R	AATCTGAAGCCCCGGCCATGTGCCGGGGTTTCTTTTTGAatgggaattagccatggtcc	LMC500Δ*dacB*
Env-F	GGAATTCcatatg GATGAGCGTGACCAACTC	pET28a-His-EnvC
Env-R	CCCaagcttttaTCTTCCCAACCACGGC	pET28a-His-EnvC

To construct the PBP4 expression plasmids, the wild-type *dacB* gene was firstly amplified from the *E*. *coli MG1655* genome using primers priXL244 and priXL245, and subsequently cloned into plasmid pSAV057 and pSAV057-dsba^ss^-mCherry-PBP5 (pNM010 [38]) with *EcoRI* and *HindIII*, to generate the PBP4 plasmids without and with mCherry fusion. Similarly, QuickChange site directed mutagenesis and Gibson assembly approaches were applied afterwards to construct the inactive *dacB* mutant plasmids from these two wild-type plasmids, using the primers listed in Table 2. Tre1 and Tre1E415Q were expressed from the arabinose inducible promoter of pBAD18 as described [69].

### Immunolabeling

After reaching steady state for minimal glucose grown cells or the desired OD for rich medium grown cells, the cells were fixed for 15 min by addition of a mixture of formaldehyde (f. c. 2.8%) and glutaraldehyde (f. c. 0.04%) to the cultures in the shaking water bath and immunolabeled as described [94] with Rabbit polyclonal antibodies against PBP4, NlpI [55] preabsorbed against *ΔdacB* or *ΔnlpI*, strains, respectively, or against FtsZ or FtsN [26]. As secondary antibody, donkey anti-rabbit conjugated to Cy3 or to Alexa488 (Jackson Immunochemistry, USA) diluted 1:300 in blocking buffer (0.5% (wt/vol) blocking reagents (Boehringer, Mannheim, Germany) in PBS) was used, and the samples were incubated for 30 minutes at 37°C. For immunolocalization, cells were immobilized on 1% agarose in PBS slabs coated object glasses as described [70] and photographed with an Orca Flash 4.0 (Hamamatsu, Japan) CCD camera mounted on an Olympus BX-60 (Japan) fluorescence microscope through a 100x/*N*.*A*. 1.35 oil objective. Images were taken using the program ImageJ with MicroManager (https://www.micro-manager.org).

SIM images were obtained with a Nikon Ti Eclipse microscope (Japan) and captured using a Hamamatsu Orca-Flash 4.0 LT camera. Phase contrast images were acquired with a Plan APO 100x/1.45 Ph3 oil objective. SIM images were obtained with a SR APO TIRF 100x/1.49 oil objective, using 3D-SIM illumination with a 561nm laser, and were reconstructed with Nikon-SIM software using the values 0.20–0.25–0.20 for the parameters Illumination Modulation Contrast (IMC), High Resolution Noise suppression (HNS) and Out of focus Blur Suppression (OBS), respectively.

### Image analysis

Phase contrast and fluorescence images were combined into hyperstacks using ImageJ (http://imagej.nih.gov/ij/) and these were linked to the project file of Coli-Inspector running in combination with the plugin ObjectJ (https://sils.fnwi.uva.nl/bcb/objectj/). The images were scaled to 15.28 pixel per μm. The fluorescence background has been subtracted using the modal values from the fluorescence images before analysis. Slight misalignment of fluorescence with respect to the cell contours as found in phase contrast was corrected using Fast-Fourier techniques as described [80]. Data analysis was performed as described [80]. In brief, midcell was defined as the central part of the cell comprising 0.8 μm of the axis. From either cell part, midcell and remaining cell, the volume, the integrated fluorescence, and thus the concentration of fluorophores was calculated. The difference of the two concentrations is multiplied with the volume of midcell. It yields FCPlus (surplus of fluorescence) at midcell. For age calculation, all cell lengths are sorted in ascending order. Then the equation

age=ln(1‐0.5*rank/(nCells‐1))/ln(0.5)

is used, where *rank* is a cell’s index in the sorted array, *nCells* is the total number of cells, and *age* is the cell’s age expressed in the range 0.. 1. To analyze the FCPlus or the total concentration of fluorescence as a function of cell division cycle age, the data points of the individual cells were binned in 5% age classes and plotted with the 95% confidence interval indicated as error bars. To determine the timing of the divisome assembly, the moment that more fluorescent material was present at midcell (FCPlus) than in the rest of the cell was taken as timepoint zero of arrival (t_0_) and then the maximum FCPLus value (usually a small plateau) was divided by half and the corresponding timepoint was noted as the half maximum fluorescent intensity time value (t_1/2_).

### Δ*dacB* and amidases/regulators fluorescence imaging

To image membrane and DNA compartmentalisation of the cells, FM1-43FX dye (Invitrogen) and DAPI (Stratech Scientific) were used, respectively. Overnight cultures were adjusted to initial OD_600_ = 0.01 in LB. The cells were incubated at 37°C until OD_600_ = ~0.2. A volume of 500 μl of the culture was stained with 5 μg ml^-1^ of FM1-43FX at room temperature for 10 min. The cells were adjusted to 33 mM sodium phosphate pH 7.4 and fixed by addition of 2.4% formaldehyde and 0.04% glutaraldehyde. Fixed cells were inoculated on agarose pads, which were prepared with 1.5% agarose in PBS and set in Gene Frames (Thermo Scientific). Cells were imaged using a Zeiss AxioObserver equipped with a Plan-Apochromat 100x/Oil Ph3 objective and illumination from HXP 120V for phase contrast images. For FM1-43FX images Zeiss filter set 38 (Ex: 470/40 nm, beamsplitter 495 nm, Em: 525/50 nm) was used. DAPI images were captured using the Zeiss filter set 96 (Ex: 390/40 nm, beamsplitter 420, Em: 450/40 nm). For phenotype analysis in S2 Table we used MicrobeJ plugin for Fiji 600 [95].

### Proteins

Proteins were purified from overexpression strains according to published procedures: NlpI, PBP4, PBP4(A62) and PBP4ΔD3 [55]. LpoA, LpoA^N^ and LpoA^C^ [82]; PBP1A [4]. EnvC was purified from BL21(DE3) pET28a-His-EnvC. The overexpression plasmid pET28a-His-EnvC was constructed by amplifying a soluble EnvC version without the N-terminal, 34 amino acid long, signal peptide into the pET28a (+) vector using the restriction enzymes *Nde*I and *Hind*III. To purify recombinant EnvC, cells which were grown in 3 L LB with 50 μg/ml kanamycin to OD578 0.5–0.6, at 37°C. Protein overproduction was induced with 1 mM IPTG for 3 h at 30°C before cells were harvested by centrifugation and lysed by sonication before ultracentrifugation at 14,000 *× ɡ*, 1 h, 4°C. The cell lysate was centrifuged and the supernatant was applied to a 5 ml HisTrap HP column (GE healthcare). The column was washed with 4 column volumes of 25 mM Tris/HCl, 300 mM NaCl, 20 mM imidazole, pH 7.5 at a flow rate of 2 ml/min. The oligohistidine-tagged EnvC was eluted using 25 mM Tris/HCl, 300 mM NaCl, 400 mM imidazole, 10% glycerol, pH 7.5. Protein purity and yield was analysed by SDS-PAGE and fractions of interest were pooled. The oligohistidine tag was removed by incubating the samples with 1 unit/ml of restriction grade thrombin (Novagen), overnight at 4°C, during dialyses against 25 mM Tris/HCl, 200 mM NaCl, pH 8.0. The sample was concentrated to ~ 5 ml using Vivaspin concentrator spin columns (Sartorius) at 4,500 *× ɡ* at 4°C. The sample was then applied to a HiLoad 16/600 Superdex 200 column (GE healthcare) at 1 ml/min pre-equilibrated with 25 mM HEPES/NaOH, 300 mM NaCl, 10%, glycerol, pH 7.5. Fractions were analysed by SDS-PAGE and those with the highest protein purity and yield were pooled and stored at -80°C.

### Ni^2+^-NTA pulldown assays

Nickel-Nitrilotriacetic acid (Ni-NTA) beads (100μl) were pre-equilibrated with dH_2_O and binding buffer (10 mM HEPES/NaOH, 10 mM MgCl_2_, 150 mM NaCl, 0.05% Triton X-100, pH 7.5) by centrifugation at 4000 *× g*, 4 min at 4°C, and then incubated with proteins of interest. Equimolar amounts (1–2 μM) of His_6_-tagged and untagged proteins were incubated alone (control) and in combination, for 10 min at 4°C in a 200 μl reaction volume, containing 10 mM binding buffer. An aliquot of this mixture is taken as an ‘applied’ sample. The protein samples were then added to pre-equilibrated Ni-NTA beads and incubated overnight on a spinning plate at 4°C with 1.3 ml of binding buffer.

Beads were centrifuged at 4000 *× g*, 4 min, 4°C and washed a further 4–6 times with 1 ml of washing buffer (binding buffer with 30 mM Imidazole) before re-suspending in 250 μl washing buffer and transferring beads to Proteus spin columns (Generon), centrifuging as described above. Bound proteins were eluted by the addition of 50 μl of SDS-PAGE loading buffer and boiling at 100°C for 5 min. Spin columns were centrifuged a final time at 1500 *× g*, 5 min, RT, to collect ‘eluted’ protein before separation by SDS-PAGE. Eluted samples were run alongside applied samples for comparison.

### Microscale thermophoresis

Microscale thermophoresis (MST) is an immobilisation-free method which allows the detection of biomolecular interactions in solution. This technique is based on the specific directed movement of a protein along a heat gradient (thermophoresis), an observation first reported by Carl Ludwig in 1856 [96]. The thermophoresis of a protein changes upon ligand binding due to one or more changes to size, charge and/or hydration shell. In MST, a localized heat gradient is initiated by an IR-laser (wavelength 1470 nm). A protein of interest is fluorescently labelled and the change in its thermophoretic mobility, in the presence of an unlabelled ligand, is measured and expressed as a change in the normalised fluorescence (FNorm).

Proteins of interest (10–20 μM) were fluorescently labelled with an amine reactive dye (NT-647- N-hydroxysuccinimide (NHS)), cysteine reactive dye (NT-647 maleimide) or a histidine reactive dye (NT-647-Tris-NTA), according to manufacturer’s instructions (Nanotemper). Fluorescently labelled proteins were diluted to an appropriate concentration and a fixed concentration was titrated against a two-fold serial dilution of unlabelled ligand, across 16 samples, in MST running buffer (25 mM HEPES/NaOH, 150 mM NaCl, 0.05% Triton X-100, pH 7.5).

MST measurements were carried out as described in [97], using standard or premium capillaries on a Monolith NT.115 MST machine (Nanotemper). Binding curves and Kinetic parameters were plotted and estimated using manufacturer provided software (NT Analysis 1.5.41 and MO. Affinity Analysis (x64)). Capillary scans were carried out prior to all measurements to check for consistent fluorescence counts, confirming that any subsequent change in fluorescence was due to ligand binding and not due to inaccurate pipetting or adsorption and dilution effects.

### SDS-denaturation (SD) test

In instances where capillary scans showed a ligand concentration dependant change in raw fluorescence, we investigated whether this was a property of the binding interaction by carrying out an SD-test.

Samples (10 μL) with the highest and lowest concentration of unlabelled ligand were centrifuged 10 000 × *g*, 5 min, RT and mixed 1:1 volume ratio with SD-test buffer (40 mM DTT, 4% SDS). Mixtures were boiled at 100°C for 10 min, to abolish ligand binding, before being spun down and subjected to another capillary scan. If the fluorescence between samples that contained the highest and lowest concentration of ligand, after SDS treatment, were now back to ± 10% of each other, the initial observations were a property of ligand binding, and a binding curve was plotted from the raw fluorescence data. If the ligand concentration dependent change in fluorescence was still observed, then this indicated that the fluorescently labelled protein was aggregated, and assay and buffer conditions were optimised accordingly.

### *In vivo* cross-linking / co-immunoprecipitation assays

Method is described in, and adapted from [98]. An overnight culture of *E*. *coli* BW25113 cells and an appropriate mutant strain was used to inoculate 150 ml of Lennox LB (Fisher Scientific) and was cultivated to an OD_578_ of 0.5–0.6 at 37°C before harvesting by centrifugation (4500 × g, 4°C, 25 min). Cells were resuspended in 6 ml of CL buffer 1 (50 mM NaH_2_PO_4_, 20% sucrose, pH 7.4). The amine reactive cross-linker, DTSSP (3,3’-dithiobis (sulfosuccinimidylpropionate) (ThermoFisher), was freshly dissolved (20 mg/ml in dH_2_O) and added to the isolated cell suspension and incubated at 4°C with agitation for 1 h. Cross-linked cells were then harvested by centrifugation (4500 × g, 4°C, 25 min) and resuspended in 6 ml CL buffer 2 (100 mM Tris/HCl, 10 mM MgCl_2_, 1 M NaCl, pH 7.5). DNase, protease inhibitor cocktail and phenylmethylsulfonly fluoride were added prior to sonication at low levels before ultracentrifugation of the lysate (140,000 × g, 4°C, 1 h). The membrane pellet was resuspended in 2.5 ml of CL buffer 3 (25 mM Tris/HCl, 10 mM MgCl_2_, 1 M NaCl, 1% Triton X-100, 20% glycerol, pH 7.5) and the solubilised membrane extracted o/n with stirring at 4°C.

Samples were ultracentrifuged (140,000 *× g*, 4°C, 1 h) to remove debris, before removing 2 × 1.2 ml of each supernatant to be subsequently diluted with 0.6 ml of CL buffer 4 (75 mM Tris/HCl, 10 mM MgCl_2_, 1 M NaCl, pH 7.5). One sample was incubated with an optimised concentration of specific antibody with the other used as a negative control. Both samples were incubated at 4°C with agitation for 5 h. For the isolation of antibodies, and thus cross-linked interaction partners, 100 μl of protein G-coupled agarose bead resin (Roche) were washed (2 × CL buffer 4, 2 × CL wash buffer [2:1 CL buffer 3 and CL buffer 4]) and added to each sample, and incubated o/n at 4°C with agitation.

Samples were centrifuged and the supernatant retained before washing the beads 10 × 1 ml with CL wash buffer. After the final wash, beads were resuspended in 250 μl CL wash buffer and transferred to 2 ml spin dry columns and centrifuged to isolate the beads. These were then resuspended in 50 μl of fresh SDS-loading buffer and boiled to elute bound proteins, and reverse cross-linkage, and were collected by centrifugation (10,000 *× g*, RT, 5 min). Supernatant and elution samples were resolved by SDS-PAGE and transferred to a nitrocellulose membrane by Western blotting to detect for specific interaction partners using purified antibodies. The secondary antibody used here is Trueblot Anti-Rabbit IgG-HRP specific for native antibodies.

### Peptidoglycan digestion assays

To test for hydrolase activity on PG, 10 μl of sacculi isolated from strains of interest (usually *E*. *coli* strains MC1061, BW25113 or D456) were incubated with 1–10 μM of respective enzymes, 37°C for between 1 h and overnight, as indicated for respective proteins. The standard reaction conditions were 10 mM HEPES/NaOH, 10 mM MgCl_2_, 150 mM NaCl, 0.05% Triton X-100, pH 7.5, in 100 μl reaction volume. Following incubation, samples were boiled at 100°C, 10 min, to terminate reactions before digesting remaining PG overnight at 37°C, with 1 μM cellosyl. The samples were centrifuged at 10,000 × *g* for 5 min, RT, to obtain digested muropeptide products in the supernatant.

To test for activity against soluble muropeptides; first, 100 μl of intact sacculi were incubated overnight at 37°C with 1 μM cellosyl and cellosyl digestion buffer (20 mM NaPO_4_, pH 4.8). Next, samples were boiled at 100°C, 10 min, before centrifugation at 10,000 × *g* for 5 min, RT, to obtain the soluble muropeptides in the supernatant [99]. Ten μl of the supernatant was then used as muropeptide substrate for incubating with enzymes of interest, for the appropriate incubation time, at 37°C. Reactions were then terminated by boiling at 100°C for 10 min.

After digestion of respective substrates, products were reduced with NaBH_4_, adjusted to pH 4–5 and separated for analysis by reversed-phase HPLC as described below in HPLC analysis.

### Reduction of muropeptides with sodium borohydride

PG digestion samples were transferred to 2 ml vials following centrifugation. Muropeptides were reduced in a 1:1 volume ratio of sodium borohydride buffer (0.5 M sodium borate, pH 9.0) and a small spatula of sodium borohydride pellets, centrifuging at 3000 × *g*, 30 min, RT as described in [99]. The samples were adjusted to pH 4–5 using HPLC grade phosphoric acid before separation and analysis by reversed-phase HPLC.

### Reversed-phase HPLC analysis of muropeptides

Following the protocol of [99], reduced muropeptides were separated for analysis on reversed-phase HPLC systems with a Prontosil 120-3-C18-AQ 3 μm reversed-phase HPLC column (Bischoff). A linear gradient of solvent A (50 mM sodium phosphate, pH 4.31 supplemented with 0.2% NaN_3_) to 100% solvent B (75 mM sodium phosphate, pH 4.95, 15% methanol) over 90 or 180 min was used to separate muropeptides at 52°C. Unlabelled muropeptides were detected at UV absorbance 205 nm. In assays where ^14^C-radiolabelled muropeptides were used, detection was achieved by flowing scintillation cocktail along with standard buffers to give a radioisotope scintillation count (radioactivity CPM). Muropeptide profiles were recorded and analysed using Laura V4.2.11.129 (LabLogic Systems Ltd.).

### Spectrophotometric D-alanine release assay (DD-carboxypeptidase assay)

This protocol was adapted from [60]. The carboxypeptidase (CPase) activity of PG hydrolases results in the release of the terminal D-Ala residue from the pentapeptide stem of PG precursors. Using UDP-Mur*N*Ac pentapeptide as a substrate, and in this case PBP4, it was possible to spectrophotometrically measure the release of D-Ala.

Each reaction sample consisted of 200 μl of CPase buffer (50 mM HEPES/NaOH, 10 mM MgCl_2_, pH 7.6), 3 units of D-amino acid oxidase (Sigma), 6 units of horseradish peroxidase (HRP) (Sigma), Amplex Red (Sigma), and an optimised concentration of protein.

All constituents of the reaction were added and mixed directly in a quartz cuvette (Hellma, 10 mm light path, 15 mm centre), before the addition, and brief mixing by pipette, of purified UDP-Mur*N*Ac pentapeptide (BACWAN, Warwick University) to begin the reaction. The released D-Ala residues from the CPase activity of PBP4 are oxidatively deaminated by the action of D-amino acid oxidase to produce pyruvate and hydrogen peroxide (H_2_O_2_). The released H_2_O_2_ is reduced to H_2_O by HRP using Amplex Red as an electron donor. Oxidised Amplex Red produces resorufin, which has an intense pink colour and the production of which was measured spectrophotometrically using a Cary 100 Bio UV-visible spectrophotometer (wavelength 555 nm). The change in absorption over 10 min was measured and analysed using the complementing software.

### Circular dichroism

Proteins were dialysed overnight against 10 mM NaPO_4_, pH 7.5 and concentrated/diluted to 0.4 mg/ml. CD measurements were taken using a Jasco J-810 spectropolarimeter (Jasco, Tokyo, Japan) using a wavelength range of 180–250 nm. The average of 10 runs was taken for each protein with a buffer control subtraction. For a direct comparison, correcting for the differing amino acid sequences, the collected data were converted to molecular CD and plotted against wavelength (nm). The resulting CD spectra are compared in S4 Fig and show that PBP4 lacking domain 3 is folded, consisting of both α-helices (~190, 208 and 222 nm) and β-sheets (~210 nm).

### Analytical ultracentrifugation

Purified PBP4 and PBP4ΔD3 were dialyzed over night against 25 mM HEPES/NaOH, 150 mM NaCl, pH 7.5, in preparation for AUC experiments. AUC sedimentation velocity (SV) experiments were carried out in a Beckman Coulter (Palo Alto, CA, USA) ProteomeLab XL-I analytical ultracentrifuge using absorbance at 280 nm and interference detection. The AUC runs were executed at the rotation speed of 45,000 rpm and the temperature of 20°C using an 8-hole AnTi50 rotor and double-sector aluminium-Epon centerpieces. The sample volume was 400 μl and the sample concentrations ranged between approximately 0.25 and 1.4 mg/ml. The partial specific volumes ($\bar{v}$) of the proteins were calculated from their amino acid sequence, using the program SEDNTERP [100]. Sedimentation velocity boundaries were analyzed using the size-distribution c(s) model implemented in the program SEDFIT http://www.analyticalultracentrifugation.com [101]. The experimental values of the sedimentation coefficient were converted to the standard conditions (s20,w), which is the value of sedimentation coefficient in water at 20°C. The size-distribution peaks were integrated to obtain the weight-averaged values for sedimentation coefficient and molecular mass.

The atomic coordinates from the published high-resolution structure of [58] (pdb accession code 2EX2) were used to calculate the sedimentation coefficient values for the monomeric and dimeric forms of PBP4 /PBP4ΔD3 using the program SoMo [102]. PBP4 /PBP4ΔD3 crystallographic dimer was built using program PyMol (https://pymol.org/2/).

### PG binding assay

To assay for interactions of respective proteins with purified PG, we used a PG binding protocol as described in [103]. Briefly, ~100 μg purified PG was pelleted by centrifugation (10,000 × *g*, 10 min, 4°C) and resuspended in binding buffer (10 mM Tris/Maleate, 10 mM MgCl_2_, 50 mM NaCl, pH 6.8). PG was incubated with 10 μg of desired protein before incubating on ice for 30 min and centrifuging at 10,000 × *g*, 10 min, 4°C. An aliquot of the supernatant (S) was taken for SDS-PAGE. The pelleted PG was washed with 200 μl binding buffer by centrifugation as before and the wash supernatant was taken as sample W for SDS-PAGE. The pelleted PG was then resuspended in 100 μl of 2% SDS and incubated for 1 h on a stirring plate, 4°C. Mixtures were centrifuged a final time at 10,000 × *g*, 10 min, 4°C, with the supernatant, now containing any initially bound protein, taken as the pellet sample (P). A negative control sample with no PG was assayed in parallel to determine that binding of protein was specific. Samples, along with corresponding controls, were analysed by SDS-PAGE and visualised by Coomassie staining. Presence of protein band in pellet sample (P) indicated binding to PG, whilst presence in supernatant sample (S) indicated no binding.

### Bocillin binding assay

A fluorescent-bocillin binding assay was used to determine whether a PBP was purified with a correctly folded active site. Bocillin FL is a commercially available dye-β-lactam conjugate, synthesised from penicillin V and BODIPY (Molecular Probes, In., Eugene, Oreg.). Ten μg of purified PBP was incubated for 10 min at 37°C, with 20 ng of Bocillin FL in a 50 μl volume with 10 mM HEPES/NaOH, 10 mM MgCl_2_, pH 7.5. A negative control sample was pre-incubated with 1 mM ampicillin for 30 min, 37°C, to block the PBP active site, prior to incubation with Bocillin FL. After incubation, samples were boiled at 100°C for 10 min with 40 μl of SDS-PAGE loading buffer and resolved by SDS-PAGE. Fluorescent signal was observed using Typhoon Fluorescence-imager (Excitation; 488 nm, emission; 520 nm, BP20 PMT 400–600 V), followed by visualisation of gels by Coomassie staining. Proteins incubated with ampicillin should have no detectable binding of Bocillin FL. For whole cells, a culture was grown in 5 mL TY and induced with 10 μM IPTG for 3 mass doublings to an OD_600_ of 0.5 and the cells were pelleted and washed once with PBS (8 g NaCl, 1.8 g Na_2_HPO_4_, 0.24 g KH_2_PO_4_ per liter, adjusted to pH 7.4) at 4°C and resuspended in 100 μl PBS with 5μg/ml Bocilin FL and incubated for 20 min at RT. To remove the excess Bocillin FL, the samples were pelleted and resuspended in 100 μl PBS with cOmplete Mini Protease Inhibitor Cocktail (Sigma) and 2 U DNAse I (NEB). Then sonicated on ice for 10 sec and boiled 15’ at 99°C after adding 25 μL of SDS-PAGE 5x loading buffer and resolved by SDS-PAGE. Fluorescent signal was observed using a home-made imager for Midori green, followed by visualisation of gels by Coomassie staining.

### *In vitro* PG synthesis assay with radiolabelled lipid II

The assay was published by Bertsche *et al*., [104]. Lipid II (1.2 nmol, 11,000 dpm) was vacuum dried and dissolved in 5 μl of methanol. The reaction was performed in a total volume of 50 μl in 25 mM Tris/HCl, 10 mM MgCl_2_, 100 mM NaCl, 0.05% Triton X-100, pH 7.5 (buffer B), with 0.5 μM PBP1A, 1 μM LpoA, 1 μM PBP4(S62A), 2 μM NlpI. The reaction was performed for 1 h at 30°C. The pH of the samples was adjusted to 4.8 prior to boiling for 5 min followed by a digestion with 50 g/ml cellosyl for 1 h at 37°C and boiling for 5 min.

## Supporting information

S1 Fig Pre-adsorption of antibodies against Δ*dacB* BW25113 strain results in PBP4 specific antibodies and PBP4 localizes close to the PG layer. (A) Demograph with cells sorted according to length of PBP4 fluorescence of the BW25113Δ*dacB* (PBP4) strain, BW245113 (wt) strain before purification of the antibody and the BW25113Δ*dacB* strain and BW245113 strain after purification of the antibody. (B) Absolute fluorescence average profiles of cells from the BW25113 Δ*dacB* strain and the BW245113 strain before purification of the antibody and from the BW245113 strain after purification of the antibody (C) Phase contrast and corresponding fluorescence image of anti PBP4 immunolabeled BW25113Δ*dacB* (left) and wild-type cells using the supernatant of BW25113Δ*dacB* pre-adsorbed antibodies. The scale bar equals 5 μm. (D) BW25113 wild-type cells were grown in TY at 37°C and harvested in the exponential phase at an OD600 of 0.3 and fixed. Samples were divided into two aliquots and cells were immunolabeled with antibodies against PBP3, FtsN, LpoB and PBP4. The first aliquot was immunolabeled after permeabilizing the cell membrane with Triton X-100 (right: phase contrast and fluorescence images) and the second after permeabilizing cells with Triton X-100 and lysozyme that cleaves the glycan strands of the PG layer (left: phase contrast and fluorescence images). The scale bar equals 5 μm. PBP4 and LpoB, but not PBP3 or FtsN, are accessible without degrading the peptidoglycan layer.
(TIF)

S2 Fig NG-FtsE effectively localizes and recruits PBP4. Strains were grown in LB at 37°C to an OD of 0.3 in the presence of 30 μM IPTG to induce expression of mNG-FtsE then fixed and immunolabeled with antibodies specific for PBP4. (A) LMC500 (1108 cells analyzed). (B) XL36 (LMC500::p*Trc*99down*ftsX*, Δ*ftsE*) with pXL110 mNG-FtsE(wt) (1189 cells analyzed). From left to right, phase contrast image, anti-PBP4 immunolabeling, mNG-FtsE, and the corresponding demograph of the diameter and the demograph of fluorescence of cells sorted according to length. Scale bar equals 5 μm.
(TIF)

S3 Fig PBP4 localizes less pronounced in Δ*amiC* and is expressed 2 and 3-fold higher in a Δ*amiAB and* Δ*amiABC* strain, respectively. Isogenic strains of the wild-type strain MC1061 were grown in LB at 37°C to an OD600 of 0.3, fixed and labeled with specific antibodies against PBP4. (A) Demographs of the fluorescence distribution in cell sorted according to their length. The numbers above the graph indicate the adjustment of the brightness and the contrast, which had to be different for the double and triple-*ami* deletion strain as they produced more PBP4 than the other strains. (B) peak normalized fluorescence along the cell length of all cells in the demograph. (C) Images showing examples of the localization of PBP4 (green) overlayed with the phase contrast of the corresponding cells. The number of cells analyzed were 3311 for MC1061 (*wt*), 1691 for Δ*amiC*, 1865 for Δ*nlpI AmiC*, 926 for Δ*amiAB* for 484 for Δ*amiABC*. The scale bar equals 2 μm.
(TIF)

S4 Fig PBP4 localization is not dependent on the presence of TolA, Pal, or NlpD. Isogenic strains of the wild-type strain BW25113 were grown in LB at 37°C to an OD600 of 0.3, fixed and labeled with specific antibodies against PBP4. From left to right, the phase contrast, corresponding fluorescence image of the PBP4 labeling, and the merged former two images, demograph of diameters (Dia) and demograph of fluorescence (Fluor) PBP4 localization where cells are sorted according to their cell length are shown. The number of cells analyzed were 1053 for BW25113 (*wt*), 918 for Δ*tolA*, 1314 for Δ*pal*, and 1600 for Δ*nlpD*. The scale bar equals 5 μm.
(TIF)

S5 Fig PBP1A and LpoA contribute to PBP4 midcell localization. Cells were grown exponentially to an OD of 0.3 in TY at 37°C, then fixed and immunolabeled with antibodies against PBP4. Because fluorescence imaging by microscopes is usually not directly comparable between different experiments, all results in each experiment were normalized to the parental strain BW25113. (A) PBP4 fluorescence at midcell per μm circumference of the cell. (B) Concentration of PBP4 in the cells. (C) Example of demographs showing fluorescence of PBP4 and its localization in cells sorted according to length of one experiment. The white line gives the limit of the cell lengths. (D) length of the cells. (E) Diameter of the cells. Δ*lpoA* (n = 5), Δ*lpoB* (n = 5), Δ*mrcA* (PBP1A, n = 2), Δ*mrcB* (PBP1B, n = 2). Each point is the average of 1000–2000 cells. Based on the one-way Anova the difference in midcell localization is significant (P = 0.009) while the difference in concentration of PBP4 is not significant (P = 0.0484).
(TIF)

S6 Fig PBP4 localizes between the outer membrane and the peptidoglycan layer in a Δ*nlpI* strain. (A) BW 25113 Δ*nlpI* cells were grown exponentially in TY medium at 37°C and fixed while shaking when at an OD600 of 0.3. The cells were harvested and split in three portions of which one was directly immunolabeled with anti-PBP4 antibodies that were pre-adsorbed to Δ*dacB* cells, the second was first treated with Triton X-100 and the last was treated with Triton X-100 and lysozyme and then immunolabeled. The scale bar equals 2 μm. (B) Map of PBP4 fluorescence sorted according to cell length of wild-type cells and Δ*nlpI* cells displayed at the same brightness and contrast values.
(TIF)

S7 Fig PBP4 localizes at site of inactive divisomes. MC4100 cells were grown to steady state in minimal glucose medium at 28°C and split in two parts. One part was 1:4 diluted in prewarmed medium without aztreonam, and the other part was 1:4 diluted in medium with 10 μg/ml aztreonam. The cells continued to grow for 0, 1, 2 or 3 mass doublings (MD) and were fixed and immunolabeled with antibodies specific for FtsZ or PBP4. Demographs with identical brightness and contrast of the FtsZ or the PBP4 fluorescence of cells grown in the absence or presence of aztreonam sorted according to length. The white line shows the borders of the cells. Number of analyzed cells per demograph for FtsZ and PBP4 were, 5084 and 3448 (0), 1430 and 1858 (1), 857 and 1630 (2) and 659 and 595 (3), respectively.
(TIF)

S8 Fig All PBP4 mutants are equally well expressed and only S62G is not able to bind Bocillin FL. Mutants expressed from plasmid without induction in the Δ*dacB* strain grown in TY at 37°C. **(**A) An immunoblot of membranes of PBP4 wild-type and variants. *This extra band is due to non-specificity of the primary antibody, which was for the purpose of immunoblotting not affinity purified. (B) Immunoblot of supernatant after pelleting the membrane of PBP4 wild-type and variants. (C) The corresponding gel where binding of the fluorescent β-lactam Bocillin FL is visible for all mutants apart from S62G. EP is empty plasmid, WT is wild-type. The other samples are PBP4 variants.
(TIF)

S9 Fig PBP4ΔD3 binds beta-lactam but is inactive against PG substrates. (A) PBP4 versions were incubated with the fluorescent β-lactam Bocillin FL with or without pre-incubation with Penicillin G (PenG), followed by SDS-PAGE analysis and detection of covalent Bocillin-PBP4 adducts by fluorescence scanner. PBP4 and PBPP4ΔD3, but not catalytically inactive PBP4(S62A), bound Bocillin FL. (B) Only wild-type PBP4, but not PBPP4ΔD3 or PBP4(S62A), was active in a DD-carboxypeptidase assay with UDP-Mur*N*Ac pentapeptide substrate. (C) PBP4 versions were incubated with muropeptides from BW25113 prior to their separation by high-performance liquid chromatography (HPLC). A control sample contained no enzyme. PBP4 DD-endopeptidase activity is demonstrated by the reduction in the dimer (TetraTetra) substrate peak and increase in the monomer (Tetra) product peak. PBPP4ΔD3 and PBP4(S62G) were inactive. (D) Quantification of the Tetra and TetraTetra peaks shown in panel C (top) and quantification of a similar analysis with PG sacculi (incubated with PBP4 versions or no enzyme), followed by generation of muropeptides and HPLC analysis (bottom).
(TIF)

S10 Fig The absence of domain 3 does not affect the dimerization, secondary structure, or PG binding of PBP4. (A) Analytical ultracentrifugation sedimentation velocity experiment of PBP4 and PBP4ΔD3 shows that both proteins are mainly dimers. The determined sedimentation coefficients (s_exp_) for the dimers fit well with the theoretical values (s_calc_) that were calculated from atomic coordinates of both protein dimers (pdb accession code for monomer is 2EX2). (B) PBP4 and PBP4ΔD3 show similar far UV circular dichroism spectra. (C) PBP4 and PBP4ΔD3 co-sediment with PG sacculi from BW25113 cells, but did not sediment in control samples without sacculi, demonstrating binding to PG. S, supernatant; W, wash fraction; P, pellet fraction.
(TIF)

S11 Fig Timing of the localization of FtsZ and FtsN in ΔdacB strain expressing PBP4 variants from plasmid. Cells were grown in minimal glucose medium at 28°C, fixed and immunolabeled against FtsZ and FtsN. (A) Graphical illustration of the meaning of t_0_ and t_1/2_. (B). Cell division cycle age timing of FtsZ and FtsN for the Δ*dacB* strain expressing PBP4 variants from plasmid without induction and its parental BW25113. Because BW25113 cannot be grown to steady state, the difference in divisome assembly of the PBP4 variant should be interpretated as not identical to that of the wild type in the case of S63G and D155A, ignoring the absolute numbers.
(TIF)

S12 Fig Interactions of PBP4 and PBPP4ΔD3 measured by microscale thermophoresis (MST). MST binding curves for interactions using different fluorescently labelled proteins (LpoA, LpoA^C^, Lpo^N^, NlpI or PBP1A) titrated against fixed concentrations of PBP4 or PBPP4ΔD3. Apparent K_D_ values are mean ± SD of three independent experiments and summarized in Fig 8A.
(TIF)

S13 Fig PBP4 or NlpI do not affect the activity of PBP1A or PBP1A/LpoA. Representative HPLC chromatograms of samples from an *in vitro* PG synthesis assay of PBP1A and radiolabelled Lipid II, in the presence of proteins indicated. The main PG products upon digestion with the muramidase cellosyl are the disaccharide pentapeptide (Penta) and the bis-disaccharide tetrapentapeptide (TetraPenta). The % peptides present in cross-links is quantified on the right side and presented as mean ± variation of two independent experiments.
(TIF)

S14 Fig PBP4 or NlpI do not affect the activity of PBP1A whereas LpoA reduces the activity of PBP4. (A) Summary of the results from an *in vitro* PG synthesis assay with PBP1A (in the presence or absence of LpoA) and radio-labelled lipid II substrate. The presence of NlpI, catalytically inactive PBP4(S62A) or both together, does not affect the cross-linkage of the PG product of a PBP1A or PBP1A/LpoA reaction (left side), and NlpI, PBP4(S62A) or both together, do not induce enhanced carboxypeptidase activity of PBP1A, which would be seen as a higher content of tetrapeptides (right side). Values are mean ± variation of two independent repeats. Example chromatograms are shown in S12B Fig. (B) The presence of LpoA modestly reduces the activity of PBP4 in a DD-carboxypeptidase assay using the substrate UDP-Mur*N*Ac pentapeptide (C) LpoA also reduces slightly the activity of PBP4 against PG sacculi from BW25113, as seen by the reduced digestion of TetraTetra upon a 30 min incubation period.
(TIF)

S15 Fig EnvC interacts with PBP4 and NlpI. (A) Interaction assays by MST with fluorescently labelled EnvC (Fl-EnvC), fluorescently labelled NlpI (Fl-NlpI), NlpI, PBP4, PBP4ΔD3 and LytM domain of EnvC (EnvC-LytM). The apparent K_D_ values are indicated. (B) Summary of interaction data. (C) Pulldown to Ni-NTA beads using oligohistidine-tagged-NlpI (His-NlpI) and untagged PBP4 and EnvC. Proteins were detected on a Coomassie Blue-stained SDS-PAGE. A, applied sample; E, elution sample.
(TIF)

S1 Table Morphological parameters LMC500 and LMC500Δ*dacB*. (DOCX)

S2 Table Phenotypes of *dacB*, amidases and regulators mutants. (DOCX)

S1 Data PBP4 localization in MC4100 and BW25113 belonging to Fig 2. (XLSX)

S2 Data PBP4 in ts strains belonging to Fig 3. (XLSX)

S3 Data PBP4 in delta mutants belonging to Figs 4 and S4. (XLSX)

S4 Data PBP4 in lpoAB and PBP1AB mutants belonging to S5 Fig. (XLSX)

S5 Data FtsZ and FtsN in LMC500 (a) belonging to Fig 6.
(XLSX)

S6 Data FtsZ and FtsN in LMC500 (b) belonging to Fig 6.
(XLSX)

S7 Data FtsZ and FtsN in LMC500 ΔdacB belonging to Fig 6. (XLSX)

S8 Data FtsZ and FtsN in BW25113 belonging to Fig 6. (XLSX)

S9 Data FtsZ and FtsN in BW25113 ΔdacB belonging to Fig 6. (XLSX)

S10 Data Powerpoint: ov2021_Plosgeneticspull down gels belonging to Fig 8. (PPTX)

S11 Data Antibody against PBP4 quality check belonging to S1 Fig. (XLSX)

S12 Data PBP4 in ami and NlpI deletion strains belonging to S3 Fig. (XLSX)

S13 Data PBP4 and aztreonam belonging to S7 Fig. (XLSX)

S14 Data PBP4 mutants in dacB (anti FtsZ, PBP4, FtsN) belonging to S11 Fig. (XLSX)

S15 Data Nov2021_PlosGeneticsMSTrawdatav1 belonging to S14 Fig. (XLSX)
