# Supplementary figures and images for: Early midcell localization of Escherichia coli PBP4 supports the function of peptidoglycan amidases
Source: PLoS Genet. 2022 May 23;18(5):e1010222. doi: 10.1371/journal.pgen.1010222 (PMC9166362; doi:10.1371/journal.pgen.1010222)

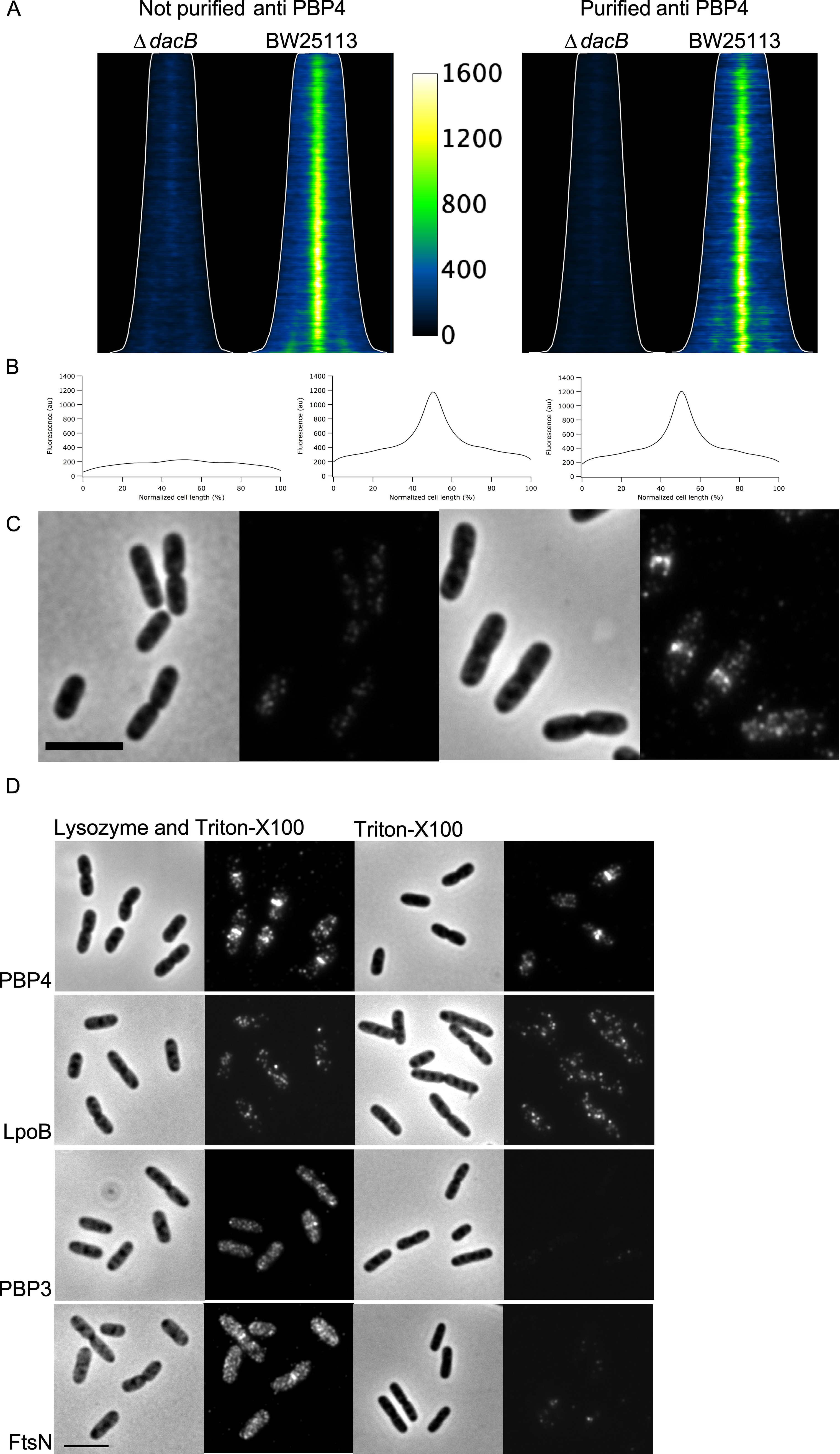

Supplement: S1 Fig — (A) Demograph with cells sorted according to length of PBP4 fluorescence of the BW25113ΔdacB (PBP4) strain, BW245113 (wt) strain before purification of the antibody and the BW25113ΔdacB strain and BW245113 strain after purification of the antibody. (B) Absolute fluorescence average profiles of cells from the BW25113 ΔdacB strain and the BW245113 strain before purification of the antibody and from the BW245113 strain after purification of the antibody (C) Phase contrast and corresponding fluorescence image of anti PBP4 immunolabeled BW25113ΔdacB (left) and wild-type cells using the supernatant of BW25113ΔdacB pre-adsorbed antibodies. The scale bar equals 5 μm. (D) BW25113 wild-type cells were grown in TY at 37°C and harvested in the exponential phase at an OD600 of 0.3 and fixed. Samples were divided into two aliquots and cells were immunolabeled with antibodies against PBP3, FtsN, LpoB and PBP4. The first aliquot was immunolabeled after permeabilizing the cell membrane with Triton X-100 (right: phase contrast and fluorescence images) and the second after permeabilizing cells with Triton X-100 and lysozyme that cleaves the glycan strands of the PG layer (left: phase contrast and fluorescence images). The scale bar equals 5 μm. PBP4 and LpoB, but not PBP3 or FtsN, are accessible without degrading the peptidoglycan layer. (TIF) [file pgen.1010222.s001.tif]

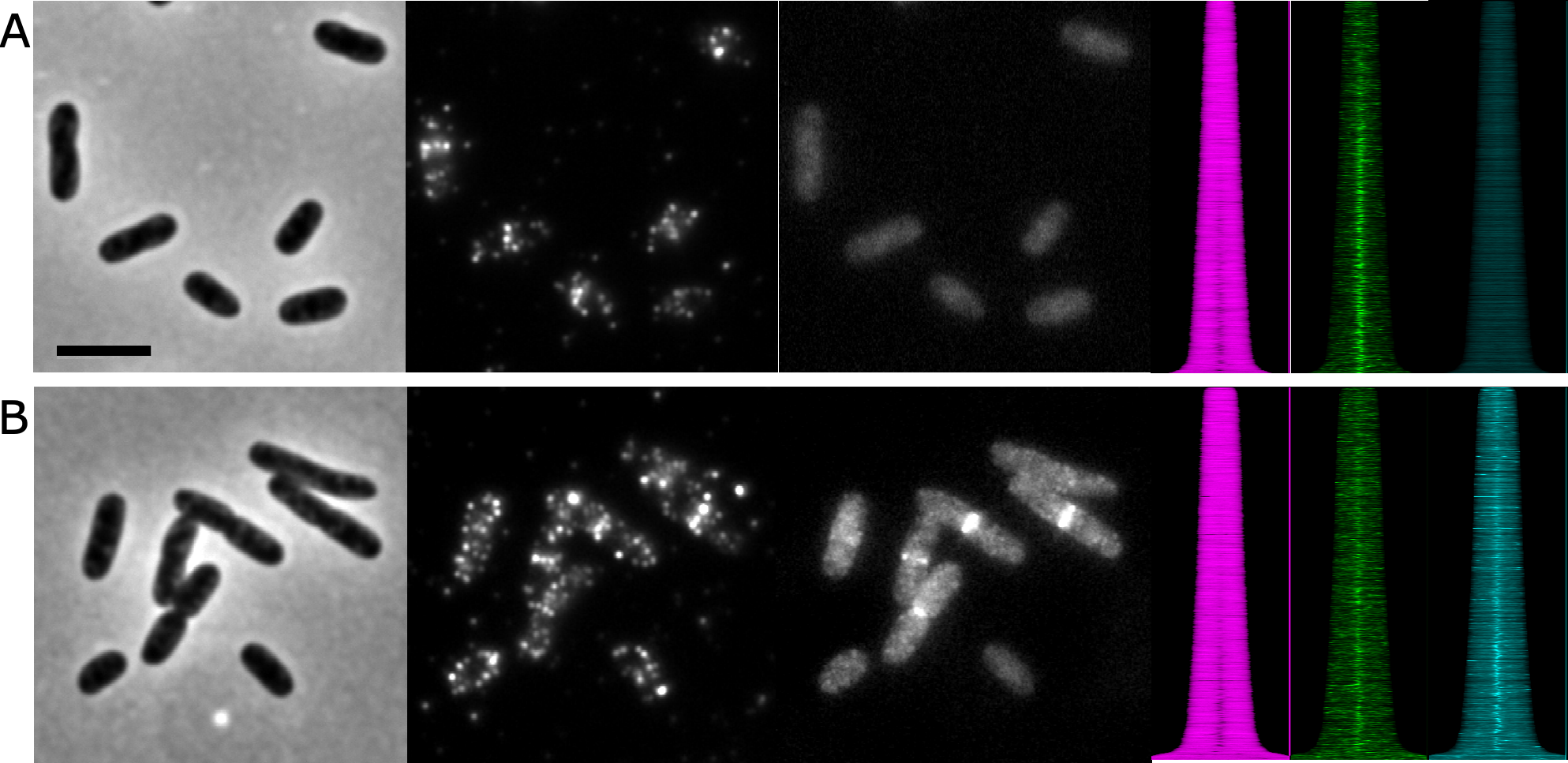

Supplement: S2 Fig — Strains were grown in LB at 37°C to an OD of 0.3 in the presence of 30 μM IPTG to induce expression of mNG-FtsE then fixed and immunolabeled with antibodies specific for PBP4. (A) LMC500 (1108 cells analyzed). (B) XL36 (LMC500::pTrc99downftsX, ΔftsE) with pXL110 mNG-FtsE(wt) (1189 cells analyzed). From left to right, phase contrast image, anti-PBP4 immunolabeling, mNG-FtsE, and the corresponding demograph of the diameter and the demograph of fluorescence of cells sorted according to length. Scale bar equals 5 μm. (TIF) [file pgen.1010222.s002.tif]

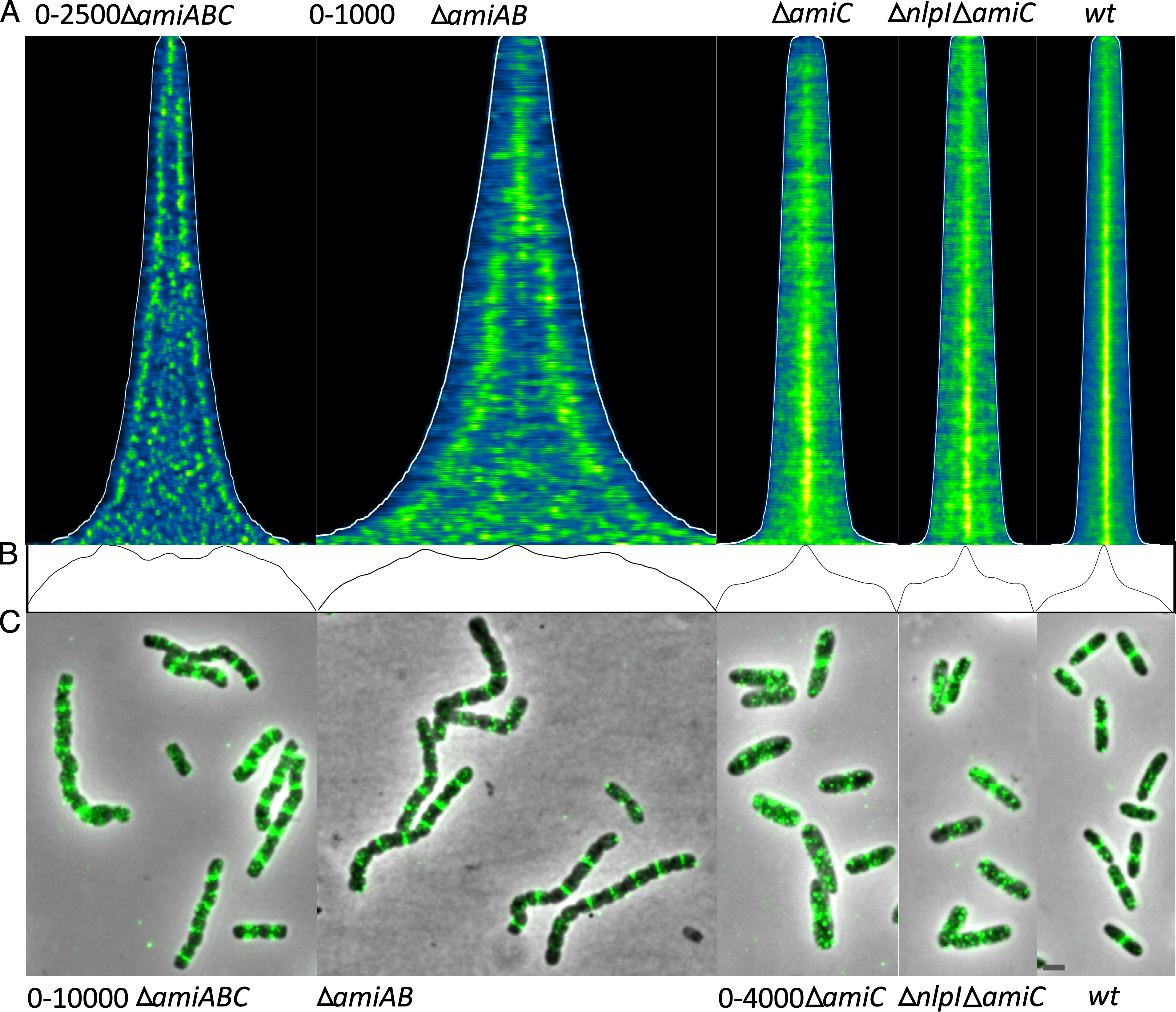

Supplement: S3 Fig — Isogenic strains of the wild-type strain MC1061 were grown in LB at 37°C to an OD600 of 0.3, fixed and labeled with specific antibodies against PBP4. (A) Demographs of the fluorescence distribution in cell sorted according to their length. The numbers above the graph indicate the adjustment of the brightness and the contrast, which had to be different for the double and triple-ami deletion strain as they produced more PBP4 than the other strains. (B) peak normalized fluorescence along the cell length of all cells in the demograph. (C) Images showing examples of the localization of PBP4 (green) overlayed with the phase contrast of the corresponding cells. The number of cells analyzed were 3311 for MC1061 (wt), 1691 for ΔamiC, 1865 for ΔnlpI AmiC, 926 for ΔamiAB for 484 for ΔamiABC. The scale bar equals 2 μm. (TIF) [file pgen.1010222.s003.tif]

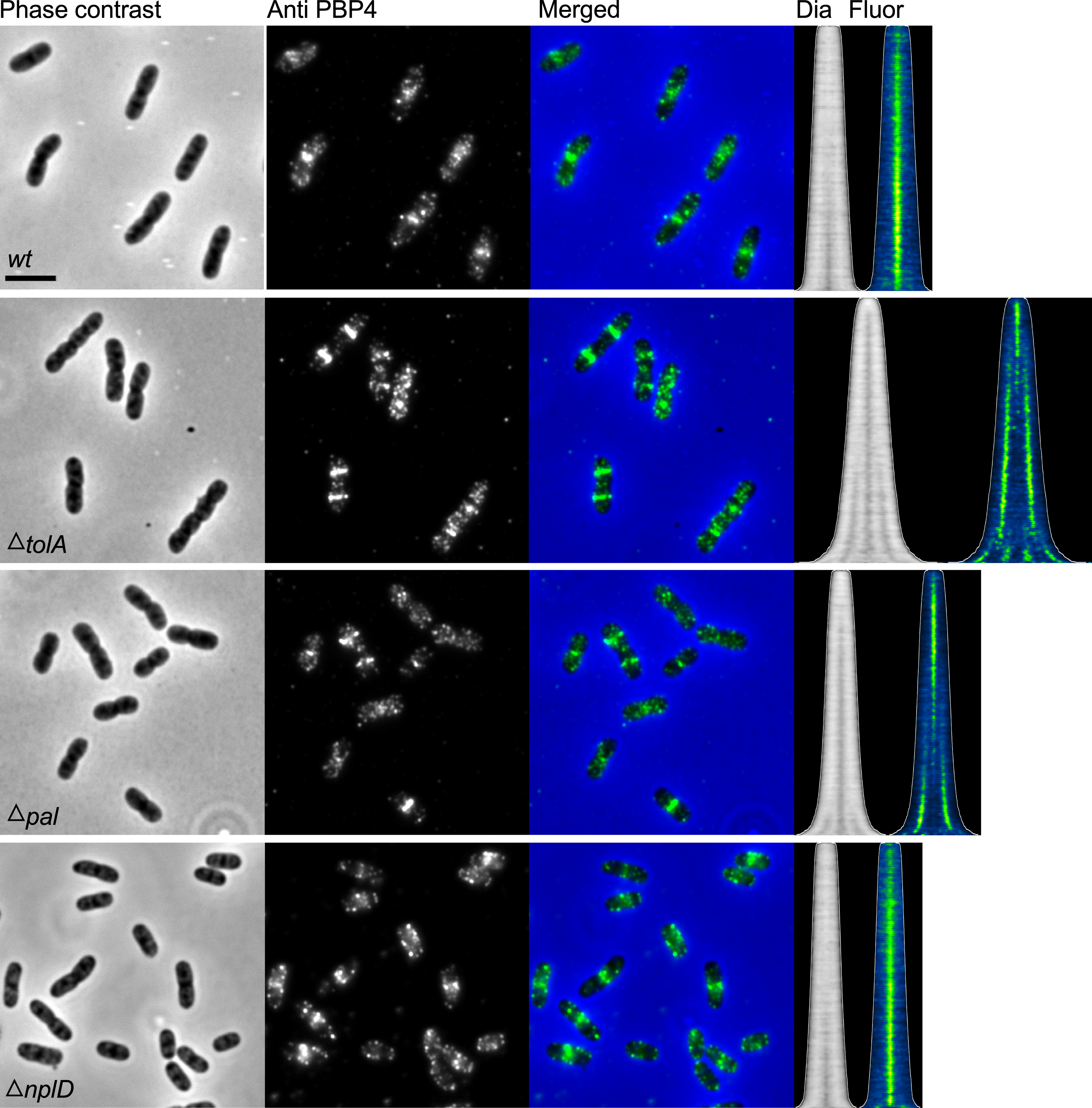

Supplement: S4 Fig — Isogenic strains of the wild-type strain BW25113 were grown in LB at 37°C to an OD600 of 0.3, fixed and labeled with specific antibodies against PBP4. From left to right, the phase contrast, corresponding fluorescence image of the PBP4 labeling, and the merged former two images, demograph of diameters (Dia) and demograph of fluorescence (Fluor) PBP4 localization where cells are sorted according to their cell length are shown. The number of cells analyzed were 1053 for BW25113 (wt), 918 for ΔtolA, 1314 for Δpal, and 1600 for ΔnlpD. The scale bar equals 5 μm. (TIF) [file pgen.1010222.s004.tif]

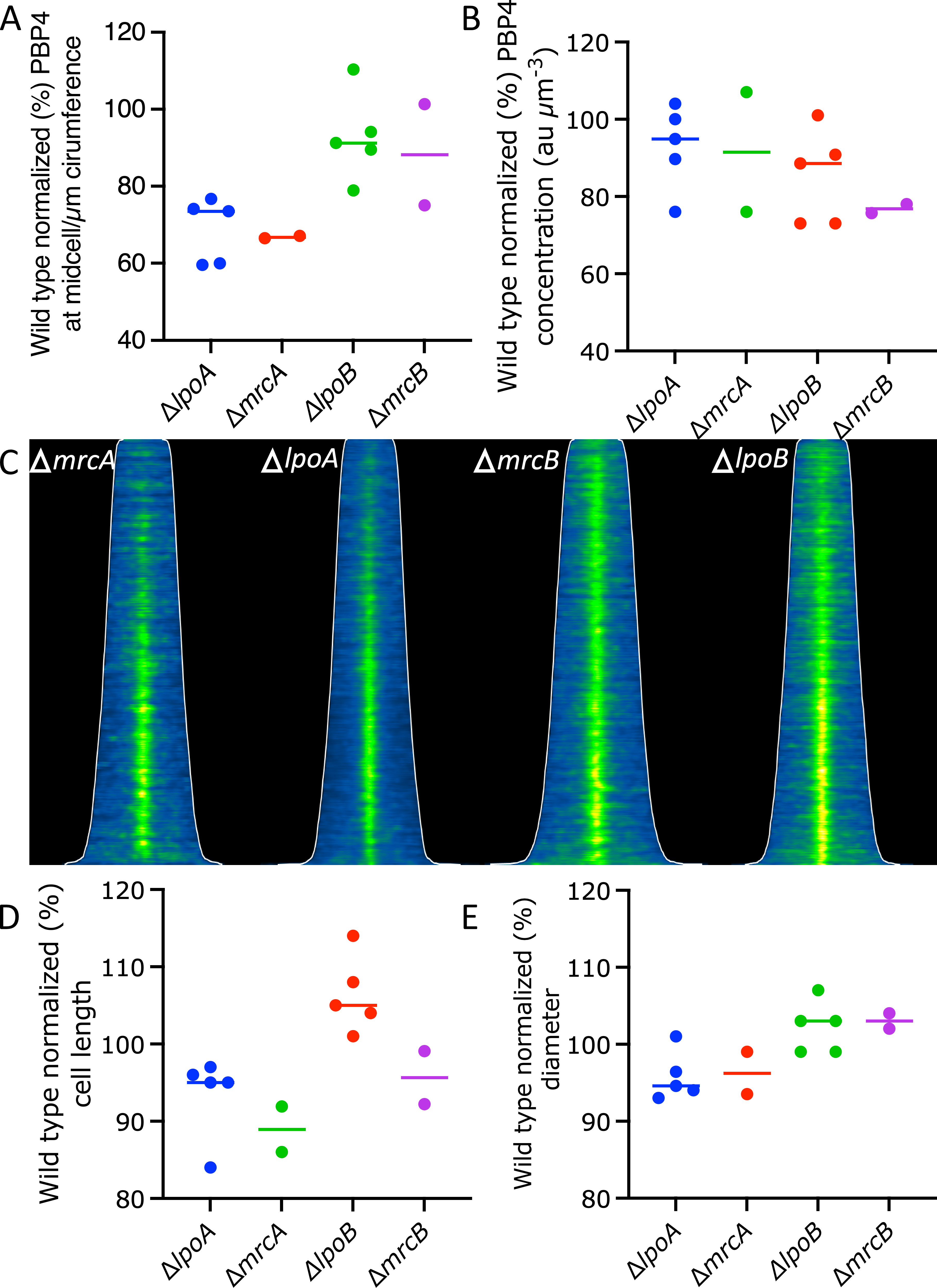

Supplement: S5 Fig — Cells were grown exponentially to an OD of 0.3 in TY at 37°C, then fixed and immunolabeled with antibodies against PBP4. Because fluorescence imaging by microscopes is usually not directly comparable between different experiments, all results in each experiment were normalized to the parental strain BW25113. (A) PBP4 fluorescence at midcell per μm circumference of the cell. (B) Concentration of PBP4 in the cells. (C) Example of demographs showing fluorescence of PBP4 and its localization in cells sorted according to length of one experiment. The white line gives the limit of the cell lengths. (D) length of the cells. (E) Diameter of the cells. ΔlpoA (n = 5), ΔlpoB (n = 5), ΔmrcA (PBP1A, n = 2), ΔmrcB (PBP1B, n = 2). Each point is the average of 1000–2000 cells. Based on the one-way Anova the difference in midcell localization is significant (P = 0.009) while the difference in concentration of PBP4 is not significant (P = 0.0484). (TIF) [file pgen.1010222.s005.tif]

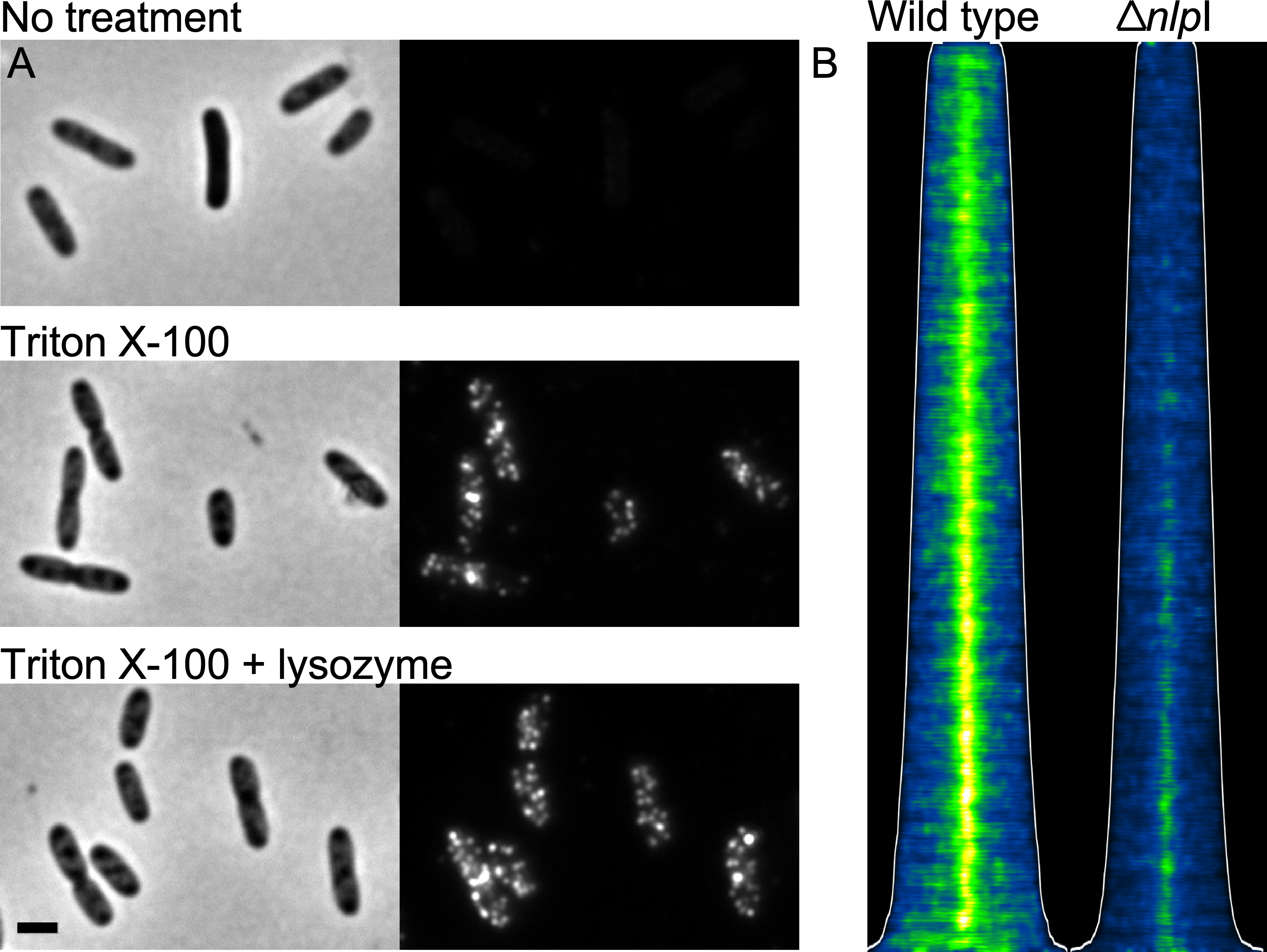

Supplement: S6 Fig — (A) BW 25113 ΔnlpI cells were grown exponentially in TY medium at 37°C and fixed while shaking when at an OD600 of 0.3. The cells were harvested and split in three portions of which one was directly immunolabeled with anti-PBP4 antibodies that were pre-adsorbed to ΔdacB cells, the second was first treated with Triton X-100 and the last was treated with Triton X-100 and lysozyme and then immunolabeled. The scale bar equals 2 μm. (B) Map of PBP4 fluorescence sorted according to cell length of wild-type cells and ΔnlpI cells displayed at the same brightness and contrast values. (TIF) [file pgen.1010222.s006.tif]

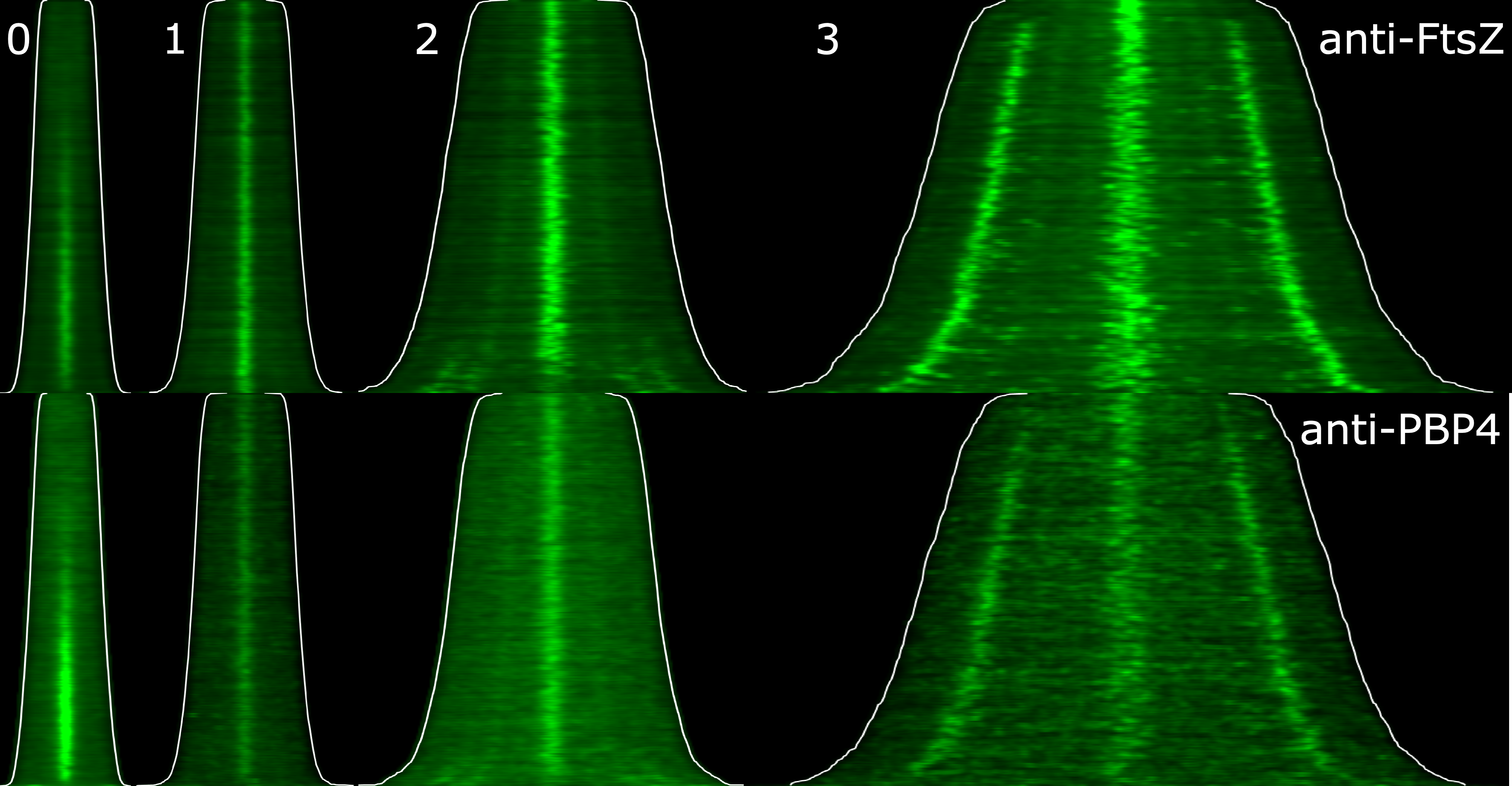

Supplement: S7 Fig — MC4100 cells were grown to steady state in minimal glucose medium at 28°C and split in two parts. One part was 1:4 diluted in prewarmed medium without aztreonam, and the other part was 1:4 diluted in medium with 10 μg/ml aztreonam. The cells continued to grow for 0, 1, 2 or 3 mass doublings (MD) and were fixed and immunolabeled with antibodies specific for FtsZ or PBP4. Demographs with identical brightness and contrast of the FtsZ or the PBP4 fluorescence of cells grown in the absence or presence of aztreonam sorted according to length. The white line shows the borders of the cells. Number of analyzed cells per demograph for FtsZ and PBP4 were, 5084 and 3448 (0), 1430 and 1858 (1), 857 and 1630 (2) and 659 and 595 (3), respectively. (TIF) [file pgen.1010222.s007.tif]

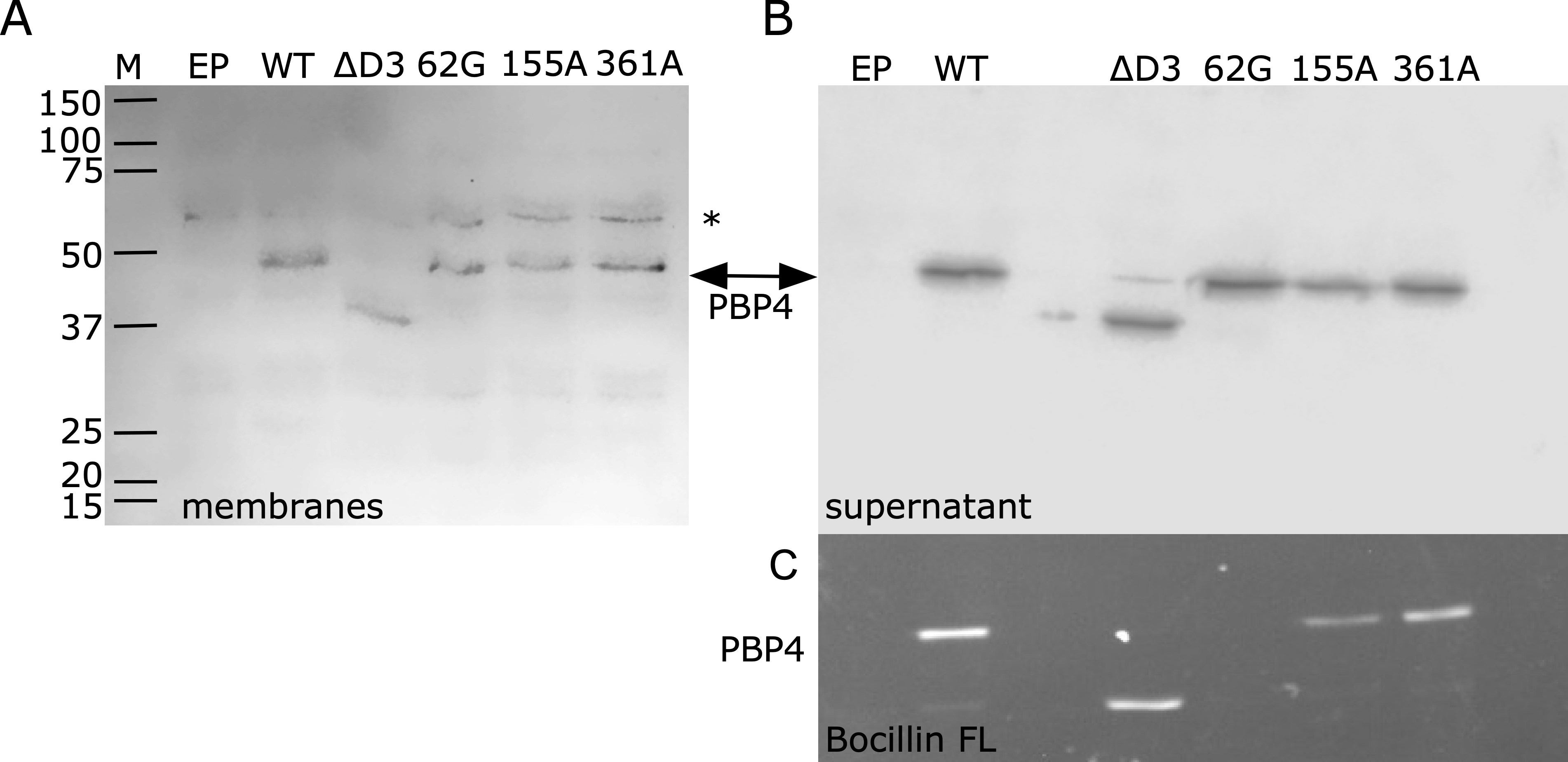

Supplement: S8 Fig — Mutants expressed from plasmid without induction in the ΔdacB strain grown in TY at 37°C. (A) An immunoblot of membranes of PBP4 wild-type and variants. *This extra band is due to non-specificity of the primary antibody, which was for the purpose of immunoblotting not affinity purified. (B) Immunoblot of supernatant after pelleting the membrane of PBP4 wild-type and variants. (C) The corresponding gel where binding of the fluorescent β-lactam Bocillin FL is visible for all mutants apart from S62G. EP is empty plasmid, WT is wild-type. The other samples are PBP4 variants. (TIF) [file pgen.1010222.s008.tif]

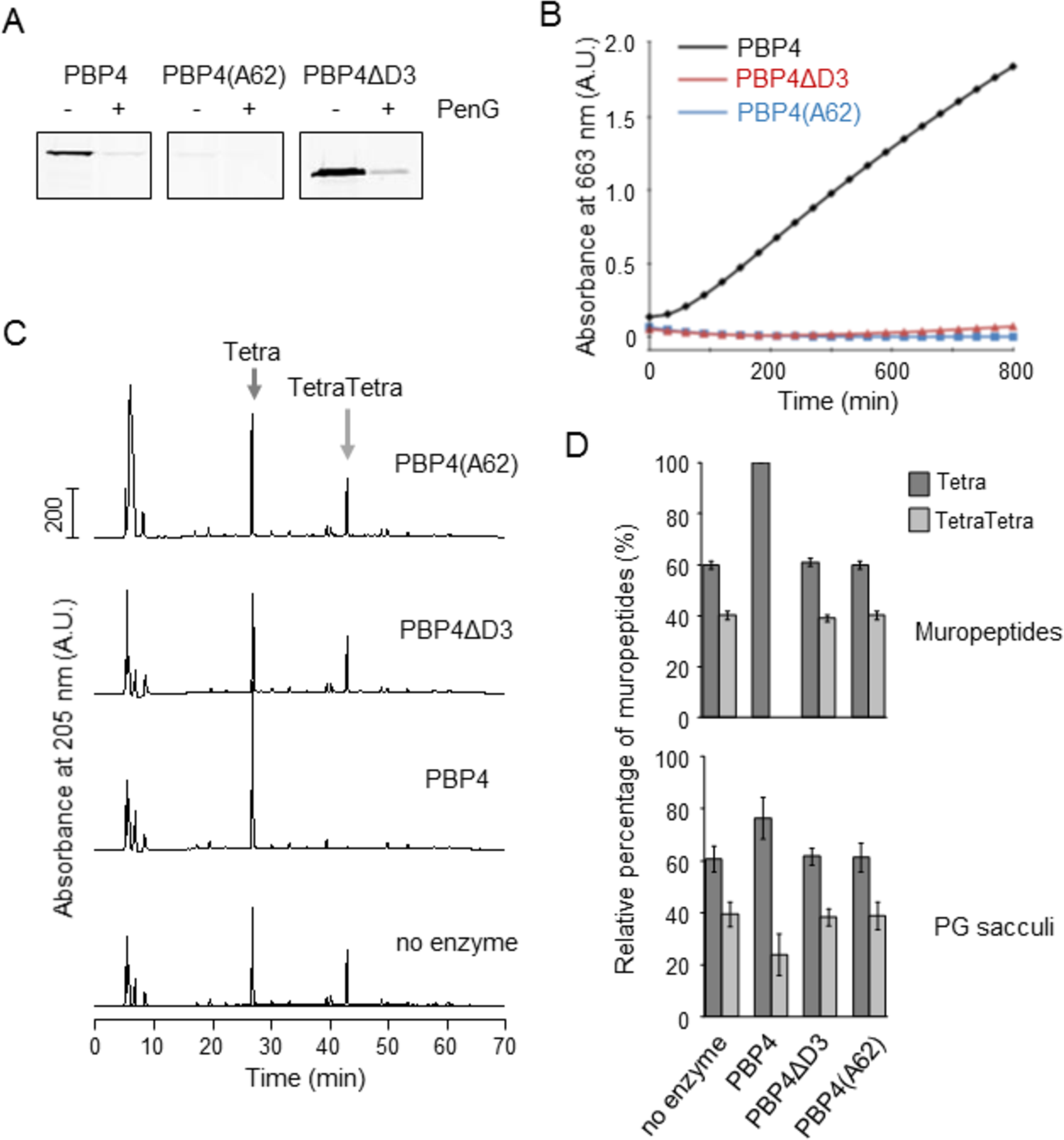

Supplement: S9 Fig — (A) PBP4 versions were incubated with the fluorescent β-lactam Bocillin FL with or without pre-incubation with Penicillin G (PenG), followed by SDS-PAGE analysis and detection of covalent Bocillin-PBP4 adducts by fluorescence scanner. PBP4 and PBPP4ΔD3, but not catalytically inactive PBP4(S62A), bound Bocillin FL. (B) Only wild-type PBP4, but not PBPP4ΔD3 or PBP4(S62A), was active in a DD-carboxypeptidase assay with UDP-MurNAc pentapeptide substrate. (C) PBP4 versions were incubated with muropeptides from BW25113 prior to their separation by high-performance liquid chromatography (HPLC). A control sample contained no enzyme. PBP4 DD-endopeptidase activity is demonstrated by the reduction in the dimer (TetraTetra) substrate peak and increase in the monomer (Tetra) product peak. PBPP4ΔD3 and PBP4(S62G) were inactive. (D) Quantification of the Tetra and TetraTetra peaks shown in panel C (top) and quantification of a similar analysis with PG sacculi (incubated with PBP4 versions or no enzyme), followed by generation of muropeptides and HPLC analysis (bottom). (TIF) [file pgen.1010222.s009.tif]

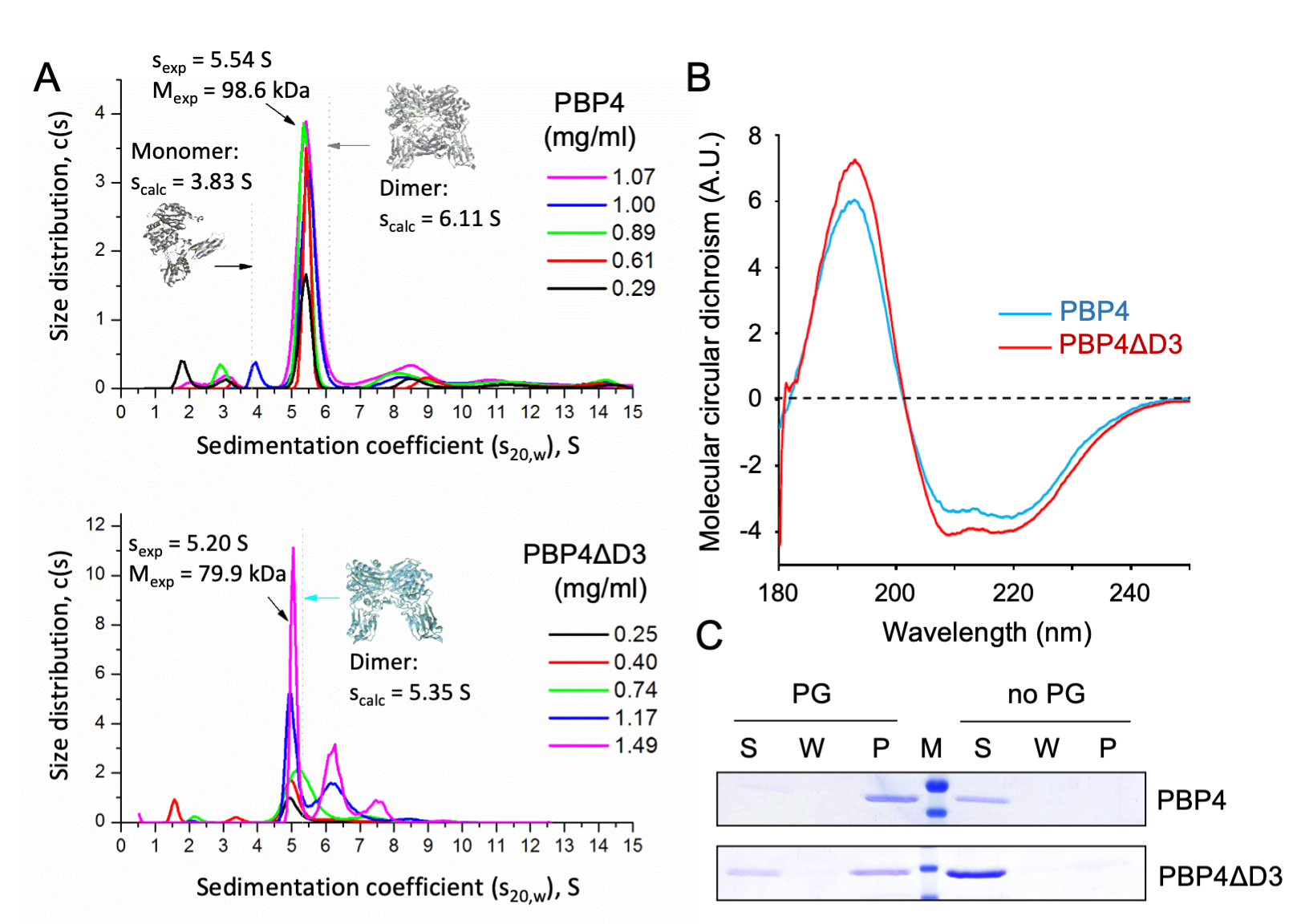

Supplement: S10 Fig — (A) Analytical ultracentrifugation sedimentation velocity experiment of PBP4 and PBP4ΔD3 shows that both proteins are mainly dimers. The determined sedimentation coefficients (sexp) for the dimers fit well with the theoretical values (scalc) that were calculated from atomic coordinates of both protein dimers (pdb accession code for monomer is 2EX2). (B) PBP4 and PBP4ΔD3 show similar far UV circular dichroism spectra. (C) PBP4 and PBP4ΔD3 co-sediment with PG sacculi from BW25113 cells, but did not sediment in control samples without sacculi, demonstrating binding to PG. S, supernatant; W, wash fraction; P, pellet fraction. (TIF) [file pgen.1010222.s010.tif]

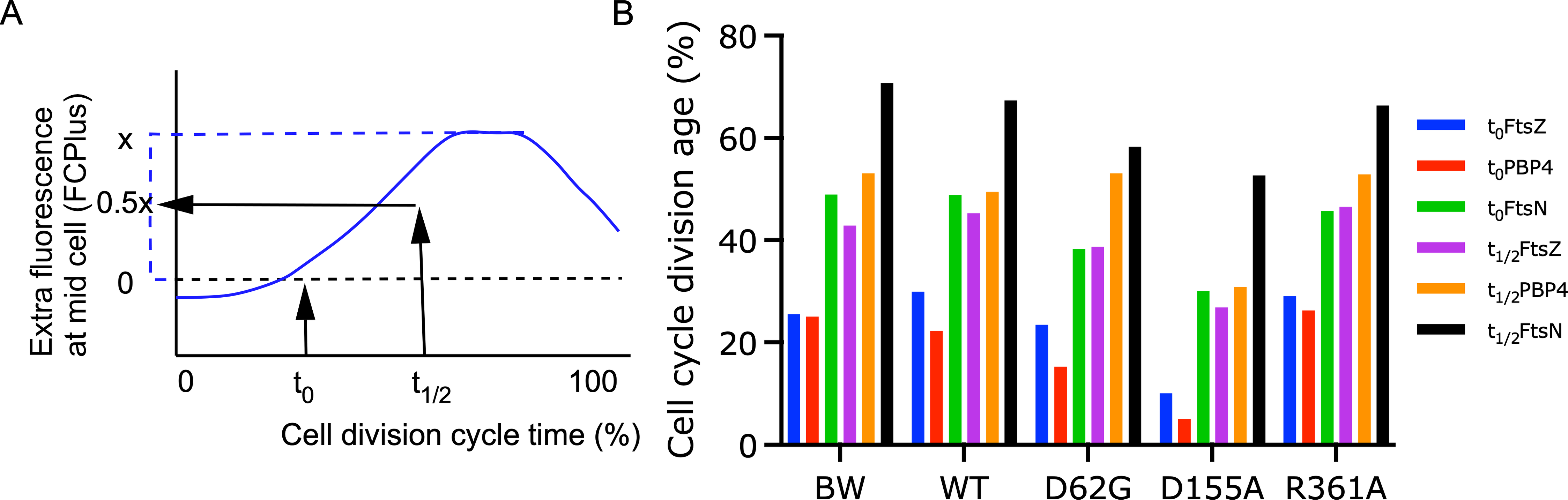

Supplement: S11 Fig — Cells were grown in minimal glucose medium at 28°C, fixed and immunolabeled against FtsZ and FtsN. (A) Graphical illustration of the meaning of t0 and t1/2. (B). Cell division cycle age timing of FtsZ and FtsN for the ΔdacB strain expressing PBP4 variants from plasmid without induction and its parental BW25113. Because BW25113 cannot be grown to steady state, the difference in divisome assembly of the PBP4 variant should be interpretated as not identical to that of the wild type in the case of S63G and D155A, ignoring the absolute numbers. (TIF) [file pgen.1010222.s011.tif]

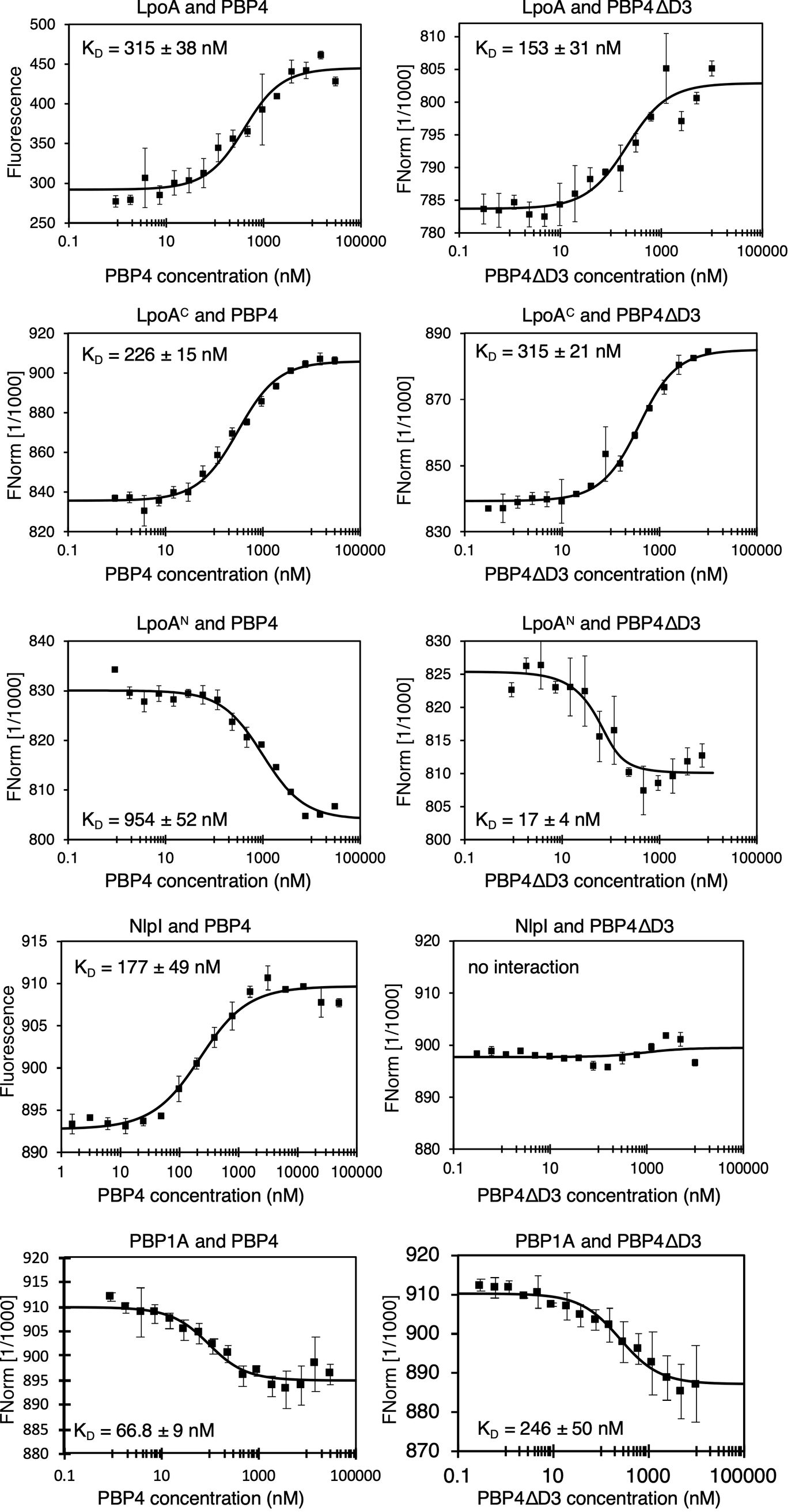

Supplement: S12 Fig — MST binding curves for interactions using different fluorescently labelled proteins (LpoA, LpoAC, LpoN, NlpI or PBP1A) titrated against fixed concentrations of PBP4 or PBPP4ΔD3. Apparent KD values are mean ± SD of three independent experiments and summarized in Fig 8A. (TIF) [file pgen.1010222.s012.tif]

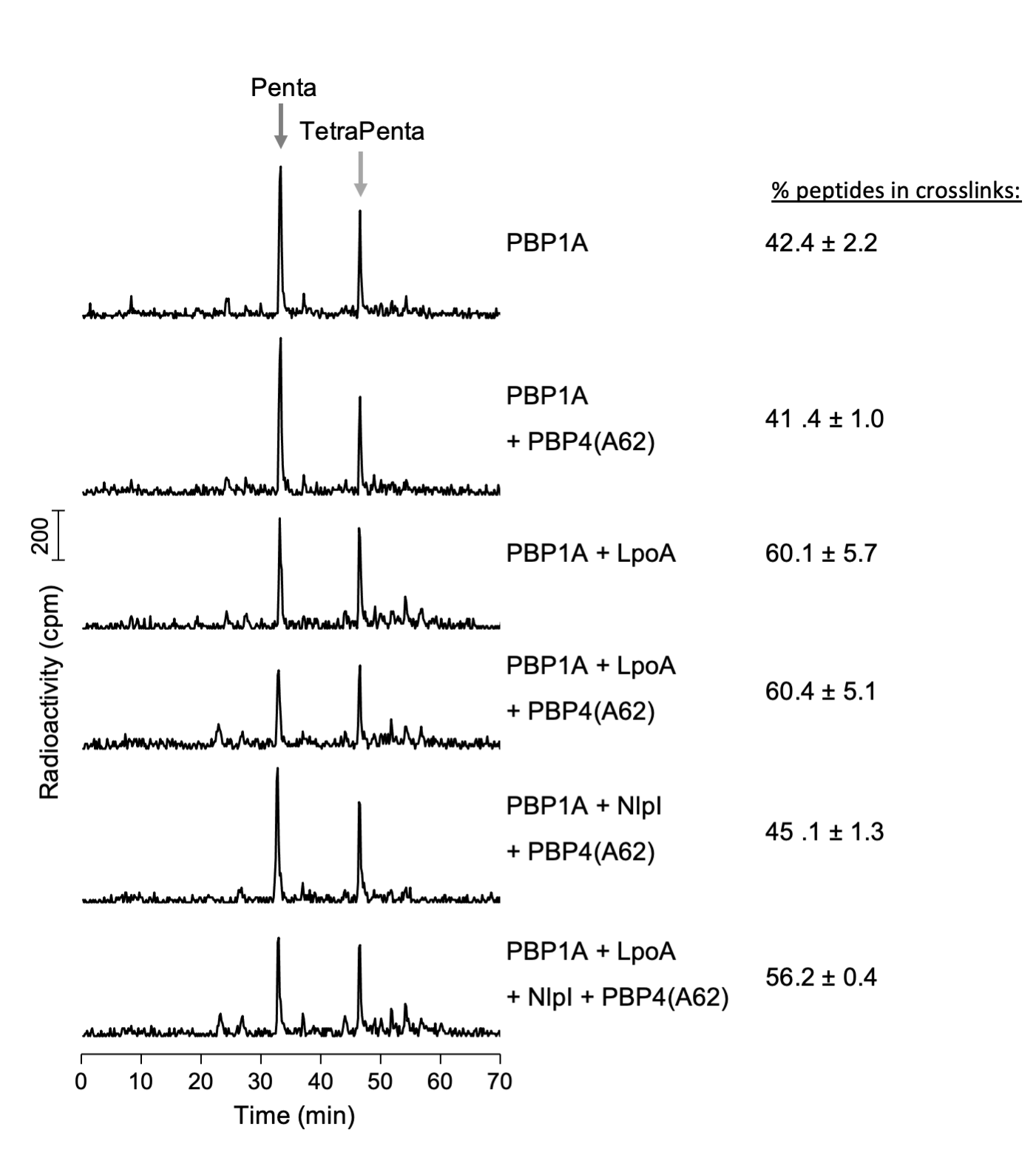

Supplement: S13 Fig — Representative HPLC chromatograms of samples from an in vitro PG synthesis assay of PBP1A and radiolabelled Lipid II, in the presence of proteins indicated. The main PG products upon digestion with the muramidase cellosyl are the disaccharide pentapeptide (Penta) and the bis-disaccharide tetrapentapeptide (TetraPenta). The % peptides present in cross-links is quantified on the right side and presented as mean ± variation of two independent experiments. (TIF) [file pgen.1010222.s013.tif]

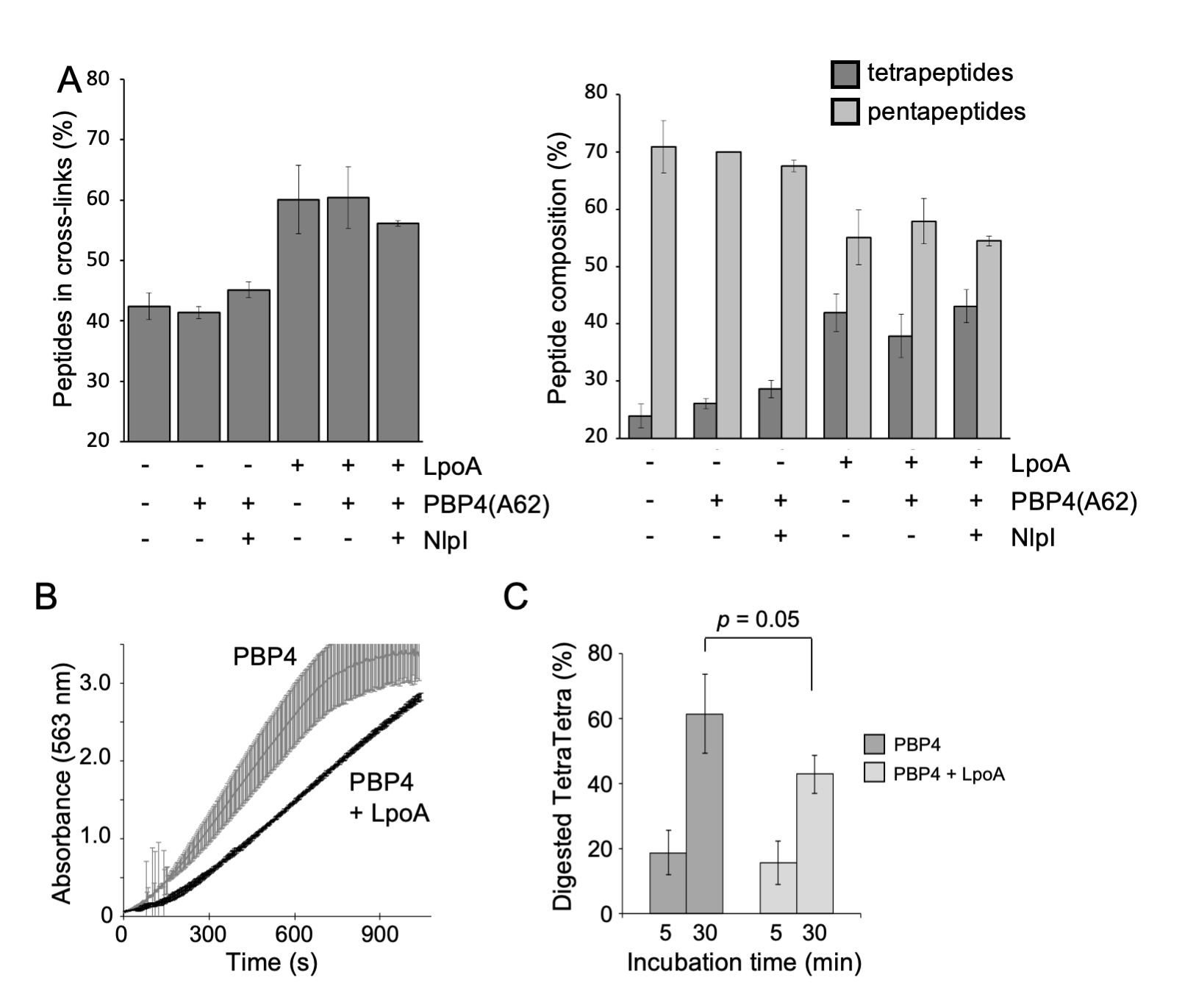

Supplement: S14 Fig — (A) Summary of the results from an in vitro PG synthesis assay with PBP1A (in the presence or absence of LpoA) and radio-labelled lipid II substrate. The presence of NlpI, catalytically inactive PBP4(S62A) or both together, does not affect the cross-linkage of the PG product of a PBP1A or PBP1A/LpoA reaction (left side), and NlpI, PBP4(S62A) or both together, do not induce enhanced carboxypeptidase activity of PBP1A, which would be seen as a higher content of tetrapeptides (right side). Values are mean ± variation of two independent repeats. Example chromatograms are shown in S12B Fig. (B) The presence of LpoA modestly reduces the activity of PBP4 in a DD-carboxypeptidase assay using the substrate UDP-MurNAc pentapeptide (C) LpoA also reduces slightly the activity of PBP4 against PG sacculi from BW25113, as seen by the reduced digestion of TetraTetra upon a 30 min incubation period. (TIF) [file pgen.1010222.s014.tif]

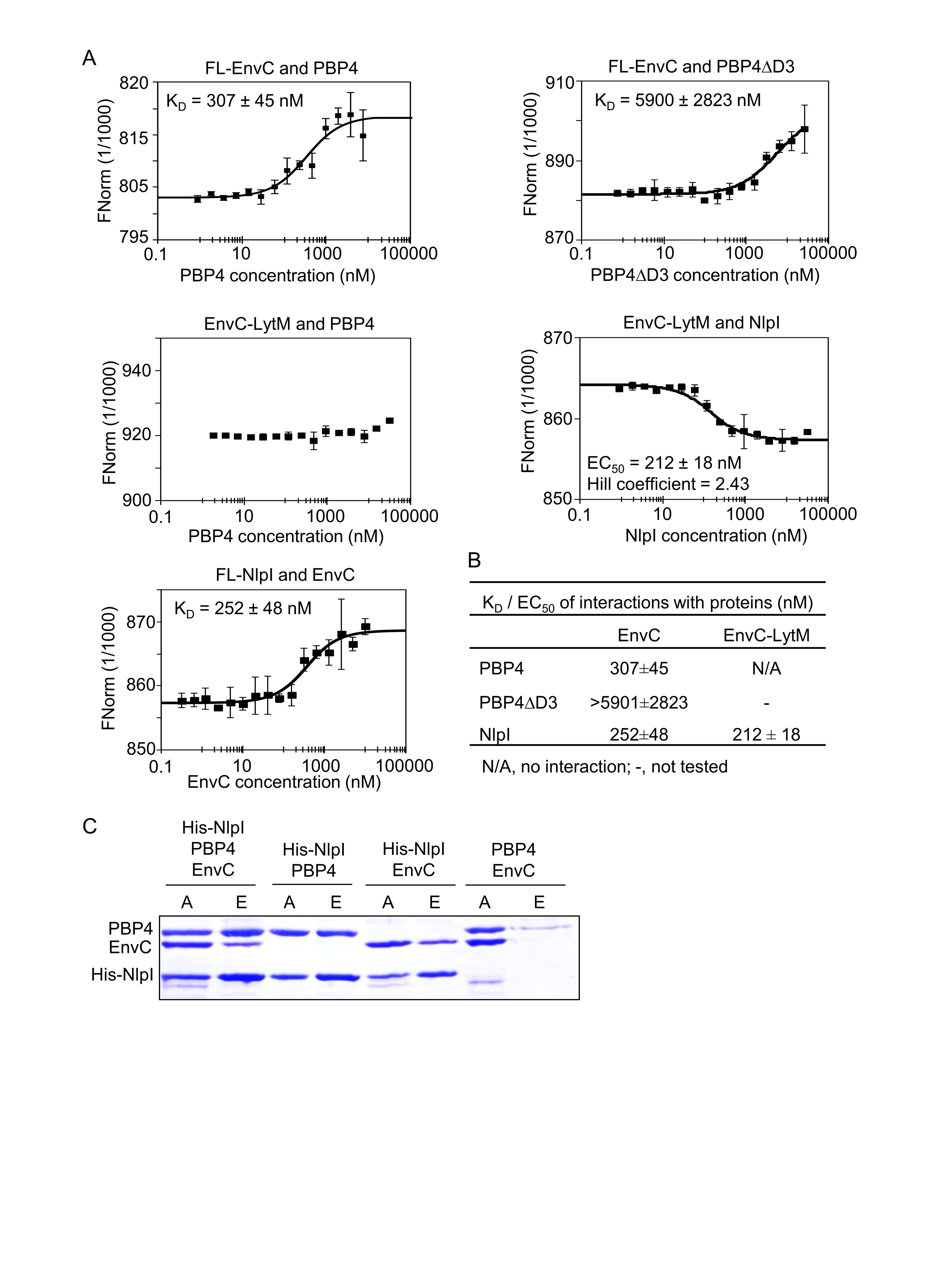

Supplement: S15 Fig — (A) Interaction assays by MST with fluorescently labelled EnvC (Fl-EnvC), fluorescently labelled NlpI (Fl-NlpI), NlpI, PBP4, PBP4ΔD3 and LytM domain of EnvC (EnvC-LytM). The apparent KD values are indicated. (B) Summary of interaction data. (C) Pulldown to Ni-NTA beads using oligohistidine-tagged-NlpI (His-NlpI) and untagged PBP4 and EnvC. Proteins were detected on a Coomassie Blue-stained SDS-PAGE. A, applied sample; E, elution sample. (TIF) [file pgen.1010222.s015.tif]
