# Supplementary material for: Early midcell localization of Escherichia coli PBP4 supports the function of peptidoglycan amidases
Source: PLoS Genet. 2022 May 23;18(5):e1010222. doi: 10.1371/journal.pgen.1010222 (PMC9166362; doi:10.1371/journal.pgen.1010222)
Supplement: S1 Table — (DOCX) [file pgen.1010222.s016.docx]

**S1 Table. Morphological parameters LMC500 and LMC500Δ*dacB***

| **Parameter** | **LMC500** | **n** | **LMC500Δ*dacB*** | **n** | **p** |
| --- | --- | --- | --- | --- | --- |
| Length (μm) | 2.57 ± 0.07 | 8 | 2.58 ± 0.09 | 12 | 0.79 |
| Diameter (μm) | 0.89 ± 0.09 | 8 | 0.91 ± 0.19 | 12 | 0.73 |
| Constrictions (%) | 16.9 ± 1.8 | 8 | 17.4 ± 1.3 | 12 | 0.53 |

n = number of biological replicates, P from a two tailed T-test to verify whether the means are significant different. The mass doubling time of both strains was 85 min.
