# Supplementary material for: Early midcell localization of Escherichia coli PBP4 supports the function of peptidoglycan amidases
Source: PLoS Genet. 2022 May 23;18(5):e1010222. doi: 10.1371/journal.pgen.1010222 (PMC9166362; doi:10.1371/journal.pgen.1010222)
Supplement: S2 Table — (DOCX) [file pgen.1010222.s017.docx]

S2 Table. Phenotypes of *dacB*, amidases and regulators mutants

| Strain | | Relevant genotype | No. of cells^1^ | Total length (µm)^2^ | Avg length (µm)^3^ | Total width (µm) | Avg width (µm) | Total no. of septa^4^ | Length/septum (µm)^5^ | | | Length/  Segment (µm)^6^ | No. of septa/cell | | | No. of cells chaining^7^ | Percentage of cells chaining^8^ | |
| --- | --- | --- | --- | --- | --- | --- | --- | --- | --- | --- | --- | --- | --- | --- | --- | --- | --- | --- |
| MB01064 | | WT | 131 | 595.1 | 4.5 | 135.0 | 1.0 | 54 | 11.0 | | | 3.2 | 0.4 | | | 0.0 | 0.0 | |
| NT10022 | | ∆*dacB* | 252 | 1078.2 | 4.3 | 258.3 | 1.0 | 119 | 9.1 | | | 2.9 | 0.5 | | | 2 | 0.8 | |
| MB01119 | | ∆*amiA* | 194 | 1035.8 | 5.3 | 204.7 | 1.1 | 112 | 9.2 | | | 3.4 | 0.6 | | | 1 | 0.5 | |
| MB01120 | | ∆*amiB* | 173 | 854.8 | 4.9 | 181.7 | 1.1 | 85 | 10.1 | | | 3.3 | 0.5 | | | 0 | 0.0 | |
| MB01121 | ∆*amiC* | | 322 | 1622.9 | 5.0 | 334.8 | 1.0 | 162 | 10.0 | | | 3.4 | 0.5 | | | 2 | 0.6 | |
| MB01051 | ∆*nlpD* | | 223 | 1041.6 | 4.7 | 230.9 | 1.0 | 91 | 11.4 | | | 3.3 | 0.4 | | | 1 | 0.4 | |
| MB01122 | ∆*envC* | | 208 | 1761.8 | 8.5 | 199.3 | 1.0 | 313 | 5.6 | | | 3.4 | 1.5 | | | 71 | 34.1 | |
| NT10274 | ∆*amiA* ∆*amiB* | | 100 | 1282.4 | 12.8 | 91.0 | 0.9 | 266 | 4.8 | | | 3.5 | 2.7 | | | 65 | 65.0 | |
| MB01154 | ∆*amiA* ∆*dacB* | | 245 | 990.2 | 4.0 | 240.6 | 1.0 | 129 | 7.7 | | | 2.6 | 0.5 | | | 2 | 0.8 | |
| MB01155 | ∆*amiB* ∆*dacB* | | 248 | 887.1 | 3.6 | 250.1 | 1.0 | 110 | 8.1 | | | 2.5 | 0.4 | | | 2 | 0.8 | |
| MB01156 | ∆*amiC* ∆*dacB* | | 196 | 897.0 | 4.6 | 203.5 | 1.0 | 69 | | 13.0 | 3.4 | | | 0.4 | 1 | | | 0.5 |
| MB01157 | ∆*envC* ∆*dacB* | | 105 | 1900.9 | 18.1 | 115.2 | 1.1 | 275 | | 6.9 | 5.0 | | | 2.6 | 81 | | | 77.1 |
| MB01158 | ∆*nlpD* ∆*dacB* | | 250 | 1192.7 | 4.8 | 262.9 | 1.1 | 121 | | 9.9 | 3.2 | | | 0.5 | 1 | | | 0.4 |
| MB01159 | ∆*amiA* ∆*amiB* ∆*dacB* | | 109 | 1267.7 | 11.6 | 105.3 | 1.0 | 273 | | 4.6 | 3.3 | | | 2.5 | 70 | | | 64.2 |

1 All cells were considered single cells independent of the number of segments.

2 Total length means cumulative length of all cells measured.

3 Refers to the total length/number of cells.

4 Septa are considered as any membrane constrictions or completed membrane septa in cell chains.

5 Length/septum indicates the total length/total number of septa. It shows the frequency at which septa are detected. The number is much lower in chaining cells because septa persist for an abnormally long time.

6 The number of cell segments refers to the number of cells plus the number of septa. The “length/segment” is the total length/total number of segments. In normal (nonchaining) cells, this measurement is similar to the average cell length (pole-pole distance), but the value is smaller because predivisional cells contain two segments and are counted as two cells instead of one (i.e., some pole-to-septa measurements are taken into account, as well as pole-pole measurements). In chaining cells, the length/segment measurement refers mainly to the distance between adjacent septa.

7 Cells with more than one septum are considered chaining cells.

8 Percentage of chaining cells related to the total no. of cells.
