# Supplementary material for: Early midcell localization of Escherichia coli PBP4 supports the function of peptidoglycan amidases
Source: PLoS Genet. 2022 May 23;18(5):e1010222. doi: 10.1371/journal.pgen.1010222 (PMC9166362; doi:10.1371/journal.pgen.1010222)
Supplement: S10 Data — (PPTX) [file pgen.1010222.s027.pptx]

## Slide 1
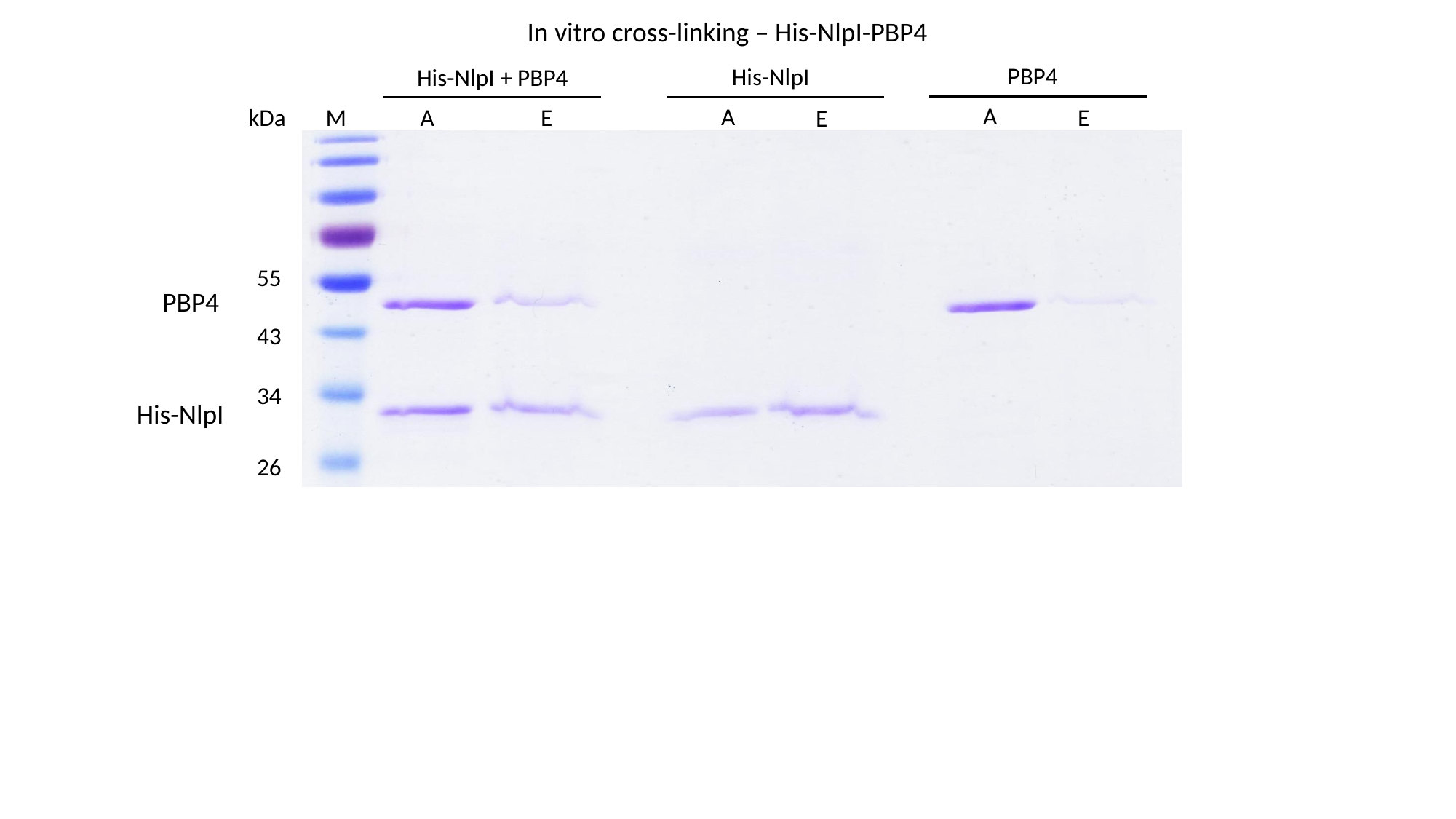

In vitro cross-linking – His-NlpI-PBP4
PBP4
His-NlpI
His-NlpI + PBP4
A
A
E
A
kDa
M
E
E
55
PBP4
43
34
His-NlpI
26

## Slide 2
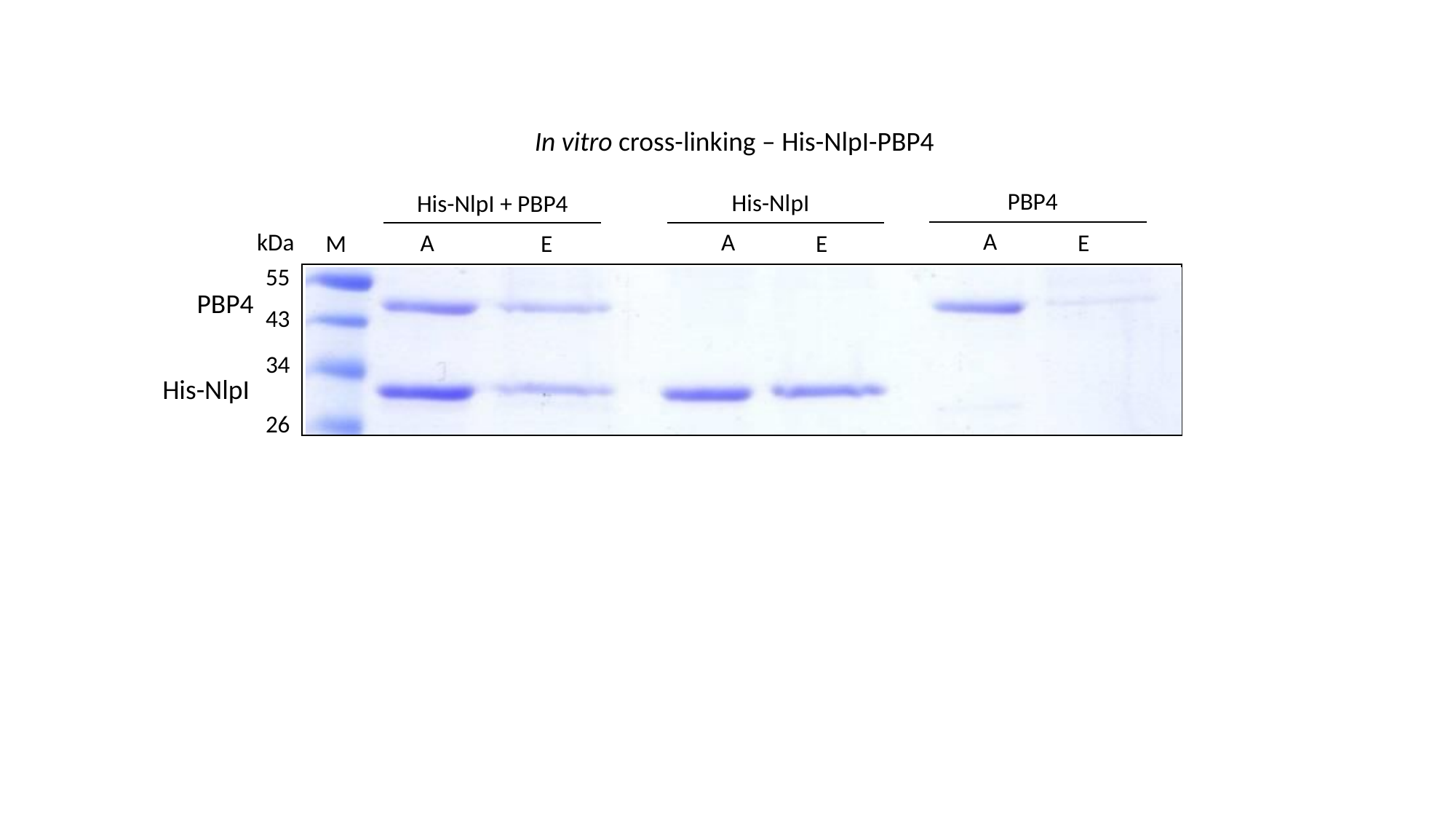

In vitro cross-linking – His-NlpI-PBP4
PBP4
His-NlpI
His-NlpI + PBP4
A
kDa
A
E
A
M
E
E
55
PBP4
43
34
His-NlpI
26

## Slide 3
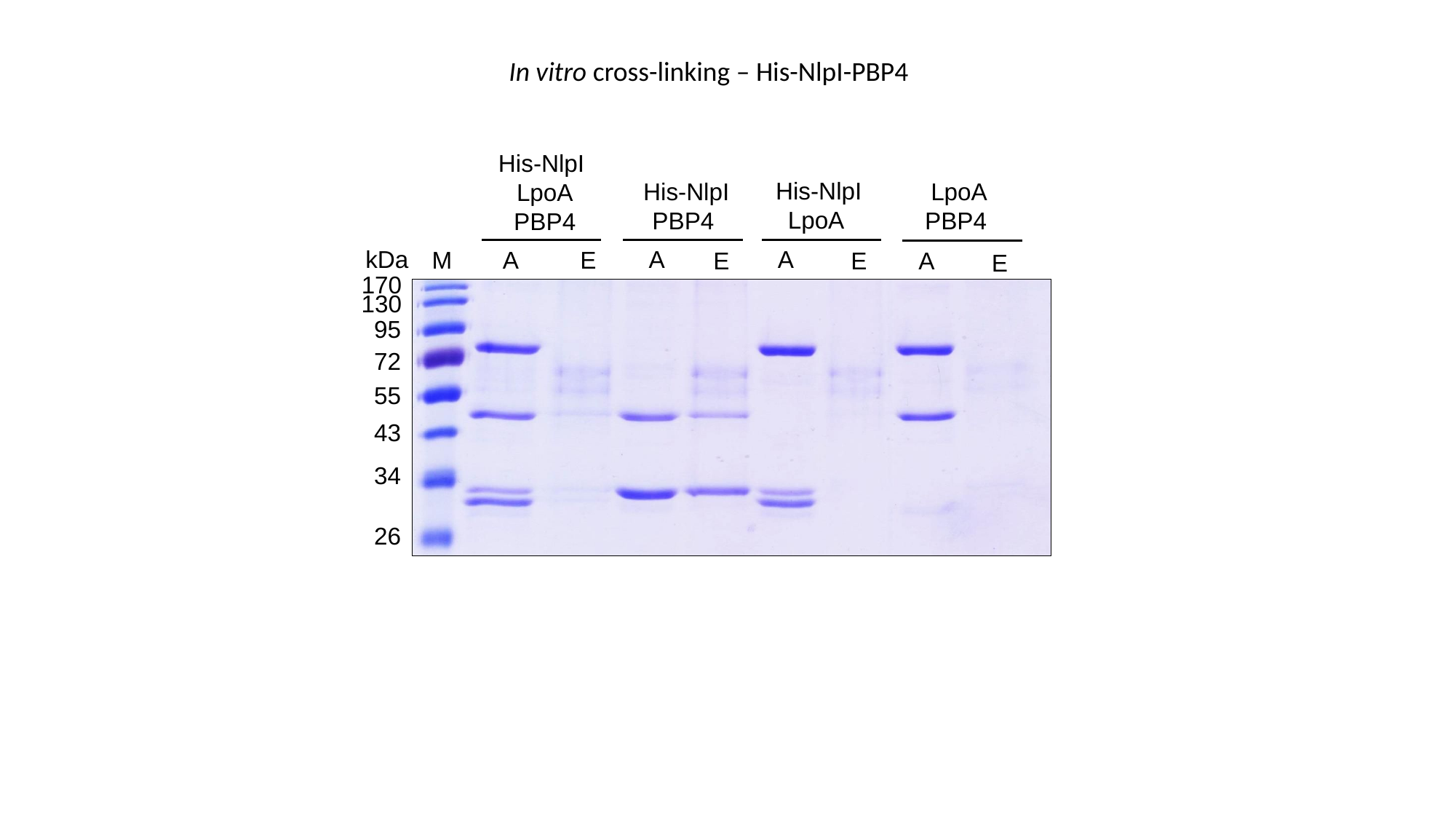

In vitro cross-linking – His-NlpI-PBP4
His-NlpI
LpoA
PBP4
His-NlpI
LpoA
His-NlpI
PBP4
LpoA
PBP4
kDa
A
A
A
M
E
E
E
A
E
170
130
95
72
55
43
34
26
